# Hybridorubrins A–D: Azaphilone Heterodimers from Stromata of *Hypoxylon fragiforme* and Insights into the Biosynthetic Machinery for Azaphilone Diversification

**DOI:** 10.1002/chem.202003215

**Published:** 2020-12-10

**Authors:** Kevin Becker, Sebastian Pfütze, Eric Kuhnert, Russell J. Cox, Marc Stadler, Frank Surup

**Affiliations:** ^1^ Department Microbial Drugs Helmholtz Centre for Infection Research GmbH (HZI) Inhoffenstrasse 7 38124 Braunschweig Germany; ^2^ German Centre for Infection Research Association (DZIF) Partner site Hannover-Braunschweig Inhoffenstrasse 7 38124 Braunschweig Germany; ^3^ Institute for Organic Chemistry Leibniz University Hannover Schneiderberg 1B 30167 Hannover Germany; ^4^ Centre for Biomolecular Drug Research (BMWZ) Schneiderberg 38 30167 Hannover Germany

**Keywords:** biosynthesis, inhibitors, natural products, polyketides, structure elucidation

## Abstract

The diversity of azaphilones in stromatal extracts of the fungus *Hypoxylon fragiforme* was investigated and linked to their biosynthetic machineries by using bioinformatics. Nineteen azaphilone‐type compounds were isolated and characterized by NMR spectroscopy and mass spectrometry, and their absolute stereoconfigurations were assigned by using Mosher ester analysis and electronic circular dichroism spectroscopy. Four unprecedented bis‐azaphilones, named hybridorubrins A–D, were elucidated, in addition to new fragirubrins F and G and various known mitorubrin derivatives. Only the hybridorubrins, which are composed of mitorubrin and fragirubrin moieties, exhibited strong inhibition of *Staphylococcus aureus* biofilm formation. Analysis of the genome of *H. fragiforme* revealed the presence of two separate biosynthetic gene clusters (BGCs) *hfaza1* and *hfaza2* responsible for azaphilone formation. While the *hfaza1* BGC likely encodes the assembly of the backbone and addition of fatty acid moieties to yield the (*R*)‐configured series of fragirubrins, the *hfaza2* BGC contains the necessary genes to synthesise the widely distributed (*S*)‐mitorubrins. This study is the first example of two distant cross‐acting fungal BGCs collaborating to produce two families of azaphilones and bis‐azaphilones derived therefrom.

## Graphical abstract

**Two become one**! Hybridorubrins A–D, unprecedented bis‐azaphilones isolated from the stromata of the ascomycete *Hypoxylon fragiforme*, are composed of mitorubrin‐ and fragirubrin‐type moieties. As revealed by genome analysis, two distantly located biosynthetic gene clusters work together to form these precursors. In contrast to their building blocks, the hybridorubrins exhibit strong inhibitory activity against biofilm formation of *Staphylococcus aureus*.

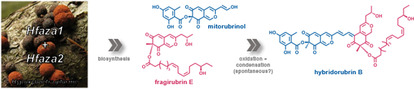

## Introduction

The Hypoxylaceae, which were recently resurrected in the course of a major phylogenetic study, are the second largest family of the ascomycete order Xylariales,^[1]^ and they are known for a particularly diverse secondary metabolism.^[2]^ In contrast to other families of the order, both their mycelial cultures and their stromata (a mass of fungal tissue that has embedded spore‐bearing structures such as ascomata) have been shown to contain diverse pigments and other secondary metabolites. The first of these pigments were reported in 1974 by Steglich et al. from *Hypoxylon fragiforme*, the type species of the largest genus of the Hypoxylaceae, and shown to belong to the mitorubrin‐azaphilone class of metabolites.^[3]^ Several years later, the same species was subjected to an intensive study, and various cytochalasans and other unknown compounds were detected and isolated from the young, growing stromata.^[4]^ In the same study, it was found that the composition of secondary metabolite profiles differs drastically during the vegetative growth period, and this points toward differential activation of secondary metabolite biosynthesis genes. From cultures of the fungus, several different metabolites such as dihydroisocoumarins,^[5]^ a dibenzoxanthenone,^[6]^ various cytochalasans,^[7]^ and small polyketides have been reported.^[8]^ Some of these metabolites were found to have prominent activities in biological systems, while others, like the complex azaphilones that were recently detected in fossil stromata of *H. fragiforme* and isolated from freshly collected material, are unprecedented compounds.^[1b]^ We have recently started to further evaluate the diversity of secondary metabolites in twelve selected species of the Hypoxylaceae for which we generated high‐quality genome sequences with the aim of establishing correlations between the biological and chemical diversity in these organisms at the genomic level.^[9]^ The ex‐epitype strain of *H. fragiforme*, the type species of the genus *Hypoxylon* and the most frequently encountered species in the Northern hemisphere, was selected for genome sequencing. As expected from the various reports on the chemical diversity of secondary metabolites, the genome harbours a great many biosynthetic gene clusters (BGCs) that putatively encode the biosynthesis of various polyketide and polyketide–peptide hybrids. We have recently reported on the identity of the cytochalasin gene cluster of this fungus and its partial heterologous expression in *Magnaporthe grisea*.^[10]^ Furthermore, we reported the occurrence of the novel azaphilones fragirubrins A–E and the bis‐azaphilones rutilins C and D in stromata of *H. fragiforme* in addition to the known mitorubrins.^[1b]^ The present study deals with the isolation and identification of azaphilone heterodimers with interesting structural and biological features, as well as the assignment of their biosynthesis genes.

## Results and Discussion

### Isolation and structure elucidation

**Figure 1:**
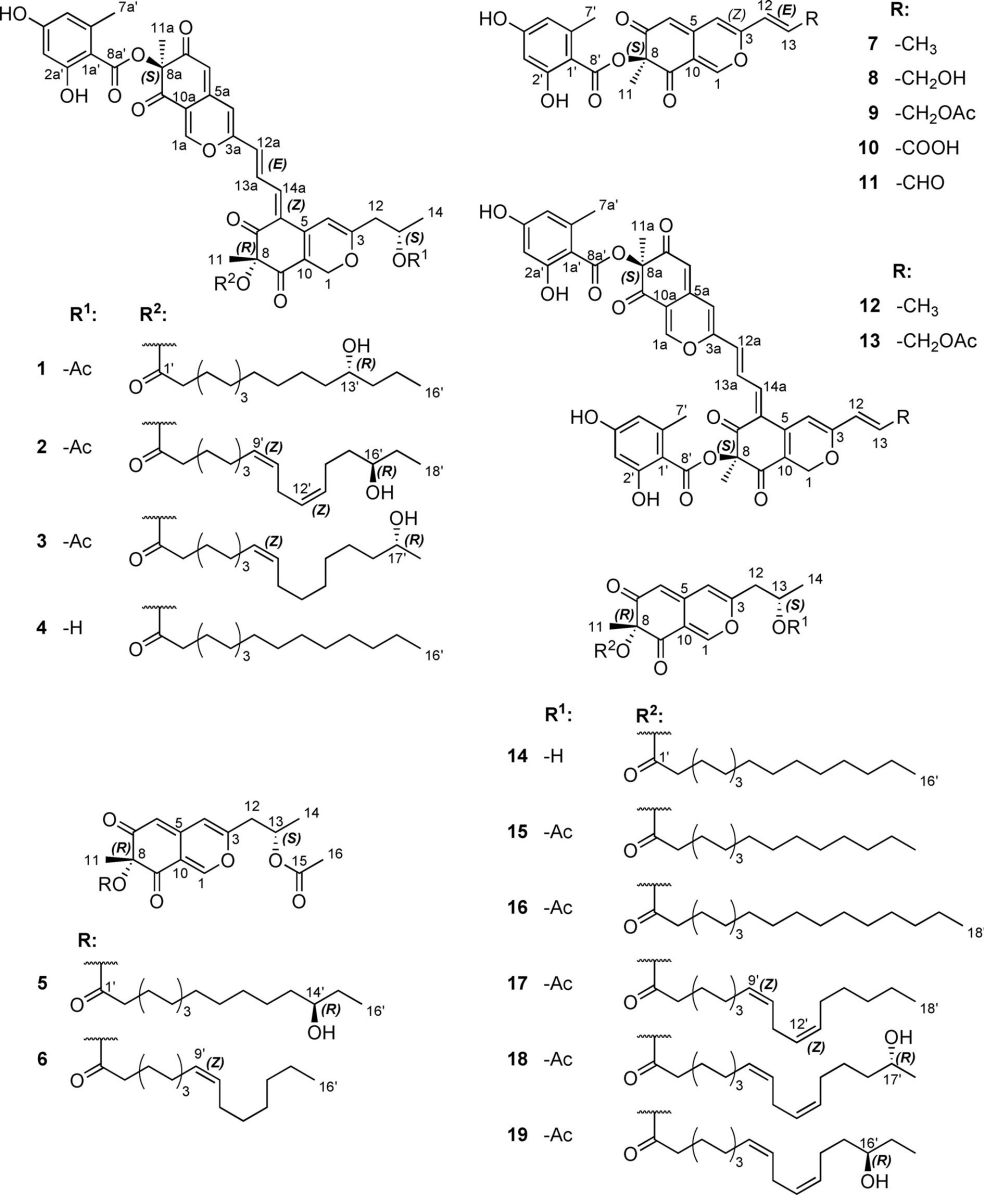
Structures of new (left) and known (right) azaphilones isolated from the stromata of *Hypoxylon fragiforme*: **1**–**4**: hybridorubrins A–D; **5**–**6**: fragirubrins F–G; **7**: mitorubrin; **8**: mitorubrinol; **9**: mitorubrinol acetate; **10**: mitorubrinic acid; **11**: mitorubrinal; **12**–**13**: rutilins C–D; **14**: lenormandin F; **15**–**19**: fragirubrins A–E.

Freshly collected stromata of *Hypoxylon fragiforme* were extracted with acetone. In the crude extract the new compounds **1**–**6** (Figure 1) were detected by HR‐ESI‐MS analysis and subsequently purified by preparative chromatography. Proton and carbon NMR data of the pure **1**–**6** are given in Tables 1 and 2.

**Table 1:** ^1^H NMR spectroscopic data [ppm] of hybridorubrins A–D (**1**–**4**) and fragirubrins F–G (**5**–**6**) (**1**, **3**–**4**, **6**: 700 MHz; **2**, **5**: 500 MHz).

Position	**1** ^[a]^	**2** ^[b]^	**3** ^[a]^	**4** ^[a]^	**5** ^[a]^	**6** ^[b]^
1	5.07, d (13.6); 4.84, d (13.6)	5.18, d (14.0); 4.87, d (14.0)	5.07, d (13.6); 4.84, d (13.6)	5.02, d (13.6); 4.94, d (13.6)	8.00, d (1.1)	7.85, s
4	6.13, s	5.71, s	6.13, s	6.09, s	6.45, s	6.14, s
6	–	–	–	–	5.47, d (1.1)	5.54, s
11	1.53, s	1.57, s	1.53, s	1.53, m	1.45, s	1.54, s
12	2.63, dd (14.4, 7.7); 2.57, dd (14.4, 5.2)	2.61, dd (14.6, 8.0); 2.49, dd (14.6, 5.0)	2.61, dd (14.3, 8.0); 2.57, dd (14.3, 5.4)	2.46, dd (14.2, 7.7); 2.41, dd (14.2, 5.2)	2.76, d (6.4)	2.69, dd (14.7, 7.3); 2.61, dd (14.7, 5.3)
13	5.19, dqd; (7.7, 6.5, 5.2)	5.25, m	5.19, m	4.10, m	5.17, tq (2×6.4)	5.17, m
14	1.30, d (6.5)	1.34, d (6.2)	1.30, d (6.2)	1.22, d (6.2)	1.31, d (6.3)	1.33, d (6.5)
16	1.99, s	2.07, s	1.99, s	–	1.98, s	2.06, s
2′	2.41, t (7.3)	2.46, t (7.7)	2.42, t (7.4)	2.41, t (7.5)	2.36, t (7.4)	2.45, t (7.4)
3′	1.62, m	1.66, m	1.62, td (7.4, 1.8)	1.61, m	1.59, tt (2×7.4)	1.63, m
4′	1.39, m	1.36, m	1.40, m	1.40, m	1.37, m	1.35, m
5′–7′	1.31, m^[c]^	1.32, m^[c]^	1.35, m^[c]^	1.29, m^[c]^	1.30, m^[c]^	1.30, m^[c]^
8′	1.31, m^[c]^	2.05, m	2.05, m^[c]^	1.29, m^[c]^	1.30, m^[c]^	2.01, m^[c]^
9′	1.31, m^[c]^	5.39, m	5.35, m^[c]^	1.29, m^[c]^	1.30, m^[c]^	5.35, m^[c]^
10′	1.31, m^[c]^	5.34, m	5.35, m^[c]^	1.29, m^[c]^	1.30, m^[c]^	5.35, m^[c]^
11′	1.45/1.32, m	2.80, dd (2×6.7)	2.05, m^[c]^	1.29, m^[c]^	1.30, m^[c]^	2.01, m^[c]^
12′	1.39, m	5.38, m	1.35, m^[c]^	1.29, m^[c]^	1.44, m; 1.33, m	1.30, m^[c]^
13′	3.50, m	5.39, dd (9.3, 4.3)	1.35, m^[c]^	1.29, m^[c]^	1.37, m	1.30, m^[c]^
14′	1.36, m	2.18, m; 2.16, m	1.35, m^[c]^	1.27, m	3.42, br s	1.27, m
15′	1.36, m	1.51, m	1.31, m	1.29, m	1.44, m; 1.37, m	1.30, m
16′	0.88, t (7.1)	3.56, m	1.38, m	0.88, t (7.1)	0.90, t (7.3)	0.89, d (6.7)
17′	–	1.49, m	3.69, dt; (10.5, 5.9)	–	–	–
18′	–	0.95, t (7.4)	1.10, d (5.9)	–	–	–
1a	8.21, s	7.98, s	8.21, d (0.9)	8.21, d (1.1)	–	–
4a	6.89, s	6.45, s	6.89, s	6.88, s	–	–
6a	5.74, d (0.9)	5.76, s	5.74, d (0.9)	5.74, d (1.1)	–	–
11a	1.68, s	1.69, s	1.68, s	1.68, m	–	–
12a	7.02, d (15.3)	6.59, d (15.3)	7.01, d (15.2)	7.04, d (15.1)	–	–
13a	8.05, dd (15.3, 11.8)	8.01, dd (15.3, 11.7)	8.05, dd (15.2, 11.6)	8.05, dd (15.1, 11.8)	–	–
14a	7.54, d (11.8)	7.02, d (11.7)	7.54, br d (11.6)	7.56, d (11.8)	–	–
3a′	6.24, d (2.4)	6.18, d (2.2)	6.23, d (2.4)	6.23, d (2.4)	–	–
5a′	6.36, m	6.21, d (2.2)	6.36, d (2.4)	6.36, m	–	–
7a′	2.61, m	2.61, m	2.61, s	2.61, s	–	–
2a′‐OH	10.74, s	10.75, s	10.74, s	10.74, s	–	–
4a′‐OH	9.24, s	n/a ^[d]^	9.26, s	9.24, s	–	–
misc.	13′‐OH; 3.22, d (5.4)	16′‐OH; n/a^[d]^	17′‐OH; 3.31, d (4.7)	13‐OH; 3.92, d (4.7)	14′‐OH; n/a^[d]^	–

[a] [D_6_]acetone. [b] CDCl_3_. [c] Signals could not be unambiguously assigned due to overlaps. [d] No signals observed.

**Table 2:** ^13^C NMR spectroscopic data [ppm] of hybridorubrins A–D (**1**–**4**) and fragirubrins F–G (**5**–**6**) (**1**, **3**–**4**, **6**: 175 MHz; **2**, **5**: 125 MHz).

Position	**1** ^[a]^	**2** ^[b]^	**3** ^[a]^	**4** ^[a]^	**5** ^[a]^	**6** ^[b]^
1	65.0, CH_2_	64.5, CH_2_	65.0, CH_2_	64.9, CH_2_	155.0, CH	153.7, CH
3	166.1, C	165.4, C	166.2, C	167.9, C	159.3, C	157.8, C
4	98.3, CH	96.5, CH	98.3, CH	97.7, CH	111.3, CH	110.8, CH
5	143.4, C	142.1, C	143.4, C	143.6, C	143.1, C	141.9, C
6	129.6, C	129.2, C	129.6, C	129.8, C	107.6, CH	107.5, CH
7	194.6, C	193.8, C	194.6, C	194.6, C	192.6, C	192.8, C
8	85.7, C	84.5, C	85.7, C	85.7, C	85.4, C	84.1, C
9	189.7, C	189.1, C	189.7, C	189.6, C	193.8, C	193.2, C
10	115.6, C ^[c]^	115.2, C	115.6, C	115.4, C	116.0, C	115.2, C
11	22.6, CH_3_	22.1, CH_3_	22.6, CH_3_	22.6, CH_3_	22.7, CH_3_	22.1, CH_3_
12	41.5, CH_2_	40.9, CH_2_	41.5, CH_2_	45.4, CH_2_	39.9, CH_2_	39.4, CH_2_
13	68.8, CH	68.2, CH	68.8, CH	45.4, CH_2_	68.4, CH	67.5, CH
14	20.4, CH_3_	20.2, CH_3_	20.4, CH_3_	24.0, CH_3_	20.0, CH_3_	19.9, CH_3_
15	170.6, C	170.6, C	170.6, C	–	170.5, C	170.2, C
16	21.2, CH_3_	21.3, CH_3_	21.2, CH_3_	–	21.1, CH_3_	21.2, CH_3_
1′	172.4, C	172.5, C	172.4, C	172.4, C	172.7, C	173.1, C
2′	33.9, CH_2_	33.2, CH_2_	33.9, CH_2_	33.9, CH_2_	33.8, CH_2_	33.2, CH_2_
3′	25.6, CH_2_	24.6, CH_2_	25.6, CH_2_	25.6, CH_2_	25.6, CH_2_	24.6, CH_2_
4′	29.8, CH_2_	28.9, CH_2_	29.9, CH_2_	29.5, CH_2_	29.7, CH_2_	28.9, CH_2_
5′–7′	30.4, CH_2_^[c]^	29.1, CH_2_^[c]^	30.1, CH_2_^[c]^	30.4, CH_2_^[c]^	29.8, CH_2_^[c]^	29.2, CH_2_^[c]^
8′	30.4, CH_2_^[c]^	27.2, CH_2_	27.9, CH_2_^[c]^	30.4, CH_2_^[c]^	30.4, CH_2_^[c]^	27.2, CH_2_^[c]^
9′	30.4, CH_2_^[c]^	130.3, CH	130.6, CH^[c]^	30.4, CH_2_^[c]^	30.4, CH_2_^[c]^	129.9, CH^[c]^
10′	30.4, CH_2_^[c]^	127.8, CH	130.6, CH^[c]^	30.4, CH_2_^[c]^	30.4, CH_2_^[c]^	129.9, CH^[c]^
11′	26.6, CH_2_	25.6, CH_2_	27.9, CH_2_^[c]^	30.4, CH_2_^[c]^	30.4, CH_2_^[c]^	27.2, CH_2_^[c]^
12′	38.7, CH_2_	128.6, CH	30.1, CH_2_^[c]^	30.4, CH_2_^[c]^	26.6, CH_2_	29.2, CH_2_^[c]^
13′	71.2, CH	129.5, CH	30.1, CH_2_^[c]^	30.4, CH_2_^[c]^	38.1, CH_2_	29.2, CH_2_^[c]^
14′	40.9, CH_2_	23.6, CH_2_	30.1, CH_2_^[c]^	32.7, CH_2_	72.9, CH	31.8, CH_2_
15′	19.7, CH_2_	23.6, CH_2_	26.7, CH_2_	23.4, CH_2_	31.2, CH_2_	22.6, CH_2_
16′	14.6, CH_3_	36.6, CH_2_	40.4, CH_2_	14.4, CH_3_	10.5, CH_3_	14.1, CH_3_
17′	–	72.9, CH	67.6, CH	–	–	–
18′	–	30.2, CH_2_	24.2, CH_3_	–	–	–
1a	155.2, CH	153.8, CH	155.4, CH	155.2, CH	–	–
3a	155.4, C	154.4, C	155.2, C	155.5, C	–	–
4a	115.8, CH	114.7, CH	115.8, CH	115.7, CH	–	–
5a	143.0, C	141.9, C	143.0, C	143.1, C	–	–
6a	110.0, CH	109.5, CH	110.0, CH	109.9, CH	–	–
7a	192.3, C	192.6, C	192.3, C	192.3, C	–	–
8a	86.8, C	84.9, C	86.8, C	86.8, C	–	–
9a	192.8, C	192.2, C	192.8, C	192.8, C	–	–
10a	115.6, C ^[c]^	114.5, C	115.6, C	115.6, C	–	–
11a	22.7, CH_3_	22.2, CH_3_	22.7, CH_3_	22.7, CH_3_	–	–
12a	135.1, CH	132.9, CH	135.1, CH	135.0, CH	–	–
13a	131.5, CH	131.1, CH	131.5, CH	131.5, CH	–	–
14a	140.2, CH	137.9, CH	140.2, CH	140.0, CH	–	–
1a′	105.0, C	104.5, C	105.0, C	105.0, C	–	–
2a′	166.2, C	165.5, C	164.0, C	166.2, C	–	–
3a′	101.8, CH	101.1, CH	101.8, CH	101.8, CH	–	–
4a′	164.0, C	161.5, C	166.2, C	164.0, C	–	–
5a′	112.7, CH	111.7, CH	112.7, CH	112.7, CH	–	–
6a′	144.9, C	144.7, C	144.9, C	144.9, C	–	–
7a′	24.1, CH_3_	24.0, CH_3_	24.1, CH_3_	24.1, CH_3_	–	–
8a′	170.7, C	169.9, C	170.7, C	170.7, C	–	–

[a] [D_6_]acetone. [b] CDCl_3_. [c] Signals could not be unambiguously assigned due to overlap.

Hybridorubrin A (**1**) was shown to have the molecular formula C_52_H_62_O_15_ by HR‐ESI‐MS. The IR spectrum of **1** showed characteristic absorptions at $\tilde{\nu }$_max_=1717 and 1621 cm^−1^, representing ester and conjugated double bonds, respectively (Figure S42 in the Supporting Information). In the ^1^H and ^1^H/^13^C HSQC NMR spectra, the presence of six methyl groups, two methylene groups, and an uncertain number of methylene groups in an alkyl chain, as well as one two aliphatic and nine aromatic/olefinic methine groups, was observed. The ^13^C and ^1^H/^13^C HMBC spectra showed the presence of four conjugated keto groups, three carboxylic ester groups, as well as eleven sp^2^‐ and two sp^3^‐hybridized carbon atoms.

**Figure 2:**
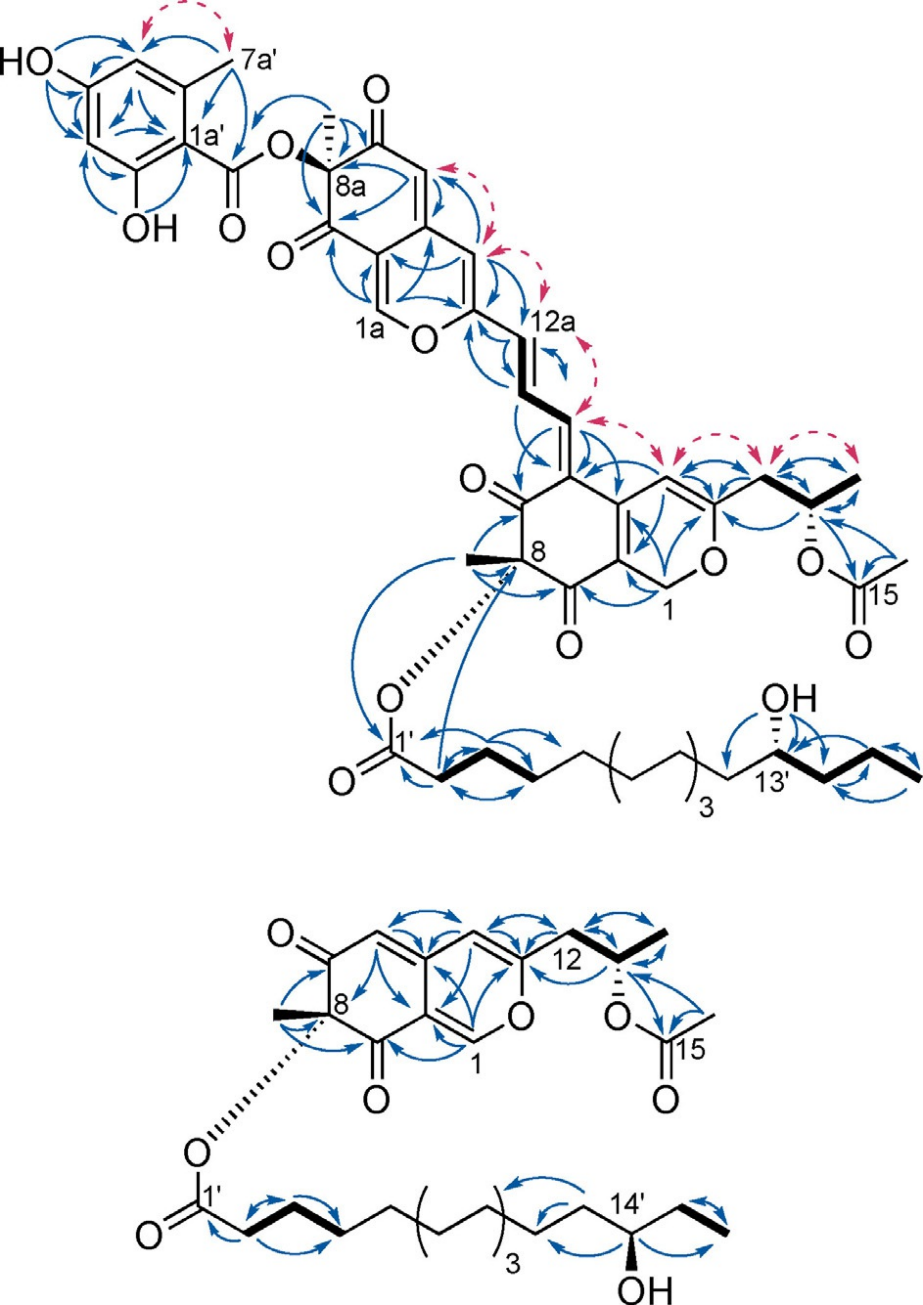
Key NMR correlations of hybridorubrin A (**1**) and fragirubrin F (**5**). Bold bonds: ^1^H/^1^H COSY correlations; solid, blue arrows: ^1^H/^13^C HMBC correlations; dashed, pink arrows: ^1^H/^1^H ROESY correlations.

^1^H/^1^H COSY signals (Figure 2) revealed 12‐H_2_, 13‐H, and 14‐H_3_ to be contiguous. For the propyl chain protons 12a‐H_2_, 13a‐H, and 14a‐H_3_, a similar link was established. The first azaphilone core was identified by ^1^H/^13^C HMBC correlations (Figure 2) from 4‐H to C‐3, C‐6, and C‐10, from 1‐H_2_ to C‐3, C‐5, C‐9, and C‐10, as well as from 11‐H_3_ to C‐7, C‐8, and C‐9. Mutual correlations of 4‐H and 12‐H_2_ linked C‐12 to C‐3. An acetate moiety was connected to C‐13 by correlations from 13‐H to C‐15 and from 16‐H_3_ to C‐12. The second azaphilone unit was established analogously. Correlations from 13_a_‐H to C‐6 as well as 14a‐H to C‐5, C‐6, and C‐7 linked the two azaphilone units. The *Z* configuration of the Δ^6,14a^ alkene was deduced from the presence of a strong ^1^H/^1^H ROESY correlation between 14a‐H and 4‐H (Figure 2 and Figure S10 in the Supporting Information), while that of the Δ^12a,13a^ alkene was determined as *E* from the coupling constant of the respective protons (^3^ *J*=15.3 Hz).

For the fatty acid moiety, the carboxylic terminus was established by ^1^H/^1^H COSY correlations linking 2′‐H_2_, 3′‐H_2_, and 4′‐H_2_ as well as by ^1^H/^13^C HMBC correlations from 3′‐H_2_ to C‐1′, C‐2′, C‐4′, and C‐5′. The methyl terminus 16′‐H_3_ showed correlations to C‐15′ and C‐14′, which were supported by ^1^H/^1^H COSY data. The hydroxyl group 13′‐OH showed ^1^H/^13^C HMBC correlations to C‐12′, C‐13′, and C‐14′. The methylene 12′‐H_2_ had a correlation to C‐11′, which was accordingly placed in the alkyl chain. The missing signals for C‐5′ to C‐10′ overlap and could not be assigned unambiguously. Consequently, the length of the fatty acid chain was deduced from the molecular formula of **1**. By using Mosher's method,^[11]^ the stereochemistry of C‐13′ was assigned as (*R*) (Figure S38 in the Supporting Information). Ultimately, the fatty acid moiety of **1** was deduced to be (*R*)‐13′‐hydroxypalmitic acid. The fatty acid was linked to C‐8 by ^4^ *J* ^1^H/^13^C HMBC correlations from 11‐H_3_ to C′‐1 and from 2′‐H_2_ to C‐8. Lastly, the orsellinic acid moiety was established by ^1^H/^13^C HMBC correlations of 7a′‐H_3_ to C‐1a′, C‐5a′, C‐6a′, and C‐8a′, from 4a′‐OH to C‐3a′, C‐4a′, and C‐5a′, as well as from 2a′‐OH to C‐1a′, C‐2a′, and C‐3a′. Correlations from 11a‐H_3_ to C‐8a′ linked the orsellinic acid to C‐8a. The stereochemistry of C‐8(*R*) and C‐8a(*S*) was deduced from their respective building blocks mitorubrin and fragirubrin (see Stereochemistry section below for details). Due to occurrence of a fragirubrin building block in **1**, the stereochemistry of C‐13(*S*) was deduced from the fragirubrins.^[1b]^ Eventually, the same C‐13(S) configuration was also found in lenormandin F.^[12]^

Analysis of hybridorubrin B (**2**) revealed its molecular formula to be C_54_H_62_O_15_, indicating two additional carbon atoms and two additional degrees of unsaturation compared with **1**. Instead of (*R*)‐13′‐hydroxypalmitic acid, it bears (*R*)‐16′‐hydroxylinoleic acid, as shown by its NMR data. The stereochemistry of C‐16′ was assigned by Mosher's method (Figure S39 in the Supporting Information). The chemical shifts of C‐8′ and C‐14′ were characteristic for a *cis* (*Z*)*/cis* (*Z*) 1,4‐diene configuration of Δ^9′,10′^ and Δ^12′,13′^.^[13]^

Hybridorubrin C (**3**) has a molecular formula of C_54_H_64_O_15_, as shown by HR‐ESI‐MS data. This implied a formal loss of hydrogen relative to **1**, representing one additional degree of unsaturation, while having a fatty acid moiety extended by two carbon atoms. Accordingly, two olefinic protons were observed in the ^1^H/^13^C HSQC spectrum and placed in the fatty acid chain of **3**. The exact position of the alkene was deduced to be Δ^9′,10′^ due to occurrence of two diagnostic MS/MS fragments (*m*/*z* 155.1123 and 171.1066) after epoxidation of the double bond (see Experimental Section and Figure S45 in the Supporting Information).^[14]^ The stereochemistry of this alkene was determined as *cis* (*Z*) from comparison of chemical shifts of the allylic carbon atoms C‐8′ and C‐11′ (both *δ*_C_=27.4).^[15]^ By applying Mosher's method, the stereochemistry of C‐17′(*R*) was deduced (Figure S40 in the Supporting Information).

Hybridorubrin D (**4**) showed a molecular formula of C_50_H_60_O_13_, implying the formal loss of a C_2_H_2_O_2_ fragment compared to **1**. The NMR spectra of **4** were highly similar to those of **1**, with the key differences being the lack of an acetyl group attached to O‐13 as well as a different fatty acid moiety, which was identified as palmitic acid.

The molecular formula of fragirubrin F (**5**) was determined from its HR‐ESI‐MS data as C_31_H_46_O_8_. Its ^1^H and ^13^C NMR data showed high similarity to those of fragirubrin A (**15**).^[1b]^ Compound **5** contains four methyl groups, three olefinic and two aliphatic methine groups, as well as 14 methylene groups. Additionally, signals for two conjugated keto groups, two ester carbonyl groups, one oxygenated sp^3^ carbon atom and three sp^2^ carbon atoms were observed in the ^13^C NMR spectra. The main difference to **15** was the replacement of the palmitoyl moiety by (*R*)‐14′‐hydroxypalmitic acid. The absolute stereochemistry of C‐14′ was determined by using Mosher's method (Figure S41 in the Supporting Information).

HR‐ESI‐MS data determined the molecular formula of fragirubrin G (**6**) as C_31_H_44_O_7_, implying one additional degree of unsaturation relative to fragirubrin A (**15**). The ^1^H and ^1^H/^13^C HSQC spectra located an additional olefin in the fatty acid moiety (Δ^9′,10′^). The position and stereochemistry of this double bond was determined by degradation of the compound to its fatty acid methyl ester (**6**‐FAME) and subsequent comparison of GC retention times with those of authentic standards, which resulted in the identification of 9‐*cis* (*Z*)‐hexadecenoic acid (palmitoleic acid; see Figure S46 in the Supporting Information and Experimental Section).

### Stereochemistry of azaphilones occurring in *H. fragiforme* and revision of rutilins C and D

The stereochemistry of the azaphilones, particularly of C‐8 in the backbone, is an important aspect of structural complexity. While the first occurrence of (−)‐mitorubrins was described in 1965 by Büchi et al. from cultures of *Penicillium rubrum* ^[16]^ (current name: *Talaromyces ruber*), Steglich et al. later described (+)‐stereoisomers of mitorubrins from the stromata of *H. fragiforme*.^[3]^ Curiously, the genus *Talaromyces* was reported to contain either (+)‐ or (−)‐mitorubrins depending on the species.^[17]^

We utilized electronic circular dichroism (ECD) spectroscopy as a means to assess the stereochemistry of the monomeric azaphilones. A study by Clark et al. on chemical synthesis of the azaphilone backbone^[18]^ allowed for relatively simple assignment: mitorubrinol (**8**) from *H. fragiforme* showed a positive cotton effect (CE) at 245 nm and negative CE at 226 and 272 nm (Figure S44 in the Supporting Information). These ECD data indicate an (*S*)‐(+)configuration,^[18]^ which we conferred to all mitorubrin‐type azaphilones from *H. fragiforme* due to their common biosynthetic origin (see Biosynthesis section for details). However, all fragirubrins^[1b]^ showed inverted ECD spectra with positive CE at about 230 and 274 nm and negative CE around 250 nm, accordingly rendering them (*R*)‐(−) isomers (Figure S44 in the Supporting Information).

Taking the ECD results and the BGC analysis (see Biosynthesis section below) into account then allows for stereochemical assignment of the heterodimers: rutilins such as **12**–**13**, which consist of two (*S*)‐mitorubrin‐type building blocks, are hence (*S*)‐configured at both C‐8 and C‐8a. Hybridorubrins **1**–**4**, in turn, are (*S*)‐configured at C‐8a in their mitorubrin moiety and (*R*)‐configured at C‐8 in their fragirubrin part. We hence have to revise data from our prior study with rutilins C (**12**) and D (**13**), and the mitorubrins,^[1b]^ to be (*S*)‐configured at C‐8 and C‐8a.

### Bioactivity testing

Compounds **1**–**2**, **4**–**10**, and **12**–**18** were tested for their antimicrobial activity in a minimum inhibitory concentration (MIC) assay as well as for their cytotoxicity, but were found to be devoid of activity against the examined test organisms or cell lines (Table S3 in the Supporting Information). The lack of antimicrobial and cytotoxic activities is largely in accordance with former findings for mitorubrin‐type azaphilones.^[4]^

In addition, **1, 3**–**4**, **7**–**10**, **12**, and **14**–**15** were tested for their inhibitory effect on biofilm formation of *Staphylococcus aureus* (Table 3). Due to minor impurities in the samples, the given percentage values only allow for a rough estimation of bioactivity. Strong activity was observed for the bis‐azaphilones hybridorubrins A (**1**), C (**3**), D (**4**), and rutilin C (**12**). These compounds have potency similar to that of the reference microporenic acid A,^[19]^ as well as sclerin, and sclerin diacid from *H. fragiforme*.^[8]^ The mitorubrin‐type azaphilones **7** and **9**, as well as the fatty acid‐containing **15**, showed weak activity, while **8**, **10**, and **14** showed no inhibition.

**Table 3:** Inhibitory effect of azaphilones from *Hypoxylon fragiforme* on biofilm formation of *Staphylococcus aureus*.

Compound	Biofilm inhibition [%]^[a]^	Concentration [μg×mL^−1^]	Potency of inhibition^[b]^
hybridorubrin A (**1**)	81	250	+++
	72	125	
	68	62.5	
	61	31.3	
	45	15.6	
	32	7.8	
	27	3.9	
hybridorubrin C (**3**)	82	250	+++
	79	125	
	65	62.5	
	60	31.3	
	60	15.6	
	34	7.8	
	25	3.9	
hybridorubrin D (**4**)	71	250	+++
	61	125	
	50	62.5	
	37	31.3	
	27	15.6	
mitorubrin (**7**)	29	250	+
	27	125	
	27	62.5	
mitorubrinol (**8**)	n.i.	250	−
mitorubrinol acetate (**9**)	24	250	+
mitorubrinic acid (**10**)	n.i.	250	−
rutilin C (**12**)	59	250	+++
	72	125	
	63	62.5	
	51	31.3	
	41	15.6	
lenormandin F (**14**)	n.i.	250	−
fragirubrin A (**15**)	27	250	+
	29	125	
	38	62.5	
microporenic acid A^[19]^	81	250	+++
	83	125	
	45	62.5	
	20	31.3

[a] Only inhibition values ≥20 % are listed here; n.i.: no inhibition. [b]+++: inhibition ≥70 %; ++: inhibition ≥40 and <70 %; +: inhibition ≥20 and <40 %; −: inhibition <20 %.

These results allow for preliminary structure–activity relationships to be deduced: since rutilin C (**12**) showed much stronger inhibition of biofilm formation than **7**, a strong influence of the fused second azaphilone backbone is suggested. In addition, the differing inhibition of biofilm formation of the mitorubrin‐type azaphilones **7**–**10** indicates a modest influence of the functional group at C‐14: a methyl group or an acetate unit (**7**, **9**) allowed for weak activity, while azaphilones carrying more polar hydroxyl or carboxylic acid moieties at C‐14 (**8**, **10**) exhibited no inhibition of biofilm formation.

Lenormandin F (**14**) and fragirubrin A(**15**), which both carry a C_16_ fatty acid moiety instead of an orsellinic acid residue at C‐8, only differ in the presence of an acetate moiety at C‐13 in **15**. While **14** showed no activity, **15** exhibited weak activity similar to **7** and **9**. Hence, a positive effect of C‐13 acetylation can be deduced. By comparing **7** and **9** to **15**, the presence of an orsellinic acid or a fatty acid moiety at C‐8 does not seem to be highly relevant for activity against *S. aureus* biofilm formation.

Taking these findings into account, the strong bioactivity measured for hybridorubrins A (**1**), C (**3**), and D (**4**) and rutilin C (**12**) can be mainly explained by the fusion of two azaphilone building blocks. As acetylation of C‐13 was deduced to be beneficial for bioactivity, **4** consequently exhibited a weaker effect than **1** and **3**.

### Azaphilone BGC analysis

In order to understand how the wide diversity of azaphilone‐type compounds in the stromata of *H. fragiforme* is genetically encoded, we investigated the genome sequence of the producer organism. Genome sequencing of the fungus had been performed in the context of a previous study.^[9]^

To identify a likely candidate gene cluster, the previously published sequences of the BGCs encoding azaphilones in *Monascus ruber* ^[20]^ (i.e., monascin, ankaflavin, and monascorubrin), azanigerones in *Aspergillus niger*,^[21]^ and mitorubrinol in *Talaromyces marneffei* ^[22]^ were used for homology searches. In *M. ruber*, *A. niger*, and *T. marneffei* assembly of the azaphilone core structure is initiated by the action of a non‐reducing polyketide synthase (NR‐PKS) and finalized by subsequent processing of a ketoreductase (KR) and FAD‐dependent monooxygenase (FAD‐MO).^[20]^ These three core proteins were initially used as the template for BLASTP searches against a *H. fragiforme* protein database created by using the software Geneious 9.1.8.

**Figure 3:**
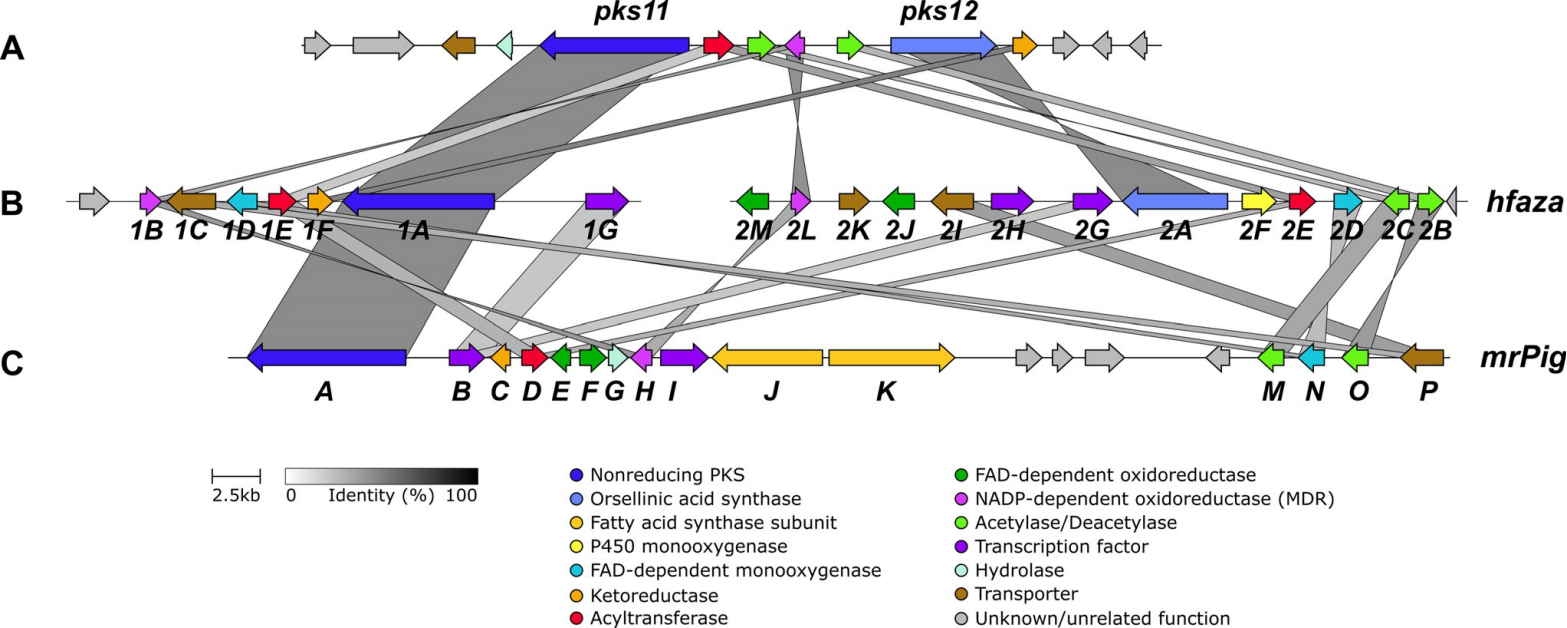
Gene cluster comparison of the BGCs encoding for azaphilone production visualized with the clinker tool: **A**, the known mitorubrin BGC of *T. marneffei*; **B**, *hfaza1* and *hfaza2* from *H. fragiforme*; **C**, the known *mrPig* BGC from *M. ruber*. In accordance with the original publication,^[22]^ no further labels were assigned to the *T. marneffei* genes.

In total, seven NR‐PKS‐containing BGCs were found. However, only one included the required KR and FAD‐MO encoding genes. This BGC (designated *hfaza1*, GenBank Acc. No. MN736721) is composed of seven genes, the majority of which show high homology with the biosynthetic genes of the *M. ruber* azaphilone *mrPig* and the *T. marneffei* mitorubrinol BGCs (Figure 3). In addition to the NR‐PKS (*hfaza1A*), the KR *(hfaza1F*), and the FAD‐MO (*hfaza1D*), genes encoding an NADPH‐dependent dehydrogenase (*hfaza1B*), an ac(et)yltransferase (*hfaza1E*), a transporter (*hfaza1C*), and a transcription factor (*hfaza1G*) are present.

A previous investigation of the mitorubrinol gene cluster in *T. marneffei* showed that two PKS genes are involved in the biosynthesis of **8** and **10**.^[22]^ The second PKS encodes the biosynthesis of orsellinic acid. We therefore searched for a homologue of the putative *T. marneffei* orsellinic acid synthase (OSAS) *pks12* in *H. fragiforme*. Accordingly, we found a gene cluster encoding a highly similar NR‐PKS together with a set of genes of which the majority also appeared in the *M. ruber* (Figure 3) and *A. niger* azaphilone BGCs. This second gene cluster is designated *hfaza2* (GenBank Acc. No. MN736720). The respective NR‐PKS (Hfaza2A) has an SAT‐KS‐AT‐PT‐ACP domain structure, and thus lacks a typical C‐terminal release domain. Additional genes in the *hfaza2* BGC encode an FAD‐MO (*hfaza2D*) with high homology to *hfaza1D* and *mrPigN*, an ac(et)yltransferase (*hfaza2E*) homologous to *hfaza1E* and *mrPigD*, a P450 monooxygenase (*hfaza2F*), and an NADPH‐dependent dehydrogenase (*hfaza2L*) with homology to *hfaza1B* and *mrPigH*. Furthermore, two similar genes were also found in the BGC (*hfaza2B* and *hfaza2C*) that did not produce any hits in BLASTP searches against the Swiss‐Prot database, but showed strong homology with the azaphilone biosynthesis genes *mrPigM* and *mrPigO* from *M. ruber*. On the basis of knockout experiments of the latter two, it was deduced that MrPigM is an acetyltransferase, whereas MrPigO performs deacetylation.^[20]^ In addition to these genes, two FAD‐dependent oxidoreductases (*hfaza2J* and *hfaza2M*) were found, which are very similar to *azaG* and *azaL*, both of which are part of the azanigerone biosynthetic pathway.^[21]^

Finally, two putative transcription factors (*hfaza2G* and *hfaza2H*) and two putative transporters (*hfaza2I* and *hfaza2K*) were also assigned to the cluster. A detailed comparison of the *hfaza1* and *hfaza2* clusters with the uncharacterized mitorubrinol BGC reported from *T. marneffei* ^[22]^ showed the presence of almost all genes from the latter in the *H. fragiforme* BGC (Figure 3). Homologues of Hfaza1A, Hfaza2A, Hfaza1B, Hfaza2B, Hfaza2C, Hfaza1E, Hfaza2E, Hfaza1F, and Hfaza2L, were found. The *T. marneffei* cluster is expanded by two hydrolase enzymes, but no FAD‐dependent monooxygenase, P450 monooxygenases, FAD‐dependent oxidoreductases, and transcription factors are present. Therefore, we propose, according to the homology analyses, that two unlinked BGCs (*hfaza1* and *hfaza2*) act together to assemble and diversify azaphilones in *H. fragiforme*.

**Scheme 1:**
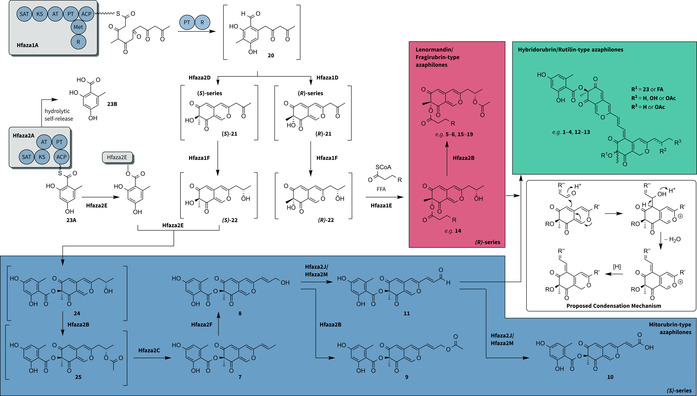
Biosynthetic hypothesis for the production and diversification of azaphilone‐type compounds in *H. fragiforme*. Putative intermediates that could not be isolated and were only detectable in traces by HPLC‐MS or not detectable at all are shown in square brackets. R in the free fatty acids (FFA) and respective side chain indicates variations in chain length, hydroxylation and unsaturation pattern depending on the final product.

*H*. *fragiforme* does not readily produce azaphilones in laboratory culture, so it is not yet possible to investigate the biosynthesis experimentally. However, there is now sufficient detailed knowledge concerning the biosynthesis of related compounds in other organisms to allow the development of a detailed biosynthetic hypothesis based on the combination of structure information and analytical HPLC‐MS data (Scheme 1).

The formation of azaphilones likely starts in a similar fashion as proved for azanigerones and *Monascus* pigments with the NR‐PKS Hfaza1A producing a hexaketide chain, which is subsequently cyclised by the product template (PT) domain and released by the reductive release (R) domain of the PKS to yield the reactive benzaldehyde intermediate **20**. Chen et al. reported that in *M. ruber* ketoreduction at C‐13 is required prior to hydroxylation at C‐8 to afford the pyran ring.^[20]^ In the crude stromatal extracts of *H. fragiforme* we could not find any evidence for the existence of such a bicyclic pyranoquinone intermediate **22**. Instead, we found a conspicuous peak with *m*/*z=*249 [*M*+H]^+^, which showed fragmentation patterns, a UV/Vis spectrum, and molecular formula consistent with the putative keto intermediate **21** (Tables S4, S5 in the Supporting Information). We therefore conclude that **21** might be produced by hydroxylation of **20** at C‐8 by the FAD‐MO Hfaza1D and subsequent spontaneous pyran ring formation. As Hfaza2D also encodes a homologous enzyme, it is possible that it can perform the same reaction. In a previous study based on crystal structure data and quantum mechanical/molecular mechanical calculations of the homologous FAD‐MO TropB, it has been demonstrated that such enzymes govern a highly enantioselective transformation.^[23]^ The occurrence of the homologous pair Hfaza1D/Hfaza2D would therefore be consistent with the observation of different stereoconfigurations at C‐8 between mitorubrin‐type and fragirubrin‐type azaphilones. Compound **21** can then be further processed by the ketoreductase Hfaza1F to yield **22**. As previously stated, we could not detect **22**, which can possibly be explained by differences in metabolic rates due to differences in enzyme reaction rates or expression levels of *hfaza1F* and subsequent processing enzymes.

In the next step, the pathway branches into two directions depending on the attached side chain. In order to yield lenormandin‐type azaphilones such as **14**, the backbone **22** can undergo acylation at the C‐8 alcohol mediated by the acyltransferase Hfaza1E. Subsequent acetylation at the C‐13 alcohol by the putative acetyltransferase Hfaza2B will lead to the highly diverse group of fragirubrins (**5**–**6**, **15**–**19**), which differ among each other in the chain length, desaturation level, and hydroxylation pattern of the side chain. This side chain very likely originates from different free or CoA‐bound fatty acids of the primary metabolism implying a broad substrate tolerance of Hfaza1E. Acyltransferases accepting a wide range of enzyme‐free acyl substrates are also involved in the biosynthesis of squalestatin.^[24]^ As only lenormandin F (**14**) has been isolated as a representative of this type of compounds from *H. fragiforme*, we assume that the majority of lenormandins are transformed into the respective fragirubrins. This hypothesis is consistent with observations made in *H. lenormandii*, which only produces the azaphilones named after this fungus.^[12]^ Thus, it can be speculated that *H. lenormandii* lacks homologues of Hfaza2B.

The diversity of mitorubrin‐type azaphilones likely starts by the attachment of orsellinic acid (**23**) to the hydroxyl group at C‐8 catalysed by Hfaza2E leading to the intermediate **24**. Due to the structural differences between **23** and fatty acids, it seems unlikely that transfer reactions are conducted by the very same enzyme. Hence, we expect the acyltransferases from both clusters to be specific for different types of substrates. In addition, we assume that these enzymes are also highly stereoselective concerning the substrate **22**, as only a single enantiomer for each compound can be detected.

Due to the absence of another obvious OSAS encoded in the genome, the involvement of Hfaza2A in the synthesis of **23** seems most likely. This is also supported by the strong homology of Hfaza2A to PKS12 of the mitorubrinol BGC in *T. marneffei*. The latter enzyme was shown to be crucial for mitorubrinol **8** ^[25]^ and mitorubrinic acid **10** ^[25]^ biosynthesis by knockdown experiments in the producing fungus, but the actual function could not be deduced from the data. Hence, it was speculated that PKS12 might be responsible for orsellinic acid biosynthesis. Unfortunately, the authors only looked specifically for the absence of **8** and **10**, but did not search for additional products in the extracts of their transformants to confirm this idea.^[22]^

The lack of a release domain in the proposed OSAS Hfaza2A could be compensated by the acyltransferase Hfaza2E, which might directly load the ACP‐bound **23A**. Such a reaction has already been suggested for the acyltransferase MrPigD, which presumably accepts ACP‐bound fatty acids in *Monascus* pigment biosynthesis^[20]^ and has been well characterised for the acyltransferase LovD involved in lovastatin biosynthesis.^[26]^ Because **23B** can also be detected as free acid in the stromatal crude extracts, we expect a hydrolytic self‐release mechanism analogously to truncated forms of the methylorcinaldehyde synthase.^[27]^ Intermediate **24** is then acetylated by the putative acetyl transferase Hfaza2B to give **25**. Mass searches for compounds **24** and **25** revealed the presence of respective traces in the stromatal crude extracts (Table S5 in the Supporting Information). Because of the very small amount of compound the corresponding relationships can, however, not be verified. The following step might involve deacetylation carried out by Hfaza2C to yield mitorubrin (**7**), which in return is hydroxylated at C‐14 putatively by the P450 monooxygenase Hfaza2F to afford one of the major stromatal metabolites, mitorubrinol (**8**).

Mitorubrinol (**8**) then acts as the starting material for the biosynthesis of **9** through the acetylation of the C‐14 alcohol performed either by Hfaza2B or a cluster‐independent acetyltransferase. In addition, **8** is also likely to be an intermediate towards mitorubrinic acid (**10**) via formation of the aldehyde mitorubrinal (**11**). We found a corresponding peak in stromatal crude extracts exhibiting the expected mass spectra (Figure S2, Tables S4, S5 in the Supporting Information). We were unable to isolate this compound, but standards of **11** obtained semisynthetically by oxidation of **8** with manganese oxide proved that the observed peak indeed corresponds to **11**.

The respective biosynthetic steps to **10** might be carried out by the action of the FAD‐dependent oxidoreductases Hfaza2J and Hfaza2M. As the *T. marneffei* BGC also encodes the production of **10**, but lacks oxidoreductase genes, a different mechanism is also possible. Interestingly, the mitorubrinol cluster of *T. marneffei* only leaves limited options to explain the conversion of **11** to **10**. The function of the highly conserved NADPH‐dependent dehydrogenase still remains obscure in all azaphilone biosynthetic pathways. Hence, it could theoretically also be involved in such oxidation steps.

Finally, we propose that the aldehyde functionality of **11** acts as an electrophile for the nucleophilic C‐6 in all *H. fragiforme* monomeric azaphilones to afford dimers of the rutilin‐ (**12**–**13**) and hybridorubrin‐type (**1**–**4**), as already postulated by Quang et al. for rutilins A and B.^[28]^ The presence of rutilins in *Hypoxylon rutilum* as major stromatal metabolites could also indicate that condensation is enzyme‐catalysed.^[28]^ However, this phenomenon could also be explained by the lack of an FAD‐dependent oxidoreductase to prevent the biosynthesis of a carboxylic acid functionality and leave the reactive aldehyde as the final enzymatic product. This is also consistent with the observation that no carboxylated azaphilones have been detected in *H. rutilum*.^[28]^ On the other hand, the mechanism could also involve radicals. The structures of the known bis‐azaphilone diazaphilonic acid^[29]^ or the azaphilone‐derived nitrogen‐containing chaetoglobins^[30]^ (Figure S3 in the Supporting Information) might possibly be formed by recombination of radicals establishing the carbon–carbon bond connecting the substructures.

When comparing the biosynthetic machinery of mitorubrins in *H. fragiforme* and *T. marneffei* various questions remain. The lack of monooxygenase genes in the cluster of the latter would prevent backbone assembly. Furthermore, monooxygenases are also very likely required to obtain mitorubrinol (**8**). Therefore, it seems likely that enzymes encoded outside of the BGC participate in the azaphilone formation of *T. marneffei*. Based on our biosynthetic hypothesis, we propose that the production of lenormandin‐type azaphilones requires only genes from *hfaza1* and thus is likely evolved earlier in these fungi. Consequently, *hfaza2* might be acquired later, for example, by horizontal gene transfer from *T. marneffei* or related fungi and has proved for the fungus to be compatible with *hfaza1*.

The existence of intertwining secondary metabolite gene clusters has already been reported for the production of the structurally distant compounds fumagillin and pseurotin A in *Aspergillus fumigatus*.^[25]^ However, these clusters were physically linked and consequently translocation of genes into neighbouring BGC can be explained by simple inversion of certain genomic regions within such a supercluster. Recently, independent gene clusters have been demonstrated to be responsible for the formation of the azaphilone azasperpyranone A in *Aspergillus terreus*. While one BGC produced the azaphilone backbone, the other BGC afforded and processed 5‐methyl orsellinic acid (5‐MOA). The respective 5‐MOA PKS contained a methyltransferase and thiolesterase domain and shared only little homology with Hfaza2A.

In addition to the elucidation of the biosynthetic pathway of azasperpyranon A, the regulatory network of the participating BGCs was deciphered by gene knockout of the encoded transcription factors (TF) and gene expression analysis. It was shown that each BGC is upregulated by a cluster‐specific TF, which in return are regulated by a third TF located in one of the BGCs.^[31]^ The regulatory network for azaphilone production in *H. fragiforme* could be highly similar, as three TFs have been identified across *hfaza1* and *hfaza2*. We thus tried to experimentally link *hfaza1* and *hfaza2* with the known azaphilones by ectopic overexpression of the individual TF genes using previously described methods.^[32]^ However, this proved unsuccessful. Knockout strategies are not viable in the Hypoxylaceae, as azaphilones are exclusively formed during stromatal development, which cannot be induced under laboratory conditions.

We could also find highly similar homologues of the two clusters in the taxonomically related fungus *H. rickii* and the more distantly related *H. rubiginosum* (data not shown), which are known to produce mitorubrins and/or the closely‐related rubiginosins.^[33]^ This observation further supports our theory about azaphilone biosynthesis in *H. fragiforme* and enables further options to study the pathways in detail. However, it will be a special challenge to obtain final proof of the biosynthetic mechanism, because the stromata can presently not be grown in the laboratory, and hence the only path forward would be heterologous expression.

## Conclusion

We used a combination of classical natural product chemistry and state‐of‐the‐art genome sequencing to deduce the biosynthesis of azaphilone pigments in *H. fragiforme*, demonstrating the powerful combination of those two methods. We showed that both possible C‐8 stereoisomers of azaphilones are produced in the stromata, which allows for assignment to subgroups: 1) the C‐8(*R*)‐configured azaphilones consist of the acyl‐carrying lenormandins and fragirubrins; 2) the group of C‐8(*S*)‐configured azaphilones carry an orsellinic acid moiety and belong to the family of mitorubrins and their fusion products, rutilins; and 3) the novel hybridorubrins, which are of mixed stereochemistry, as their building blocks originate from groups 1 and 2. Furthermore, the hybridorubrins A (**1**), C (**3**), and D (**4**) exhibited high bioactivity against formation of *S. aureus* biofilms.

Examination of the *H. fragiforme* genome revealed two BGCs to be most likely responsible for biosynthesis of azaphilone polyketides. The *hfaza1* BGC is likely responsible for biosynthesis of the azaphilone backbone and addition of fatty acid moieties to yield group 1 compounds. In parallel, the *hfaza2* BGC synthesizes orsellinic acid, which is esterified to the backbone to yield group 2 azaphilones and tailors the gained mitorubrins to obtain a high diversity of derivatives. We suggest that a spontaneous aldol condensation reaction is responsible for the formation of hybridorubrin and rutilin bis‐azaphilones from reactive aldehyde intermediates in *H. fragiforme*; however, this needs experimental verification. These results reveal the first example of two distant, cross‐acting BGCs that enable a large diversity of azaphilone products through natural mix‐and‐match strategies.

## Experimental Section

### General

NMR spectra were recorded with an Avance III 700 spectrometer with a 5 mm TCI cryoprobe (^1^H 700 MHz, ^13^C 175 MHz) and an Avance III 500 spectrometer (^1^H 500 MHz, ^13^C 125 MHz) (both Bruker, Billerica, MA/USA). Optical rotations were taken with a MCP 150 polarimeter (Anton Paar, Graz, Austria) and UV spectra with a UV‐2450 UV/Vis spectrophotometer (Shimadzu, Kyoto, Japan). IR spectra were taken with a Spectrum 100 FTIR spectrometer (Perkin Elmer, Waltham, MA/USA) and ECD spectra were measured using a J‐815 spectropolarimeter (Jasco, Pfungstadt, Germany).

ESI mass spectra were recorded with an UltiMate 3000 Series uHPLC (Thermo Fisher Scientific, Waltman, MA/USA) by utilizing a C18 Acquity UPLC BEH column (50×2.1 mm, 1.7 μm; Waters, Milford, USA) connected to an amaZon speed ESI‐Iontrap‐MS (Bruker, Billerica, MA, USA). HPLC parameters were set as follows: solvent A: H_2_O+0.1 % formic acid, solvent B: acetonitrile (MeCN)+0.1 % formic acid, gradient: 5 % B for 0.5 min, increasing to 100 % B over 19.5 min, keeping 100 % B for a further 5 min, flow rate 0.6 mL min^−1^, and DAD detection 190–600 nm.

ESI‐HRMS was performed with an Agilent 1200 Infinity Series HPLC (Agilent Technologies, Böblingen, Germany; conditions as for ESI‐MS) connected to a maXis ESI‐TOF‐MS (Bruker).

### Fungal material and extraction

To generate crude extract 1 air‐dried stromata (fruiting bodies, ca. 65 g) of *Hypoxylon fragiforme* were collected in 2017 from *Fagus sylvatica* in the vicinity of Braunschweig, Germany, by Lucile Wendt. Extraction was performed by adding 500 mL of acetone, followed by ultrasonication at 40 °C for 1 h. This procedure was repeated twice. The extracts were combined and dried in vacuo, which led to approximately 6 g of crude extract 1. For crude extract 2, about 25 g of air‐dried stromata of *Hypoxylon fragiforme* were collected in 2016 from *Fagus sylvatica* in the vicinity of Lake Starnberg, Germany, by Lucile Wendt. Extraction was performed as described above. This yielded approximately 3 g of crude extract 2.

### Isolation of secondary metabolites 1–6

Crude extract 1 yielded hybridorubrins A, B (**1**, **2**) and fragirubrins F, G (**5** and **6**), while hybridorubrins C, D (**3**, **4**) could be detected but not isolated to purity. Thus, extract 2 was utilised to isolate **3** and **4**, while **1** was isolated again as a by‐product.

Initially, crude extract 1 was separated by a Reveleris X2 Flash Chromatography system (Büchi, Essen, Germany). A 40 g silica cartridge (120×30 mm, 40 μm, SN 145146132, W.R. Grace, Columbia, MD/USA) was loaded with the crude extract and eluted with a ternary gradient (solvent A: CH_2_Cl_2_, B: CH_2_Cl_2_:acetone 9:1, C: acetone) as follows: at a flow rate of 40 mL min^−1^, isocratic conditions at 100 % A were set for 4 min, followed by a gradient to 100 % B over 25 min. This was followed by an increase of solvent C to 100 % over 20 min. This led to six fractions (*t*_R_ fraction I: 3.4–14.3 min, II: 14.7–15.9 min, III: 16.6–20.4 min, IV: 21–25.4 min, V: 26–29.8 min, VI: 30.3–52.2 min), which were evaporated to dryness in vacuo at 40 °C.

Fractions I, II, and V (see below) were further processed by using a preparative HPLC system (Gilson, Middleton, WI/USA; GX‐271 Liquid Handler with a GX Direct Injection Module, DAD 172, 305 and 306 Pump, 806 Manometric Module 811D Dynamic Mixer, 402 Syringe Pump). A Nucleodur C18ec column (150×40 mm, 7 μm; Macherey‐Nagel, Düren, Germany) was used at a flow rate of 40 mL min^−1^ with solvent A: H_2_O+0.1 % formic acid and solvent B: MeCN+0.1 % formic acid. After evaporation of MeCN in vacuo, the aqueous residues were frozen and freeze‐dried with an Alpha 1–4 LSC freeze dryer (Christ, Osterode, Germany). Fractions I (2×70 mg) and II (3×100 mg) were separated with a gradient under isocratic conditions for 5 min at 60 % B, followed by an increase to 100 % B over 45 min, 5 min of isocratic conditions, and, ultimately, a decrease to 60 % B over 2 min. Fragirubrin G (**6**, 0.9 mg) was gained from fraction I, while fragirubrin F (**5**, 21.6 mg) was isolated from fraction II. Fraction V (1×100 mg) was separated by using a gradient from 45 to 80 % B over 40 min, followed by an increase to 100 % B over 5 min and isocratic conditions at 100 % B for 15 min. It yielded hybridorubrin B (**2**, 2.8 mg). Fraction VI (1×40 mg) was separated with an RP‐MPLC system (Kronlab, Sinsheim, Germany; column 480×30 mm, ODS/AQ C18, solvents as described for fractions I–V) at a flow rate of 30 mL min^−1^. Starting with isocratic conditions at 10 % B for 5 min, a gradient to 100 % B over 60 min was followed by isocratic conditions at 100 % B for 30 min. Hybridorubrin A (**1**, 4.1 mg) was obtained from this separation.

Crude extract 2 was separated by using the aforementioned Reveleris X2 flash chromatography system. A 120 g C18 cartridge (200×40 mm, 40 μm, SN 5152991, Grace) was loaded with the crude extract and eluted with a binary gradient (solvent A: H_2_O+0.1 % formic acid; solvent B: MeCN+0.1 % formic acid) as follows: flow rate: 80 mL min^−1^, isocratic conditions at 5 % B for 3 min, followed by an increase to 45 % B over 1 min. This was followed by an increase to 80 % B over 10 min. Subsequently, the gradient was increased to 100 % B over 25 min. This was kept for a further 20 min. This yielded fractions I (*t*_R_: 40–45 min) and II (52.5–70 min). Both fractions I (1×180.3 mg) and II (1×122 mg), were individually processed by manual NP column chromatography. For this, the material was adsorbed on silica bulk material (63–200 μm) and transferred to a loading cartridge (SN 8634349, Grace). Downstream of that, a 12 g silica cartridge (SN 5146131, Grace) was installed. Under a vacuum of approximately 50 mbar, the extract was separated by using the following solvents: A: *n*‐heptane, B: CH_2_Cl_2_, C: MeOH. A step gradient with 100 mL of the following solvent mixtures was gradually applied: i) 20:80:0 (% A:B:C, v/v/v), ii) 0:100:0, iii) 0:99:1, iv) 0:98:2, v) 0:96:4, vi) 0:93:7, vii) 0:90:10, viii) 0:85:15, ix) 0:80:20. Each solvent mixture was loaded onto the device and the eluent gathered before another step was applied. Thus, chromatographic separation of fraction I yielded fraction v, while fraction II yielded fractions vi and vii, which were combined. Fraction v (2×10.5 mg) was further separated with a PLC 2250 HPLC system (Gilson) equipped with an X‐Bridge C18 column (250×19 mm, 5 μm, Waters), solvents A: H_2_O+0.1 % formic acid and B: MeCN+0.1 % formic acid, and the following gradient: flow rate: 20 mL min^−1^, isocratic conditions at 40 % B for 5 min, followed by an increase to 75 % B over 5 min. This was followed by an increase to 100 % B over 50 min. This yielded hybridorubrin D (**4**, 1.7 mg). Fraction vi+vii (8×11 mg) was separated by using the PLC 2250 with the conditions as described above. First, isocratic conditions at 40 % B were applied for 5 min, followed by an increase to 75 % B over 5 min. Then, 75 % B was kept for 25 min. The same fractions of the eight separations were combined according to LCMS results, which yielded hybridorubrin A (**1**, 11.9 mg) and a yet‐impure hybridorubrin C (**3**). The latter (1×4.7 mg) was further purified by again using the PLC 2250 under the same conditions as before, but with the exception of applying 77 % instead of 75 % B. This yielded hybridorubrin C (**3**, 1.7 mg).

### Physicochemical data

Hybridorubrin A (**1**): red oil; [*α*]_D_=+340 (*c*=0.02, MeCN); ^1^H NMR ([D_6_]acetone, 700 MHz): see Table 1; ^13^C NMR ([D_6_]acetone, 175 MHz): see Table 2; IR (ATR): $\tilde{\nu }$_max_=2927, 2854, 1717, 1621, 1261 cm^−1^, see Figure S42 in the Supporting Information; UV/Vis (acetone): *λ*_max_ (*ϵ*)=215 (4.54), 268 (4.40), 360 (4.60), 441 (4.37) nm; ECD (MeCN) *λ*(Δ*ϵ*)=231 (−5.0), 265 (−0.6), 293 (−5.4), 360 (+5.3) nm, see Figure S43 in the Supporting Information; ESI‐MS: *m*/*z* 927.58 [*M*+H]^+^, 925.55 [*M*−H]^−^; HR‐ESI‐MS: *m*/*z* 927.4163 [*M*+H]^+^ (calcd for C_52_H_63_O_15_: 927.4161); *t*_R_=16.3 min.

Hybridorubrin B (**2**): red oil; [*α*]_D_=+580 (*c*=0.02, MeCN); ^1^H NMR (CDCl_3_, 500 MHz): see Table 1; ^13^C NMR (CDCl_3_, 125 MHz): see Table 2; IR (ATR): $\tilde{\nu }$_max_=2929, 2855, 1717, 1630, 1263 cm^−1^, see Figure S42; UV/Vis (MeCN): *λ*_max_ (*ϵ*)=213 (4.50), 268 (4.37), 362 (4.52), 441 (4.31) nm; ECD (MeCN) *λ*(Δ*ϵ*): 198 (+5.4), 231 (−8.9), 267 (−1.7), 290 (−8.7), 355 (+8.0) nm, see Figure S43 in the Supporting Information; ESI‐MS: *m*/*z* 951.49 [*M*+H]^+^, 949.48 [*M*−H]^−^; HR‐ESI‐MS: *m*/*z* 951.4154 [*M*+H]^+^ (calcd for C_54_H_63_O_15_, 951.4161); *t*_R_=16.4 min.

Hybridorubrin C (**3**): red oil; [*α*]_D_=+355 (*c*=0.02, MeCN); ^1^H NMR ([D_6_]acetone, 700 MHz): see Table 1; ^13^C NMR ([D_6_]acetone, 175 MHz): see Table 2; IR (ATR): $\tilde{\nu }$_max_=2930, 2855, 1717, 1630, 1263 cm^−1^, see Figure S42 in the Supporting Information; UV/Vis (MeCN): *λ*_max_ (*ϵ*)=215 (4.4), 269 (4.2), 364 (4.4), 443 (4.1) nm; ECD (MeCN) *λ* (Δ*ϵ*): 197 (−7.2), 206 (−3.1), 213 (−8.3), 218 (−2.2), 232 (−12.7), 267 (+1.7), 289 (−11.0), 352 (+8.9) nm, see Figure S43 in the Supporting Information; ESI‐MS: *m*/*z* 953.52 [*M*+H]^+^, 951.48 [*M*−H]^−^; HR‐ESI‐MS: *m*/*z* 953.4321 [*M*+H]^+^ (calcd for C_54_H_65_O_15_, 953.4318); *t*_R_=16.6 min.

Hybridorubrin D (**4**): red oil; [*α*]_D_=+173 (*c*=0.015, MeCN); ^1^H NMR ([D_6_]acetone, 700 MHz): see Table 1; ^13^C NMR ([D_6_]acetone, 175 MHz): see Table 2; IR (ATR): $\tilde{\nu }$_max_=2924, 2854, 1721, 1622, 1262 cm^−1^, see Figure S42; UV/Vis (MeCN): *λ*_max_ (*ϵ*)=214 (4,36), 266 (4.11), 337 (4.12) nm; ECD (MeCN) *λ*(Δ*ϵ*): 209 (+2.6), 228 (−2.4), 268 (−0.5), 296 (−4.5), 330 (+2.7) nm, see Figure S43 in the Supporting Information; ESI‐MS: *m*/*z* 869.43 [*M*+H]^+^, 867.39 [*M*−H]^−^; HR‐ESI‐MS: *m*/*z* 869.4110 [*M*+H]^+^ (calcd for C_50_H_60_O_13_, 869.4107); *t*_R_=18.7 min.

Fragirubrin F (**5**): yellow oil; [*α*]_D_=−10 (*c*=0.1, MeCN); ^1^H NMR ([D_6_]acetone, 500 MHz): see Table 1; ^13^C NMR ([D_6_]acetone, 125 MHz): see Table 2; IR (ATR): $\tilde{\nu }$_max_=2925, 2854, 1737, 1715, 1639, 1233 cm^−1^, see Figure S42 in the Supporting Information; UV/Vis (MeCN): *λ*_max_ (*ϵ*)=220 (4.18), 326 (4.22) nm; ECD (MeCN) *λ*(Δ*ϵ*): 199 (−8.1), 232 (+1.9), 248 (−1.6), 273 (+5.2), 323 (−5.3) nm, see Figure S44 in the Supporting Information; ESI‐MS: *m*/*z* 547.34 [*M*+H]^+^, 545.28 [*M*−H]^−^; HR‐ESI‐MS: *m*/*z* 547.3272 [*M*+H]^+^ (calcd for C_31_H_47_O_8_, 547.3265); *t*_R_=14.4 min.

Fragirubrin G (**6**): yellow oil; [*α*]_D_=−2 (*c*=0.1, MeCN); ^1^H NMR (CDCl_3_, 700 MHz): see Table 1; ^13^C NMR (CDCl_3_, 175 MHz): see Table 2; IR (ATR): $\tilde{\nu }$_max_=2924, 2854, 1737, 1717, 1641, 1233 cm^−1^, see Figure S42; UV/Vis (MeCN): *λ*_max_ (*ϵ*)=218 (4.23), 327 (4.20) nm; ECD (MeCN) *λ*(Δ*ϵ*): 199 (−5.3), 232 (+1.4), 249 (−0.9), 272 (+3.8), 321 (−3.8) nm, see Figure S44 in the Supporting Information; ESI‐MS: *m*/*z* 529.38 [*M*+H]^+^, 527.24 [*M*−H]^−^; HR‐ESI‐MS: *m*/*z* 529.3160 [*M*+H]^+^ (calcd for C_31_H_45_O_7_, 529.3160); *t*_R_=17.5 min.

### Mosher's analyses

For the preparation of the (*S*)‐MTPA ester 1 mg of hybridorubrin A (**1**) was dissolved in 600 μL of [D_5_]pyridine, and 10 μL of (*R*)‐MTPA chloride was added. The mixture was incubated at 25 °C for 15 min and ^1^H NMR, ^1^H/^1^H COSY, ^1^H/^13^C‐HSQC, and ^1^H/^13^C‐HMBC spectra were measured. ^1^H NMR (700 MHz, [D_5_]pyridine): similar to **1**, but *δ*_H_=1.22 (11′‐H_2_), 1.60 (12′‐H_2_), 5.29 (13′‐H), 1.63 (14′‐H_2_), 1.37 (15′‐H_2_), 0.88 ppm (16′‐H_3_). The (*R*)‐MTPA ester was prepared in the same manner by addition of 10 μL of (*S*)‐MTPA chloride: ^1^H NMR (700 MHz, [D_5_]pyridine): similar to that of **1**, but *δ*_H_=1.67 (12′‐H_2_), 5.30 (13′‐H), 1.57 (14′‐H_2_), 1.23 (15′‐H_2_), 0.82 ppm (16′‐H_3_). Results are depicted in Figure S38 in the Supporting Information.

Hybridorubrin B (2×0.7 mg, **2**) was converted analogously. (*S*)‐MTPA ester of **2**: ^1^H NMR (700 MHz, [D_5_]pyridine): similar to **2**, but *δ*_H_=2.09 (14′‐H_2_), 1.69 (15′‐H_2_), 5.23 (16′‐H), 1.68 (17′‐H_2_), 0.91 ppm (18′‐H_3_). (*R*)‐MTPA ester of **2**: ^1^H NMR (700 MHz, [D_5_]pyridine): similar to that of **2**, but *δ*_H_=2.24 (14′‐H_2_), 1.75 (15′‐H_2_), 5.24 (16′‐H), 1.62 (17′‐H_2_), 0.80 ppm (18′‐H_3_). Results are depicted in Figure S39 in the Supporting Information.

Hybridorubrin C (2×0.5 mg, **3**) was converted analogously. (*S*)‐MTPA ester of **3**: ^1^H NMR (700 MHz, [D_5_]pyridine): similar to **3**, but *δ*_H_=1.20 (15′‐H_2_), 1.59/1.47 (16′‐H_2_), 5.27 (17′‐H), 1.30 ppm (18′‐H_3_). (*R*)‐MTPA ester of **3**: ^1^H NMR (700 MHz, [D_5_]pyridine): similar to **3**, but *δ*_H_=1.33 (15′‐H_2_), 1.67/1.52 (16′‐H), 5.26 (17′‐H_2_), 1.24 ppm (18′‐H_3_). Results are depicted in Figure S40 in the Supporting Information.

Fragirubrin F (2×0.5 mg, **5**) was converted analogously. (*S*)‐MTPA ester of **5**: ^1^H NMR (700 MHz, [D_5_]pyridine): similar to **5**, but *δ*_H_=1.21 (12′‐H_2_), 1.69/1.57 (13′‐H_2_), 5.18 (14′‐H), 1.66 (15′‐H_2_), 0.92 (16′‐H_3_) ppm. (*R*)‐MTPA ester of **5**: ^1^H NMR (700 MHz, [D_5_]pyridine): similar to **5**, but *δ*_H_=1.36 (12′‐H_2_), 1.61/1.54 (13′‐H_2_), 5.19 (14′‐H), 1.36 (15′‐H_2_), 0.81 (16′‐H_3_) ppm. Results are depicted in Figure S41 in the Supporting Information.

### Epoxidation and MS/MS measurements of 3

To locate the position of the double bond in the fatty acid moiety of hybridorubrin C (**3**), a sample was epoxidized with *meta*‐chloroperoxybenzoic acid (mCPBA) followed by MS/MS analysis, as recently published:^[14]^ at first, 10 μg of **3** was incubated with 10 μg of mCPBA in 10 μL of CH_2_Cl_2_ at room temperature. The reaction was quenched after 10 min with 490 μL of CH_2_Cl_2_:MeCN (1:1). The same procedure was applied to an authentic reference sample of *cis*‐octadecenoic acid [C18:1(9)].

Then, 1 μL of the samples was injected into an UltiMate 3000 Series uHPLC (Thermo Fisher Scientific) equipped with a C18 Kinetex column (1,7 μm, 150×2.1 mm, Phenomenex, Torrance, CA/USA) and the following gradient of H_2_O+0.1 % formic acid (A) and MeCN+0.1 % formic acid (B): 1 % B for 2 min, increasing to 100 % B over 18 min, keeping 100 % B for further 4 min, flow rate 0.3 mL min^−1^. This HPLC was connected to a maXis HD UHR‐ESI‐QTOF‐MS (Bruker) with the following parameters: scan range: *m*/*z* 50–1500, ion polarity: negative, capillary voltage: 4500 V, nebulizer pressure: 4.0 bar, dry heater: 200 °C, dry gas: 9.0 L min^−1^, collision energy: 20.3–50.7 eV. Results are depicted in Figure S45.

### GC analysis of 6

To determine the double‐bond geometry of the palmitoleic acid moiety of fragirubrin G (**6**), 0.5 mg of the compound was hydrolysed by incubating it with MeOH/NaOH (15 %) 1:1 for 1 h at 100 °C to yield **6**‐fatty acid (**6**‐FA). Then, **6**‐FA, as well as of 9‐*cis*‐ and 9‐*trans*‐hexadecenoic acid references, were derivatised to yield fatty acid methyl esters (FAMEs) by incubating them in MeOH/HCl (37 % w/v) 5:1 for 10 min at 80 °C. Afterwards, the three samples were extracted into the organic phase as described previously.^[34]^ The samples were analysed by gas chromatography with an Agilent 6890N GC with flame ionization detector. Separation of the FAMEs was carried out with a Macherey‐Nagel Optima 5 column (5 % phenyl, 95 % dimethylpolysiloxane; 50 m length; 0.32 mm inner diameter; 0.25 μm film thickness). The retention time of **6**‐FAME was compared with those of both references to identify its double‐bond configuration. The result is depicted in Figure S46 in the Supporting Information.

### Bioassays

Minimum inhibitory concentrations (MICs) were determined as described previously.^[35]^ A detailed protocol is given in the Supporting Information. Various test organisms of fungal and bacterial origin were tested. Bacteria: *Bacillus subtilis* (DSM10), *Staphylococcus aureus* (DSM346), *Micrococcus luteus* (DSM1790), *Chromobacterium violaceum* (DSM30191), *Escherichia coli* (DSM1116), *Pseudomonas aeruginosa* (PA14); Mycobacteria: *Mycolicibacterium smegmatis* (ATCC700084); Fungi: *Candida albicans* (DSM1665), *Schizosaccharomyces pombe* (DSM70572), *Mucor hiemalis* (DSM2656), *Pichia anomala* (DSM6766), *Rhodotorula glutinis* (DSM10134). Results are listed in Table S3 in the Supporting Information.

The cytotoxicity assay against mouse fibroblast cell line L929 and human cervical cancer cell line KB 3.1 was performed as described before.^[36]^ Results are depicted in Table S3 in the Supporting Information.

The biofilm formation inhibition assay against *Staphylococcus aureus* (DSM1104) was conducted as described before.^[37]^ Results are listed in Table 3.

### Bioinformatic analysis for gene cluster prediction

The genome of the *H. fragiforme* strain MUCL 51264 was sequenced by using PacBio, and gene prediction and annotation were carried out as previously described.^[10]^ Candidate gene clusters were manually identified by blastp searches by using various protein sequences as templates (UniProtKB/Swiss‐Prot: G3XMC4, G3XMC1, G3XMB9) against a created *H. fragiforme* protein database. The searches were performed with the software Geneious 9.1.8 (https://www.geneious.com). Homology between related biosynthetic gene cluster was mapped and visualized with the clinker tool.^[38]^ The identified gene cluster were uploaded to GenBank under the accession numbers MN736720 (*hfaza2*) and MN736721 (*hfaza1*).

## Conflict of interest

The authors declare no conflict of interest.

## Supporting information

As a service to our authors and readers, this journal provides supporting information supplied by the authors. Such materials are peer reviewed and may be re‐organized for online delivery, but are not copy‐edited or typeset. Technical support issues arising from supporting information (other than missing files) should be addressed to the authors.

Supplementary

## References

[1a] L. Wendt , E. B. Sir , E. Kuhnert , S. Heitkämper , C. Lambert , A. I. Hladki , A. I. Romero , J. J. Luangsa-ard , P. Srikitikulchai , D. Peršoh , M. Stadler , Mycol. Prog. 2018, 17, 115–154;

[1b] F. Surup , A. Narmani , L. Wendt , S. Pfütze , R. Kretz , K. Becker , C. Menbrivès , A. Giosa , M. Elliott , C. Petit , M. Rohde , M. Stadler , Fungal Divers. 2018, 92, 345–356.

[2] S. E. Helaly , B. Thongbai , M. Stadler , Nat. Prod. Rep. 2018, 35, 992–1014. 29774351 10.1039/c8np00010g

[3] W. Steglich , M. Klaar , W. Furtner , Phytochemistry 1974, 13, 2874–2875.

[4] M. Stadler , D. N. Quang , A. Tomita , T. Hashimoto , Y. Asakawa , Mycol. Res. 2006, 110, 811–820. 16876700 10.1016/j.mycres.2006.03.013

[5] J. R. Anderson , R. L. Edwards , A. J. S. Whalley , J. Chem. Soc. Perkin Trans. 1 1983, 2185–2192.

[6] R. L. Edwards , V. Fawcett , D. J. Maitland , R. Nettleton , L. Shields , A. J. S. Whalley , J. Chem. Soc. Chem. Commun. 1991, 1009–1010.

[7a] R. Kretz , L. Wendt , S. Wongkanoun , J. J. Luangsa-Ard , F. Surup , S. E. Helaly , S. R. Noumeur , M. Stadler , T. E. B. Stradal , Biomolecules 2019, 9, 73; 10.3390/biom9020073 PMC6406453 30791504

[7b] J. Ondeyka , O. D. Hensens , D. Zink , R. Ball , R. B. Lingham , G. Bills , A. Dombrowski , M. Goetz , J. Antibiot. 1992, 45, 679–685. 10.7164/antibiotics.45.679 1624370

[8] K. T. Yuyama , C. Chepkirui , L. Wendt , D. Fortkamp , M. Stadler , W. R. Abraham , Microorganisms 2017, 5, 80. 10.3390/microorganisms5040080 PMC5748589 29231891

[9] D. Wibberg , M. Stadler , C. Lambert , B. Bunk , C. Spröer , C. Rückert , J. Kalinowski , R. J. Cox , E. Kuhnert , Fungal Divers. 2020, 10.1007/s13225-020-00447-5.

[10] C. Wang , K. Becker , S. Pfütze , E. Kuhnert , M. Stadler , R. J. Cox , E. Skellam , Org. Lett. 2019, 21, 8756–8760. 31644300 10.1021/acs.orglett.9b03372

[11] T. R. Hoye , C. S. Jeffrey , F. Shao , Nat. Protoc. 2007, 2, 2451–2458. 17947986 10.1038/nprot.2007.354

[12] E. Kuhnert , F. Surup , E. B. Sir , C. Lambert , K. D. Hyde , A. I. Hladki , A. I. Romero , M. Stadler , Fungal Divers. 2015, 71, 165–184.

[13] E. Alexandri , R. Ahmed , H. Siddiqui , M. I. Choudhary , C. G. Tsiafoulis , I. P. Gerothanassis , Molecules 2017, 22, 1663. 10.3390/molecules22101663 PMC6151582 28981459

[14] Y. Feng , B. Chen , Q. Yu , L. Li , Anal. Chem. 2019, 91, 1791–1795. 30608661 10.1021/acs.analchem.8b04905 PMC6408215

[15] F. D. Gunstone , M. R. Pollard , C. M. Scrimgeour , H. S. Vedanayagam , Chem. Phys. Lipids 1977, 18, 115–129. 832335 10.1016/0009-3084(77)90031-7

[16] G. Büchi , J. D. White , G. N. Wogan , J. Am. Chem. Soc. 1965, 87, 3484–3489. 14322541 10.1021/ja01093a036

[17] S. Suzuki , T. Hosoe , K. Nozawa , T. Yaguchi , S. Udagawa , K. Kawai , J. Nat. Prod. 1999, 62, 1328–1329. 10514327 10.1021/np990146f

[18] R. C. Clark , S. Y. Lee , D. L. Boger , J. Am. Chem. Soc. 2008, 130, 12355–12369. 18712872 10.1021/ja8012819 PMC2587114

[19] C. Chepkirui , K. T. Yuyama , L. A. Wanga , C. Decock , J. C. Matasyoh , W. R. Abraham , M. Stadler , J. Nat. Prod. 2018, 81, 778–784. 29489350 10.1021/acs.jnatprod.7b00764

[20a] A. al Fahad , A. Abood , T. J. Simpson , R. J. Cox , Angew. Chem. Int. Ed. 2014, 53, 7519–7523; 10.1002/anie.201403450 24863423

[20b] W. Chen , R. Chen , Q. Liu , Y. He , K. He , X. Ding , L. Kang , X. Guo , N. Xie , Y. Zhou , Y. Lu , R. J. Cox , I. Molnar , M. Li , Y. Shao , F. Chen , Chem. Sci. 2017, 8, 4917–4925; 28959415 10.1039/c7sc00475c PMC5603960

[20c] Y. He , R. J. Cox , Chem. Sci. 2016, 7, 2119–2127. 29899939 10.1039/c5sc04027b PMC5968754

[21] A. O. Zabala , W. Xu , Y. H. Chooi , Y. Tang , Chem. Biol. 2012, 19, 1049–1059. 22921072 10.1016/j.chembiol.2012.07.004 PMC3428717

[22] P. C. Woo , C. W. Lam , E. W. Tam , C. K. Leung , S. S. Wong , S. K. Lau , K. Y. Yuen , PLoS Neglected Trop. Dis. 2012, 6, e1871. 10.1371/journal.pntd.0001871 PMC3475676 23094121

[23a] A. Abood , A. Al-Fahad , A. Scott , A. E.-D. M. S. Hosny , A. M. Hashem , A. M. A. Fattah , P. R. Race , T. J. Simpson , R. J. Cox , RSC Adv. 2015, 5, 49987–49995;

[23b] A. R. Benitez , S. Tweedy , S. A. Baker Dockrey , A. L. Lukowski , T. Wymore , D. Khare , C. L. Brooks 3rd , B. A. Palfey , J. L. Smith , A. R. H. Narayan , ACS Catal. 2019, 9, 3633–3640. 31346489 10.1021/acscatal.8b04575 PMC6658140

[24] B. Bonsch , V. Belt , C. Bartel , N. Duensing , M. Koziol , C. M. Lazarus , A. M. Bailey , T. J. Simpson , R. J. Cox , Chem. Commun. 2016, 52, 6777–6780. 10.1039/c6cc02130a 27056201

[25] note: no stereochemistry was reported for the respective compounds, thus it might differ from **8** and **10** reported herein.

[26] X. Xie , K. Watanabe , W. A. Wojcicki , C. C. Wang , Y. Tang , Chem. Biol. 2006, 13, 1161–1169. 17113998 10.1016/j.chembiol.2006.09.008

[27] K. M. Fisch , E. Skellam , D. Ivison , R. J. Cox , A. M. Bailey , C. M. Lazarus , T. J. Simpson , Chem. Commun. 2010, 46, 5331–5333. 10.1039/c0cc01162b 20552126

[28] D. N. Quang , T. Hashimoto , M. Stadler , Y. Asakawa , Tetrahedron 2005, 61, 8451–8455.

[29] Y. Tabata , S. Ikegami , T. Yaguchi , T. Sasaki , S. Hoshiko , S. Sakuma , K. Shin-Ya , H. Seto , J. Antibiot. 1999, 52, 412–414. 10.7164/antibiotics.52.412 10395277

[30] H. Ming Ge , W. Yun Zhang , G. Ding , P. Saparpakorn , Y. Chun Song , S. Hannongbua , R. X. Tan , Chem. Commun. 2008, 5978–5980. 10.1039/b812144c 19030558

[31] X. Huang , W. Zhang , S. Tang , S. Wei , X. Lu , Angew. Chem. Int. Ed. 2020, 59, 4349–4353; 10.1002/anie.201915514 31908094

[32] V. Hantke , C. Wang , E. J. Skellam , R. J. Cox , RSC Adv. 2019, 9, 35797–35802. 10.1039/c9ra07028a PMC9074748 35528102

[33a] E. Kuhnert , F. Surup , V. Wiebach , S. Bernecker , M. Stadler , Phytochemistry 2015, 117, 116–122; 26071840 10.1016/j.phytochem.2015.06.002

[33b] D. N. Quang , T. Hashimoto , M. Stadler , Y. Asakawa , J. Nat. Prod. 2004, 67, 1152–1155. 15270570 10.1021/np040063l

[34] W.-R. Abraham , C. Hesse , FEMS Microbiol. Ecol. 2003, 46, 121–128. 19719589 10.1016/S0168-6496(03)00203-4

[35] F. Surup , S. Halecker , M. Nimtz , S. Rodrigo , B. Schulz , M. Steinert , M. Stadler , Steroids 2018, 135, 92–97. 29580870 10.1016/j.steroids.2018.03.007

[36] K. Becker , A.-C. Wessel , J. J. Luangsa-Ard , M. Stadler , Biomolecules 2020, 10, 805. 10.3390/biom10050805 PMC7277860 32456162

[37] K. T. Yuyama , L. Wendt , F. Surup , R. Kretz , C. Chepkirui , K. Wittstein , C. Boonlarppradab , S. Wongkanoun , J. J. Luangsa-ard , M. Stadler , W. R. Abraham , Biomolecules 2018, 8, 129. 10.3390/biom8040129 PMC6316226 30380779

[38] C. L. M. Gilchrist , Y.-H. Chooi , bioRxiv 2020, doi: 10.1101/2020.11.08.370650.

