# Supplementary material for: Hybridorubrins A–D: Azaphilone Heterodimers from Stromata of Hypoxylon fragiforme and Insights into the Biosynthetic Machinery for Azaphilone Diversification
Source: Chemistry. 2020 Dec 10;27(4):1438–50. doi: 10.1002/chem.202003215 (PMC7898651; doi:10.1002/chem.202003215)
Supplement: Supplementary file 1 — Supplementary [file CHEM-27-1438-s001.pdf]

Chemistry–A European Journal

Supporting Information

Hybridorubrins A–D: Azaphilone Heterodimers from Stromata of *Hypoxylon fragiforme* and Insights into the Biosynthetic Machinery for Azaphilone Diversification

Kevin Becker,[a, b] Sebastian Pfütze,[a, b] Eric Kuhnert,[c, d] Russell J. Cox,[c, d] Marc Stadler,*[a, b] and Frank Surup*[a, b]

Chromatograms, DAD, and MS traces of hybridorubrins A−D and fragirubrins F−G (1−6)

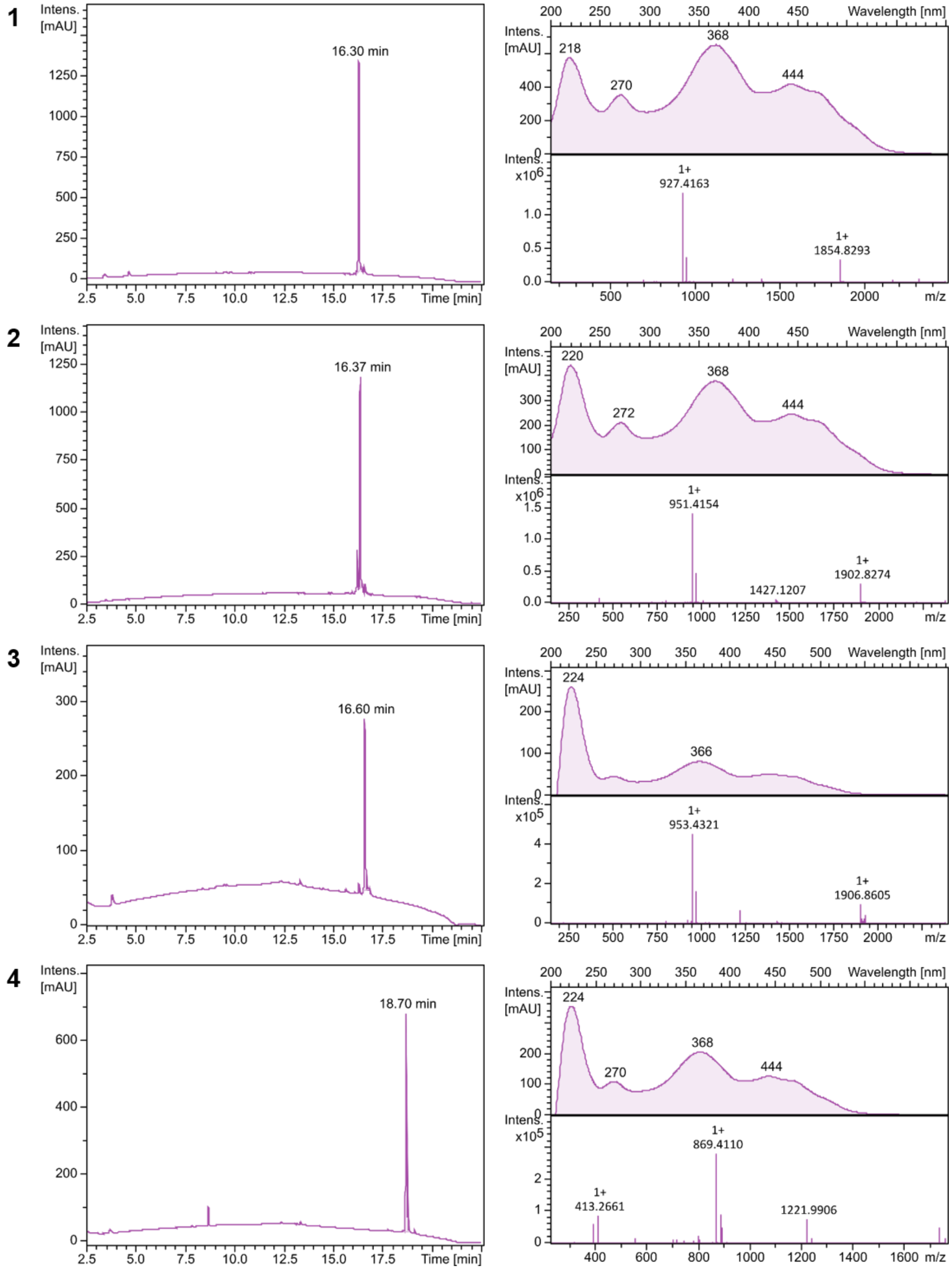

Figure S1: HPLC-UV/vis chromatograms at 210 nm, DAD and HR-ESI-MS(+) traces of hybridorubrins A−D (**1**−**4**) and fragirubrins F−G (**5**−**6**).

5

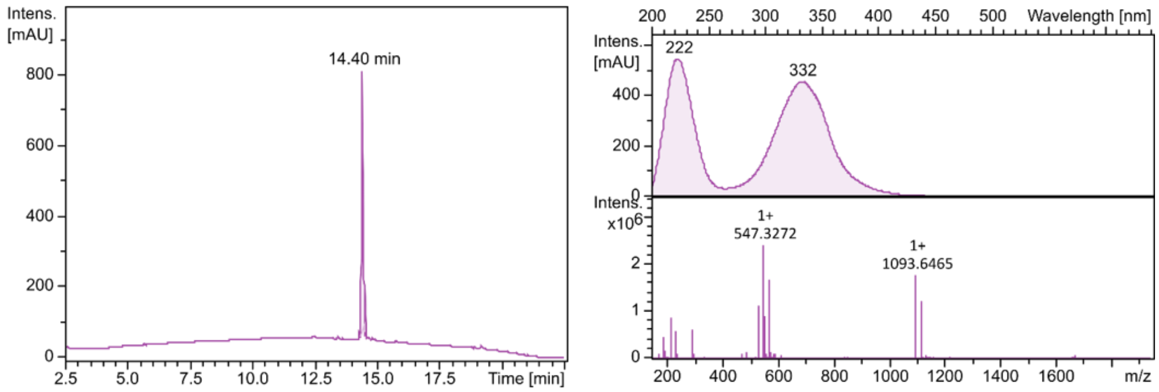

6

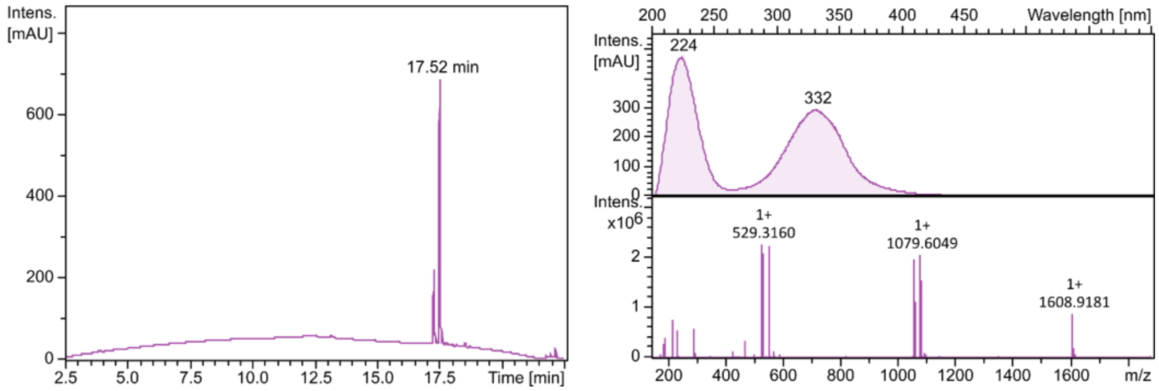

Figure S1 (continued): HPLC-UV/vis chromatograms at 210 nm, DAD and HR-ESI-MS(+) traces of hybridorubrins A−D (**1**−**4**) and fragirubrins F−G (**5**−**6**).

Protocol: minimum inhibitory concentration (MIC) assay

The assay was conducted as a minimum inhibitory concentration (MIC) assay in 96-well round-bottom microtiter plates using the parameters summarized in Table S1 and as described previously.[S1]

Stocks of the test organisms were generated by growing the organisms overnight in 50 mL shaking flasks filled with 25 mL of the growth medium at 140 rpm (for media and temperatures see Table S1). If the organisms were well grown the next day, which was checked by occurrence of an optical density (OD)>30 of the suspension ($OD_{600\ nm}$ for bacteria, $OD_{548\ nm}$ for fungi and *M. smegmatis*), aliquots of these were stored in 1.5 mL reaction tubes in a freezer at −80 °C for up to 12 months. Upon use, aliquots were unthawed and the OD of the suspension measured and adjusted by diluting with the respective growth medium. $OD_{600\ nm}$ was adjusted to 0.01 and $OD_{548\ nm}$ to 0.1.

Subsequently, 150 µL of the adjusted suspensions were added to all wells of a 96-well microtiter plate (one test organism per plate). In row A, additional 130 µL of suspensions plus 20 µL of the test compounds (1 mg×mL^{-1}) and the controls (one compound/column) were added. MeOH was used as negative controls, while different positive controls (references) were used for the test organisms (see Table S1). Then, starting from row A, 150 µL of the suspension were transferred to the next row, the contents thoroughly mixed, and 150 µL transferred to the following row. The remaining 150 µL after row H were discarded. This resulted in a serial dilution of the test compounds, ranging from 66.7 µg×mL^{-1} in row A to 0.52 µg×mL^{-1} in row H.

The microtiter plates were then incubated overnight on a microplate shaker at 800 rpm at 30 or 37 °C (see Table S1) and were visually evaluated the next day. The MIC is defined as the lowest concentration were no growth of the test organism was observed. A lower MIC thus corresponds to a higher antimicrobial activity of the test compound. Results can be found in Table S3.

[S1] K. Becker, A.-C. Wessel, J.J. Luangsa-ard, M. Stadler, *Biomolecules.* **2020**, *10*(5).

Table S1: MIC assay experiment parameters

test organism	strain No.	growth medium	incubation temp. [°C]	positive control
Bacillus subtilis	DSM 10	MHB [1]	30	oxytetracycline (1.0 mg×mL^{-1})
Staphylococcus aureus	DSM 346	MHB	30	oxytetracycline (0.1 mg×mL^{-1})
Micrococcus luteus	DSM 1790	MHB	30	oxytetracycline (0.1 mg×mL^{-1})
Chromobacterium violaceum	DSM 30191	MHB	30	oxytetracycline (0.1 mg×mL^{-1})
Escherichia coli	DSM 1116	MHB	37	oxytetracycline (0.1 mg×mL^{-1})
Pseudomonas aeruginosa	PA14	MHB	37	gentamicin (0.1 mg×mL^{-1})
Mycolicibacterium smegmatis	ATCC 700084	7H9+ADC [2]	37	kanamycin (0.1 mg×mL^{-1})
Candida albicans	DSM 1665	MYC [3]	30	nystatin (1.0 mg×mL^{-1})
Schizosaccharomyces pombe	DSM 70572	MYC	30	nystatin (1.0 mg×mL^{-1})
Mucor hiemalis	DSM 2656	MYC	30	nystatin (1.0 mg×mL^{-1})
Pichia anomala	DSM 6766	MYC	30	nystatin (1.0 mg×mL^{-1})
Rhodotorula glutinis	DSM 10134	MYC	30	nystatin (1.0 mg×mL^{-1})

[1] MHB: Müller-Hinton Broth (SN X927.1, Carl Roth GmbH, Karlsruhe, Germany)
[2] 7H9+ADC: Middlebrook 7H9 Broth Base + Middlebrook ADC Growth Supplement (SN M0678+M0553, Merck, Darmstadt, Germany)
[3] MYC: 1% w/v, bacto peptone, 1% w/v yeast extract, 2% w/v glycerol, pH 6.3

Protocol: cytotoxicity assay

The assay was conducted in 96-well flat-bottom microtiter plates using the parameters summarised in Table S2 and as already described.[S2]

Cell lines L929 and KB 3.1 were incubated at 37 °C under 10% CO_2 in Gibco™ DMEM medium (Thermo Fisher Scientific, Waltham, MA/USA) supplemented with 10% FBS. A microtiter plate was filled with 120 µL of this suspension (50,000×mL^{-1}) in each well.

Seperately, another microtiter plate was filled with 100 µL of growth medium in each well. Then, 50 µL of the test compound solutions (1 mg×mL^{-1}) were given to wells of the first column in two replicates (one compound per row). Cells without additives, MeOH, and MeOH:DMSO 9+1 were used as negative controls. Starting from the first column, 50 µL of the solutions were gradually transferred to the next column, the contents thoroughly mixed, and 50 µL transferred to the following column. This created a serial dilution of the test compounds ranging from 333 µg×mL^{-1} to 1.9×10^{-3} µg×mL^{-1}. The remaining 50 µL after column twelve were discarded. From this microtiter plate, 60 µL of the solutions from 111 µg×mL^{-1} to 1.9×10^{-3} µg×mL^{-1} were given to the first plate containing 120 µL of the cell suspensions (*i.e.* the highest concentration

[S2] B. Sandargo, M. Michehl, D. Praditya, E. Steinmann, M. Stadler, F. Surup, *Org. Lett.* **2019,** *21*, 3286–3289.

333 µg×mL^{-1} was not used). This resulted in final compound concentrations ranging from 37 µg×mL^{-1} to 0.6×10^{-3} µg×mL^{-1}.

After 5 days of incubation under the aforementioned incubation conditions, the half maximum inhibitory concentrations (IC_{50}) were determined using a colorimetric tetrazolium dye MTT assay.[S3] For this, 20 µL of a 5 mg×mL^{-1} solution of 3-(4,5-dimethyl-2-thiazolyl)-2,5-diphenyl-2*H*-tetrazolium bromide (MTT) were added to each well and incubated for two hours at 37 °C. Then, the microtiter plate was centrifuged (3,000 rpm, 5 min) and the supernatant removed by holding the plate upside-down and gentle shaking. Afterwards, the wells were washed using 100 µL of phosphate buffered saline (PBS). The plate was again centrifuged and the supernatant removed as described before. Then, 100 µL of an isopropanol:HCl solution (1L isopropanol + 4 mL HCl 37% w/v) were added to the wells. After incubating for 10 min at ambient temperature, the absorption of the wells at 595 nm was measured with an Infinite® 200 Pro microplate reader (TECAN, Männedorf, Schweiz).

The absorption values of the cells without additives were averaged and set to 100% cell viability. Then, the means of absorption of the two compound replicates were set in relation to the blank media. These percentage values were plotted against the concentration range (37 µg/mL to 0.6×10^{-3} µg×mL^{-1}). The IC_{50} value was read from the plot (in µg×mL^{-1}) and the units conversed to µM. Results can be found in Table S3.

Table S2: Cytotoxicity assay experiment parameters

cell line	type	No.	growth medium
L929	mouse fibroblasts	ACC 2	DMEM [1]<br>+ 10% FBS [2]
KB 3.1	human endocervical adenocarcinoma (AC)	ACC 158	DMEM<br>+ 10% FBS

[1] DMEM: Dulbecco's Modified Eagle Medium (SN 61965026, Thermo Fisher Scientific, Waltham, MA/USA)
[2] FBS: Fetal Bovine Serum (SN 10500064, Thermo Fisher Scientific)

[S3] T. Mosmann, *J. Immunol. Methods* **1983**, *65*, 55–63.

Results: minimum inhibitory concentration (MIC) assay and cytotoxicity assay

Table S3: Minimum inhibitory concentrations (MIC) (top panel) and cytotoxicity values (IC_{50}) (bottom panel) of **1−2**, **4−10**, **12−18**. n.i.: no inhibition

test organism / cell line	strain number	MIC [µg×mL^{-1}] / IC_{50} [µM]															
		**1**	**2**	**4**	**5**	**6**	**7**	**8**	**9**	**10**	**12**	**13**	**14**	**15**	**16**	**17**	**18**
organism																	
Bacillus subtilis	DSM 10	>66.7	>66.7	>66.7	>66.7	>66.7	>66.7	>66.7	>66.7	>66.7	>66.7	>66.7	>66.7	>66.7	>66.7	>66.7	>66.7
Staphylococcus aureus	DSM 346	>66.7	>66.7	>66.7	>66.7	>66.7	>66.7	>66.7	>66.7	>66.7	>66.7	>66.7	>66.7	>66.7	>66.7	>66.7	>66.7
Micrococcus luteus	DSM 1790	>66.7	>66.7	>66.7	>66.7	>66.7	>66.7	>66.7	>66.7	>66.7	>66.7	>66.7	>66.7	>66.7	>66.7	>66.7	>66.7
Chromobacterium violaceum	DSM 30191	>66.7	>66.7	>66.7	>66.7	>66.7	>66.7	>66.7	>66.7	>66.7	>66.7	>66.7	>66.7	>66.7	>66.7	>66.7	>66.7
Escherichia coli	DSM 1116	>66.7	>66.7	>66.7	>66.7	>66.7	>66.7	>66.7	>66.7	>66.7	>66.7	>66.7	>66.7	>66.7	>66.7	>66.7	>66.7
Pseudomonas aeruginosa	PA14	>66.7	>66.7	>66.7	>66.7	>66.7	>66.7	>66.7	>66.7	>66.7	>66.7	>66.7	>66.7	>66.7	>66.7	>66.7	>66.7
Mycolicibacterium smegmatis	ATCC 700084	>66.7	>66.7	>66.7	>66.7	>66.7	>66.7	>66.7	>66.7	>66.7	>66.7	>66.7	>66.7	>66.7	>66.7	>66.7	>66.7
Candida albicans	DSM 1665	>66.7	>66.7	>66.7	>66.7	>66.7	>66.7	>66.7	>66.7	>66.7	>66.7	>66.7	>66.7	>66.7	>66.7	>66.7	>66.7
Schizosaccharomyces pombe	DSM70572	>66.7	>66.7	>66.7	>66.7	>66.7	>66.7	>66.7	>66.7	>66.7	>66.7	>66.7	>66.7	>66.7	>66.7	>66.7	>66.7
Mucor hiemalis	DSM 2656	>66.7	>66.7	>66.7	>66.7	>66.7	>66.7	>66.7	>66.7	>66.7	>66.7	>66.7	>66.7	>66.7	>66.7	>66.7	>66.7
Pichia anomala	DSM 6766	>66.7	>66.7	>66.7	>66.7	>66.7	>66.7	>66.7	>66.7	>66.7	>66.7	>66.7	>66.7	>66.7	>66.7	>66.7	>66.7
Rhodotorula glutinis	DSM 10134	>66.7	>66.7	>66.7	>66.7	>66.7	>66.7	>66.7	>66.7	>66.7	>66.7	>66.7	>66.7	>66.7	>66.7	>66.7	>66.7
cell line																	
mouse fibroblasts	L929	n.i.	n.i.	n.i.	n.i.	n.i.	n.i.	n.i.	n.i.	n.i.	n.i.	n.i.	n.i.	n.i.	n.i.	n.i.	n.i.
human endocervical adenocarcinoma	KB 3.1	n.i.	n.i.	n.i.	n.i.	n.i.	n.i.	n.i.	n.i.	n.i.	n.i.	n.i.	n.i.	n.i.	n.i.	n.i.	n.i.

Protocol: biofilm formation inhibition assay

The assay was conducted in duplicates in untreated 96-well flat-bottom microtiter plates as already described.[S4]

A culture of *Staphylococcus aureus* (DSM 1104) was incubated overnight in CASO medium (casein-peptone soymeal-peptone broth; supplemented with 4% glucose) at 37 °C. The next day, the suspension was diluted with CASO medium to reach a turbidity of 0.5 McFarland using a NanoDrop™ 2000 spectrophotometer (Thermo Fisher Scientific, Waltham, MA/USA) at 600 nm. A 96-well flat bottom microtiter plate (SN 351172, Corning, Corning, NY/USA) was filled with 150 µL of the cell suspension to each well. Then, 15 µL of the test compounds (5 mg×mL^{-1} in MeOH) as well as further 135 µL of CASO medium were added to wells B2–B6 in two replicates (one compound per row). MeOH and microporenic acid (5 mg×mL^{-1} in MeOH) were used as negative and positive controls, respectively.

Starting from the first column, 150 µL of the solutions were gradually transferred to the next column, the contents thoroughly mixed, and 150 µL transferred to the following column, until column 8 was reached. The remaining 150 µL after column 8 were discarded. This created a serial dilution of the test compounds ranging from 250 µg×mL^{-1} to 3.9 µg×mL^{-1}. MeOH and microporenic acid were only diluted up to column 7, resulting in the lowest concentration to be 7.8 µg×mL^{-1}. The microtiter plates were then covered with adhesive porous paper (Kisker Biotech, Steinfurt, Germany) and incubated for 24 h at 37 °C.

On the next day, the supernatant was removed by holding the plate upside-down and gentle shaking over tissue paper. Then, 150 µL of crystal violet solution (0.1% w/v in H_2O) were added and the microtiter plate incubated for 15 min at ambient temperature. The crystal violet solution was discarded as described before. Afterwards, 150 µL of phosphate buffered saline (PBS) were added, mixed, and discarded again. This washing procedure was conducted three times in total. At last, the PBS was removed and 125 µL of 30% acetic acid added to each well and incubated at ambient temperature for 15 min. Then, the solutions within the 96 wells of each plate were transferred to a new microtiter plate and the absorbance at 550 nm measured using a Synergy 2 microplate reader (BioTek, Winooski, VT/USA).

The absorption values of the cells containing the MeOH serial dilutions were averaged and set to 100% cell viability. Then, the means of absorption of the four compound replicates were set in relation to the 100% cell viability for each concentration step. The growth inhibition was defined as 100%−cell viability%, *e.g.* 19% cell viability resulted in 81% inhibition. For evaluation, calculated inhibition values were categorized as: +++: if inhibition ≥70% was measured for any concentration step; ++, inhibition ≥40 and <70%; +: inhibition ≥20 and <40%; −: inhibition <20%.

[S4] Yuyama, K.T. *et al.*, *Biomolecules* **2018**, *8*.

Correlation of the biosynthetic hypothesis with detectable compounds

To identify whether predicted intermediates of the azaphilone biosynthetic pathway can be found in the stromata by UV and mass spectra analysis new stromatal crude extracts were obtained. Therefore, fresh specimens of *H. fragiforme* (EKEL19001) were collected from dead trunks of *Fagus sylvatica* in the Eilenriede forest (Hannover, Germany) at the 16th of July 2019. Approximately 0.5 cm² of the stromata surface were removed from the fungus and extracted with 100 µL acetone for 30 min. The extracts were filtered and analysed by analytical HPLC using previously described methods.[S5]

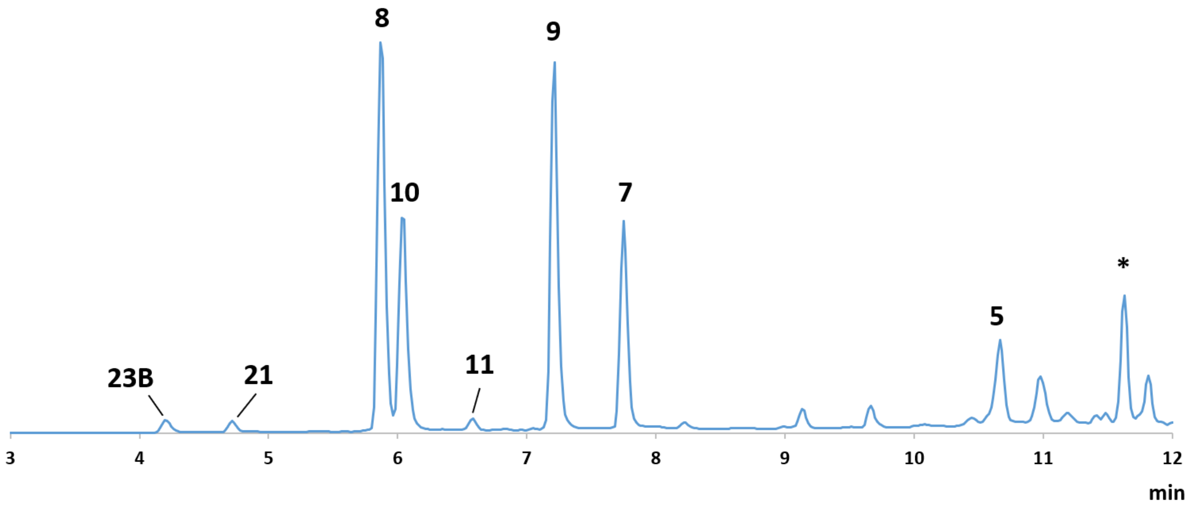

Figure S2: DAD spectrum (200−600 nm) of the stromatal acetone extract from *H. fragiforme* EKEL19001. Numbers refer to structures depicted in the main article.

[S5] K.E. Lebe, R.J. Cox, *Chem. Sci.* **2019**, *10*, 1227−1231

Table S4: DAD absorption spectra for compounds detected in the stromatal crude extracts of *H. fragiforme* EKEL19001. Compound **21** is only hypothesized based on the biosynthetic knowledge.

Compound **DAD spectrum**

23B

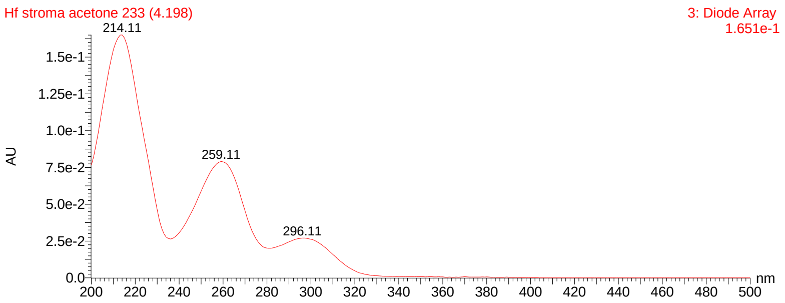

[21]

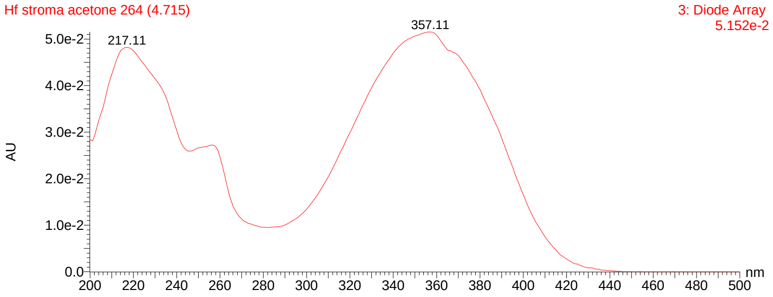

8

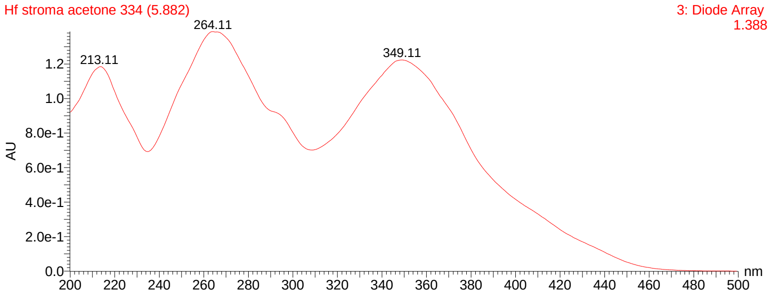

10

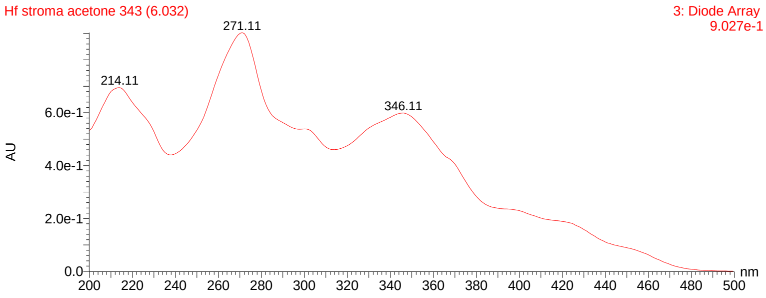

11

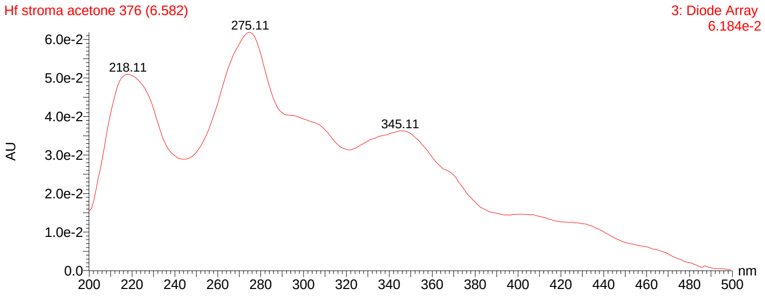

Table S4 (continued): DAD absorption spectra for compounds detected in the stromatal crude extracts of *H. fragiforme* EKEL19001. Compound 21 is only hypothesized based on the biosynthetic knowledge.

**Compound**	**DAD spectrum**
**9**	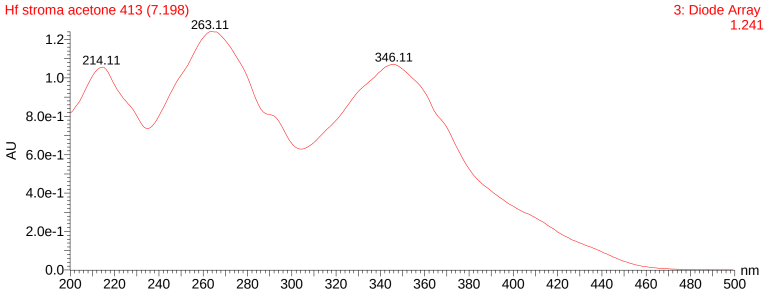
**7**	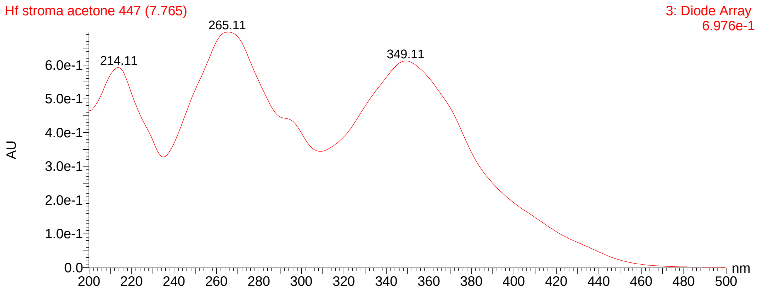
**5**	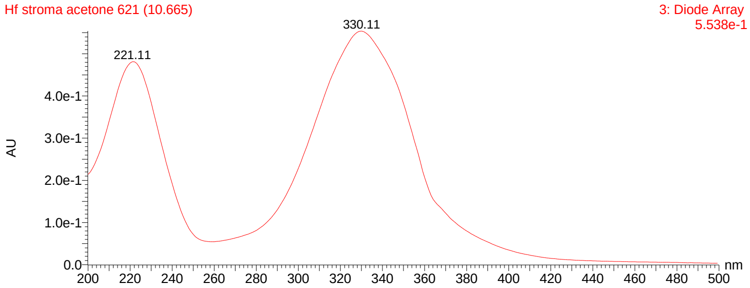
*****	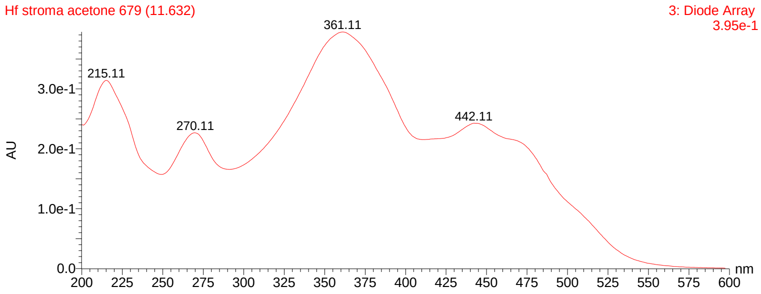

Table S5: Mass spectra from the positive and negative ionization mode and retention time (t_R) for compounds detected in the stromatal crude extracts of *H. fragiforme* EKEL19001. Compounds in brackets are predicted based on the biosynthetic hypothesis and lack structural confirmation, but respective appropriate masses were found by extracted ion chromatograms.

Compound/t_R	MS spectrum ESI (+)	MS spectrum ESI (−)
**23B**<br>4.0 min	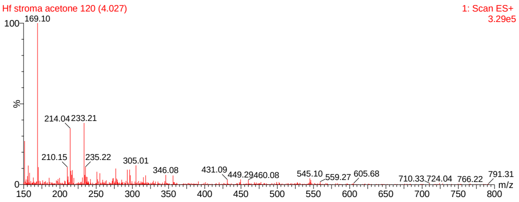	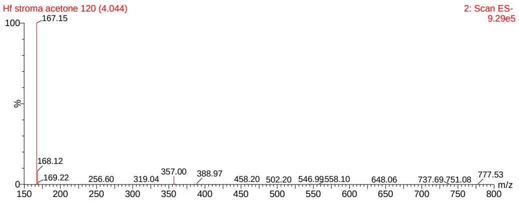
**[21]**<br>4.5 min	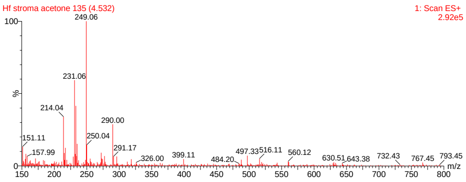	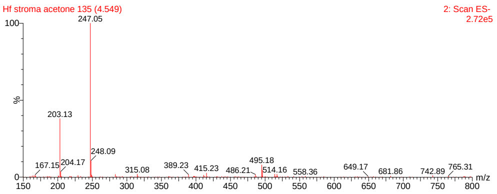
**8**<br>5.7 min	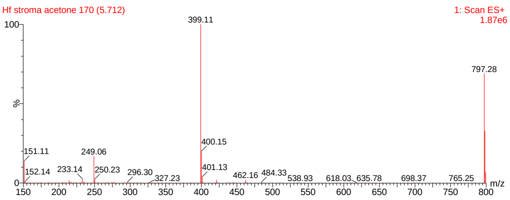	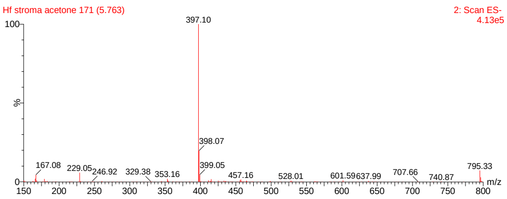
**10**<br>5.9 min	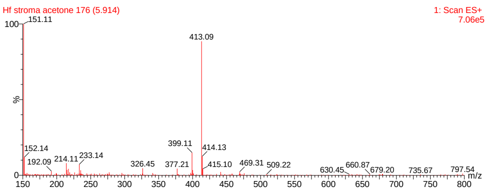	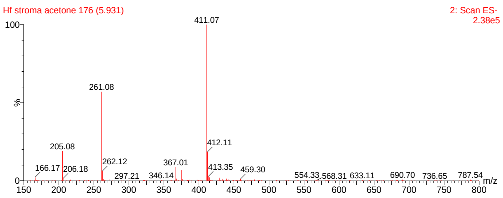
**11**<br>6.4 min	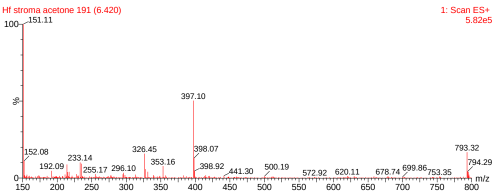	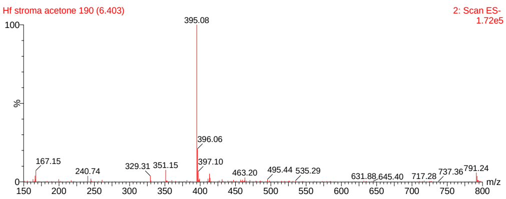
**9**<br>7.0 min	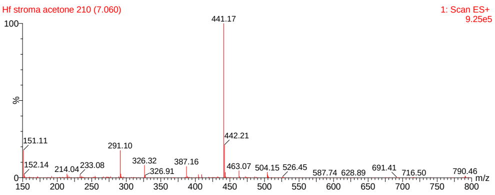	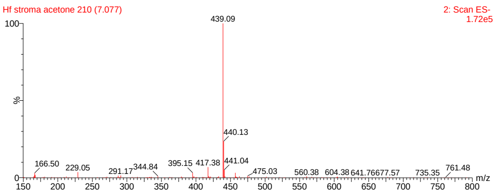
**7**<br>7.6 min	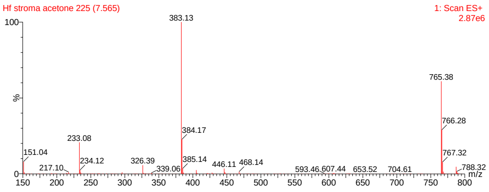	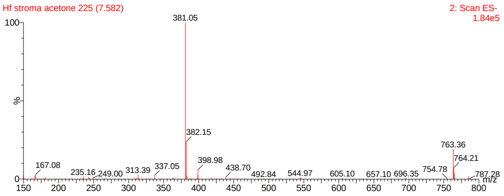

Table S5 (continued): Mass spectra from the positive and negative ionization mode and retention time (t_R) for compounds detected in the stromatal crude extracts of *H. fragiforme* EKEL19001. Compounds in brackets are predicted based on the biosynthetic hypothesis and lack structural confirmation, but respective appropriate masses were found by extracted ion chromatograms.

**Compound/t_R**	**MS spectrum ESI (+)**	**MS spectrum ESI (−)**
**5** 10.5 min	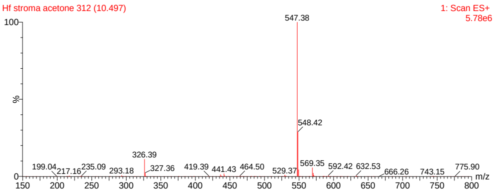	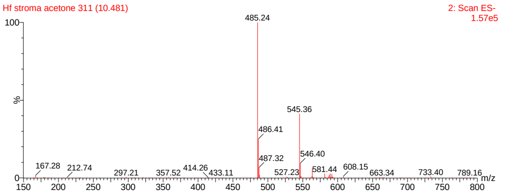
***** 11.4 min	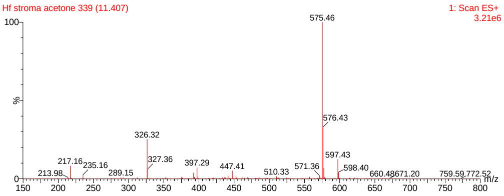	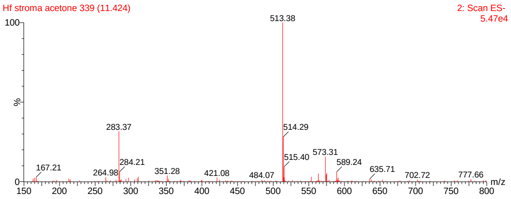
**[24]** 5.5 min	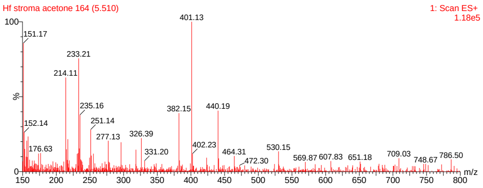	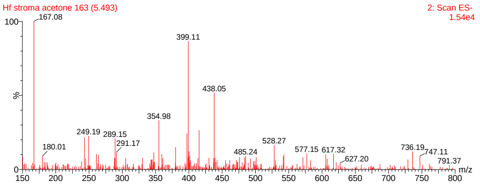
**[25]** 6.9 min	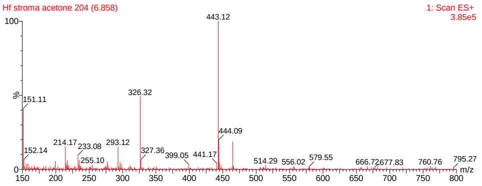	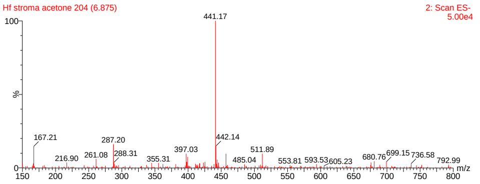

Structures of known bisazaphilone compounds

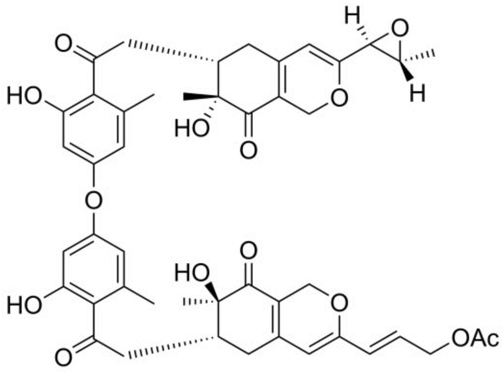

26

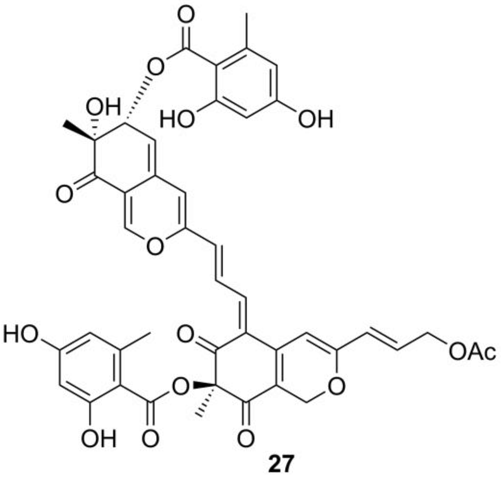

27

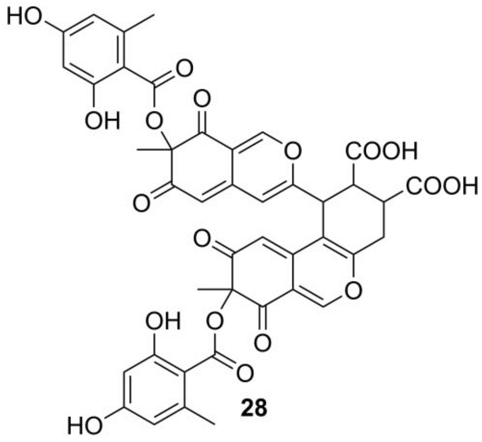

28

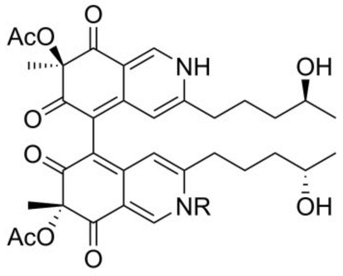

29

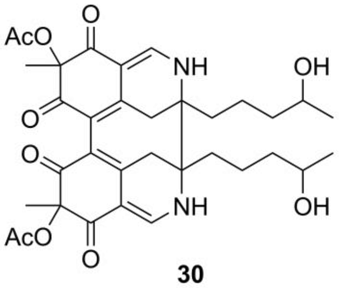

30

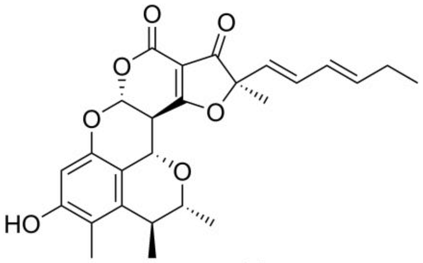

31

Figure S3: structures of representatives of known bisazaphilones (**26**−**30**) and an azaphilone of mixed biosynthetic origin (**31**). **26**: entonaemin C from *Entonaema liquescens*; **27**: rutilin A from *Hypoxylon rutilum*; **28**: diazaphilonic acid from *Talaromyces flavus*; **29**: chaetoglobin A from *Chaetomium globosum*; **30**: chaetofusin A from *Chaetomium fusiforme*; **31**: citrifuran A from *Aspergillus* sp.

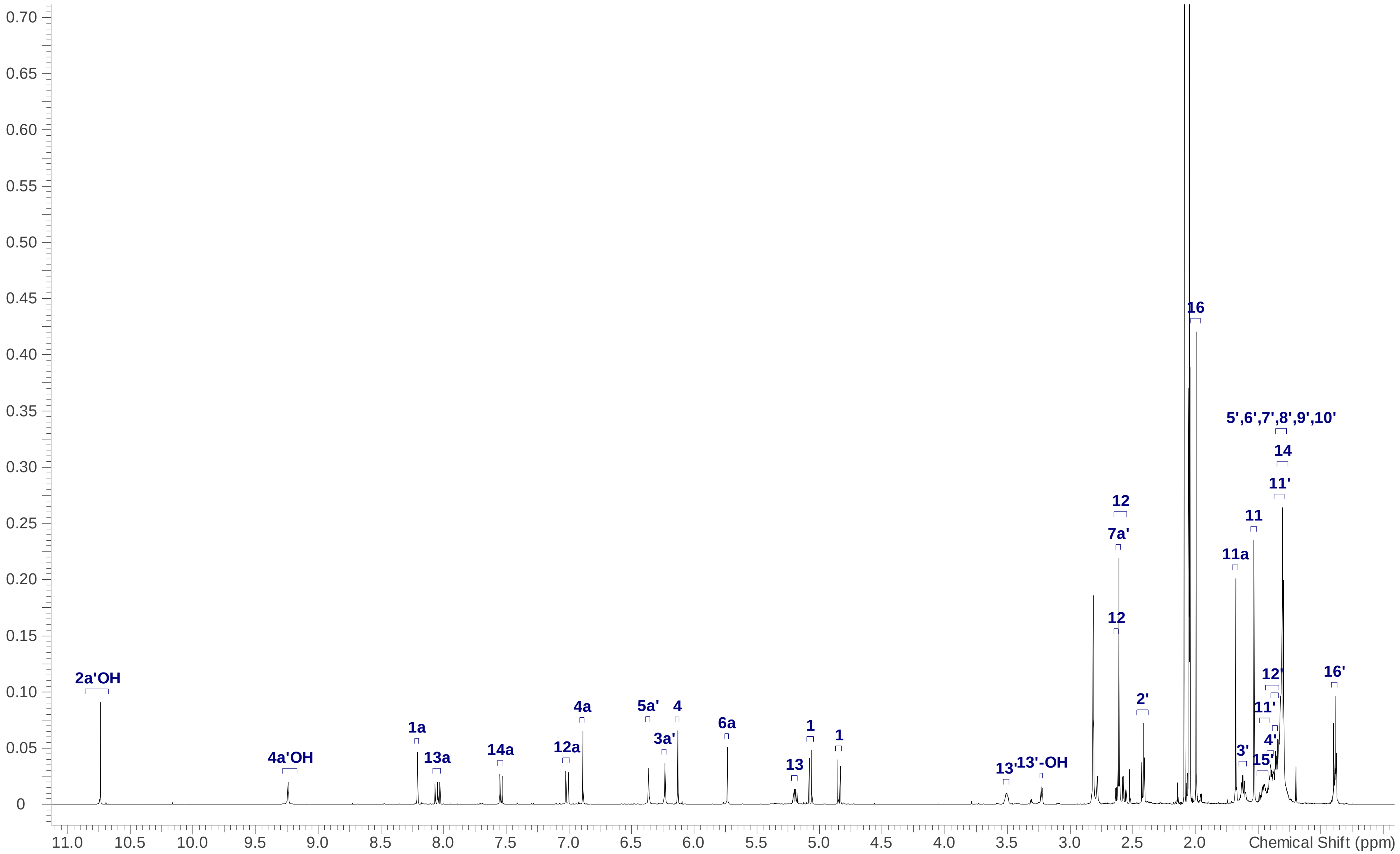

Figure S4: ^{1}H NMR spectrum (700 MHz, acetone-d_6) of hybridorubrin A (**1**).

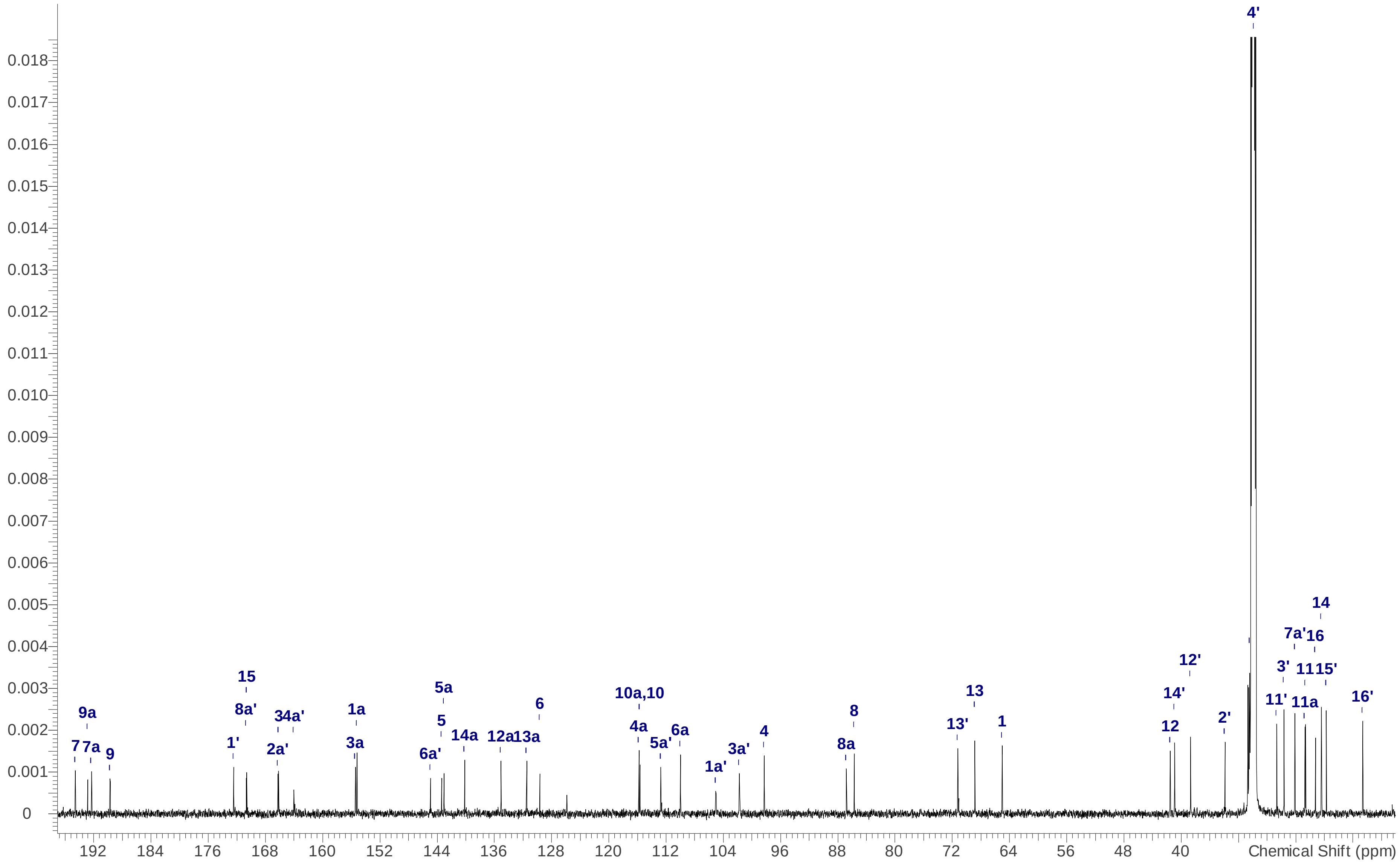

Figure S5: ^{13}C NMR spectrum (175 MHz, acetone-*d6*) of hybridorubrin A (**1**).

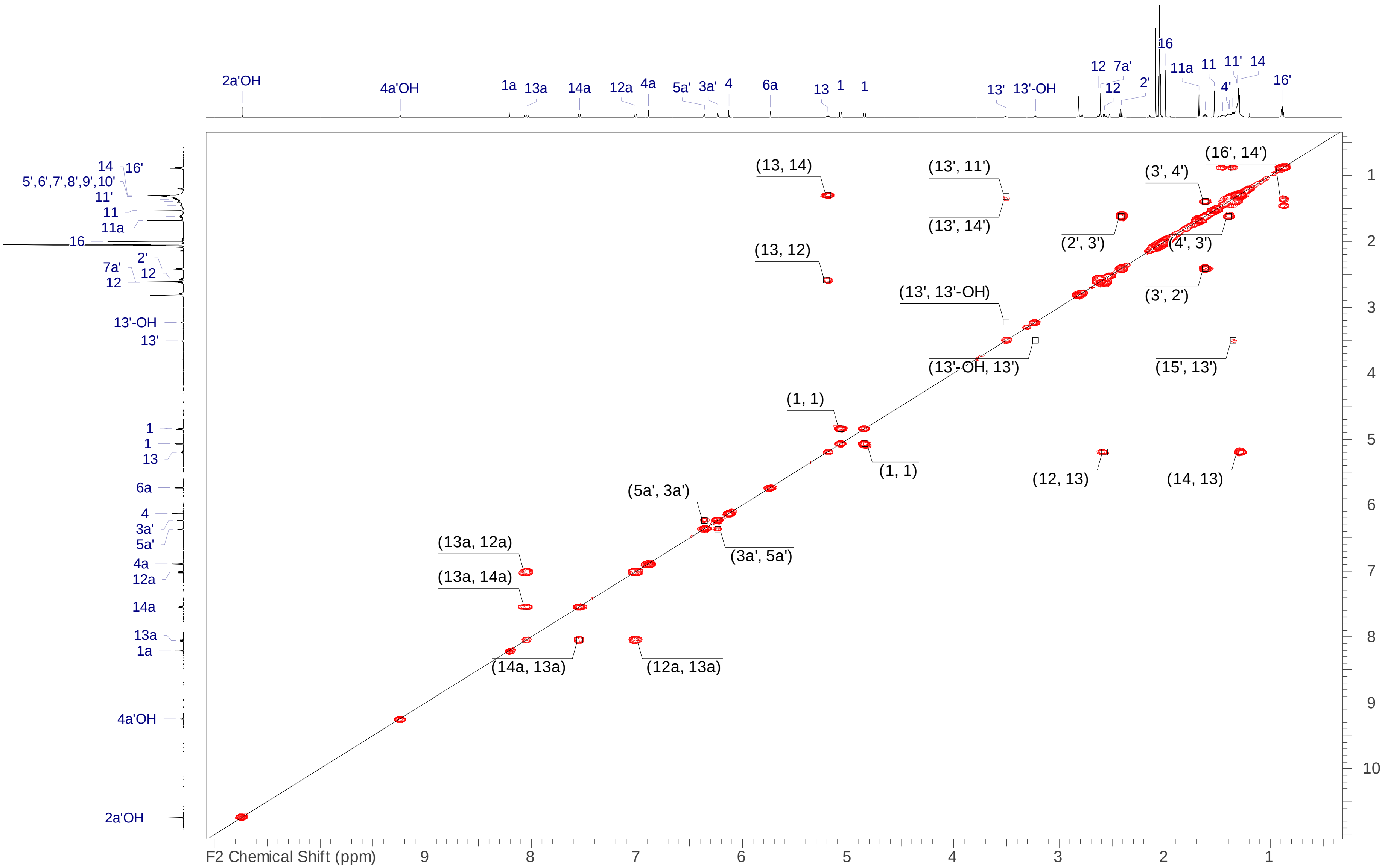

Figure S6: $^{1}H/^{1}H$ COSY spectrum (700 MHz, acetone-*d*6) of hybridorubrin A (**1**).

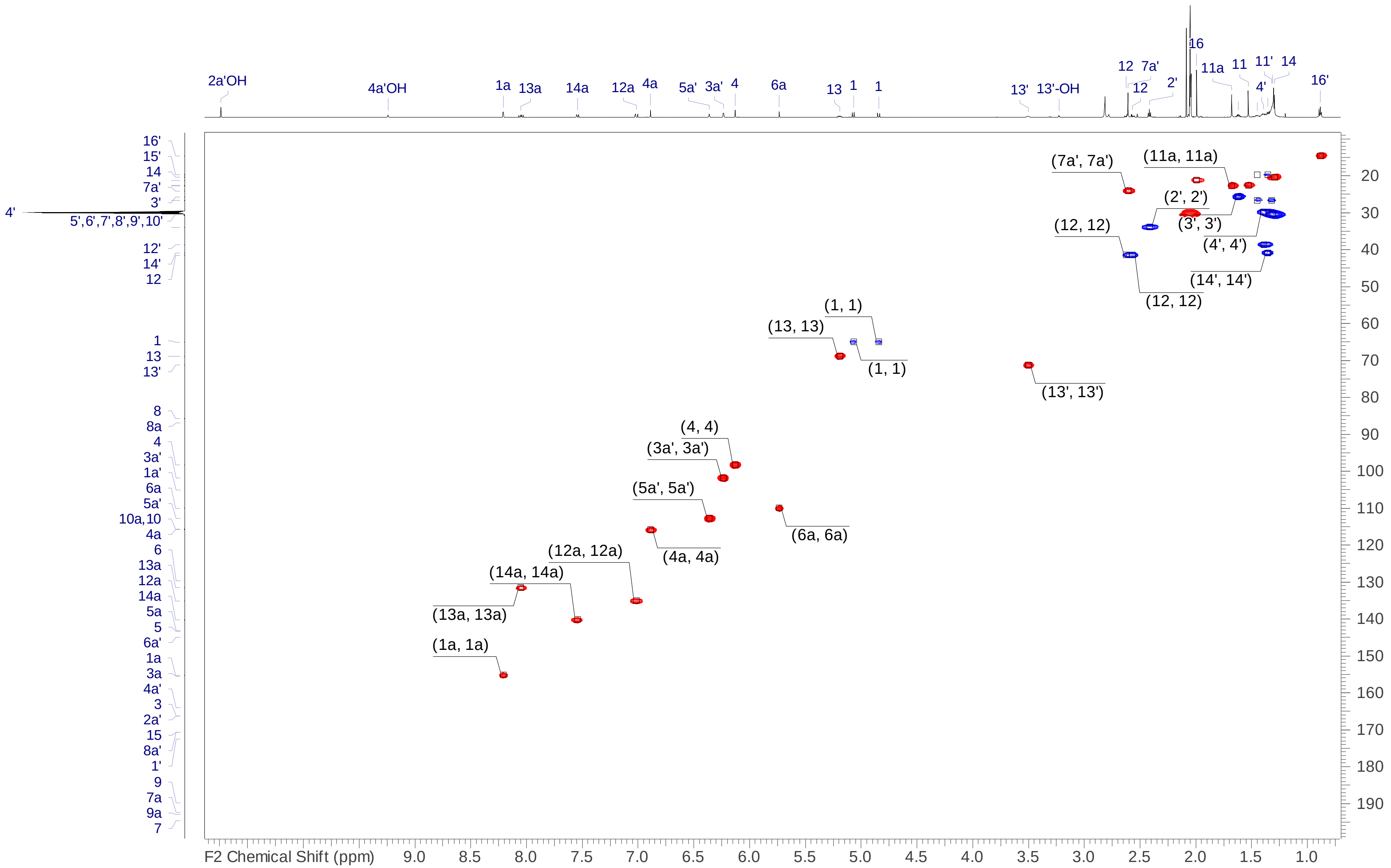

Figure S7: $^{1}H/^{13}C$ HSQC spectrum (700 MHz, acetone-*d6*) of hybridorubrin A (**1**).

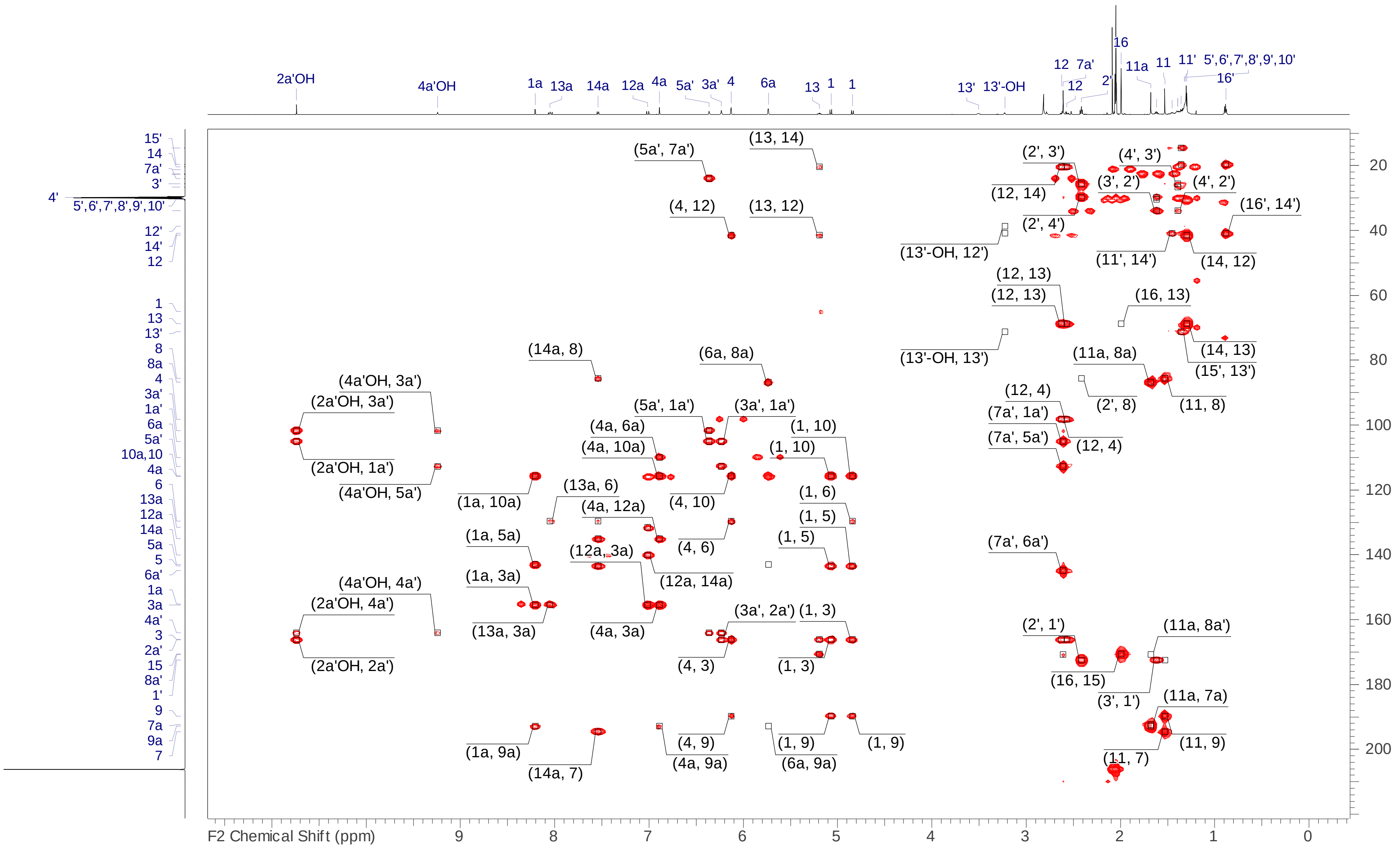

Figure S8: $^{1}H/^{13}C$ HMBC spectrum (700 MHz, acetone-*d6*) of hybridorubrin A (**1**).

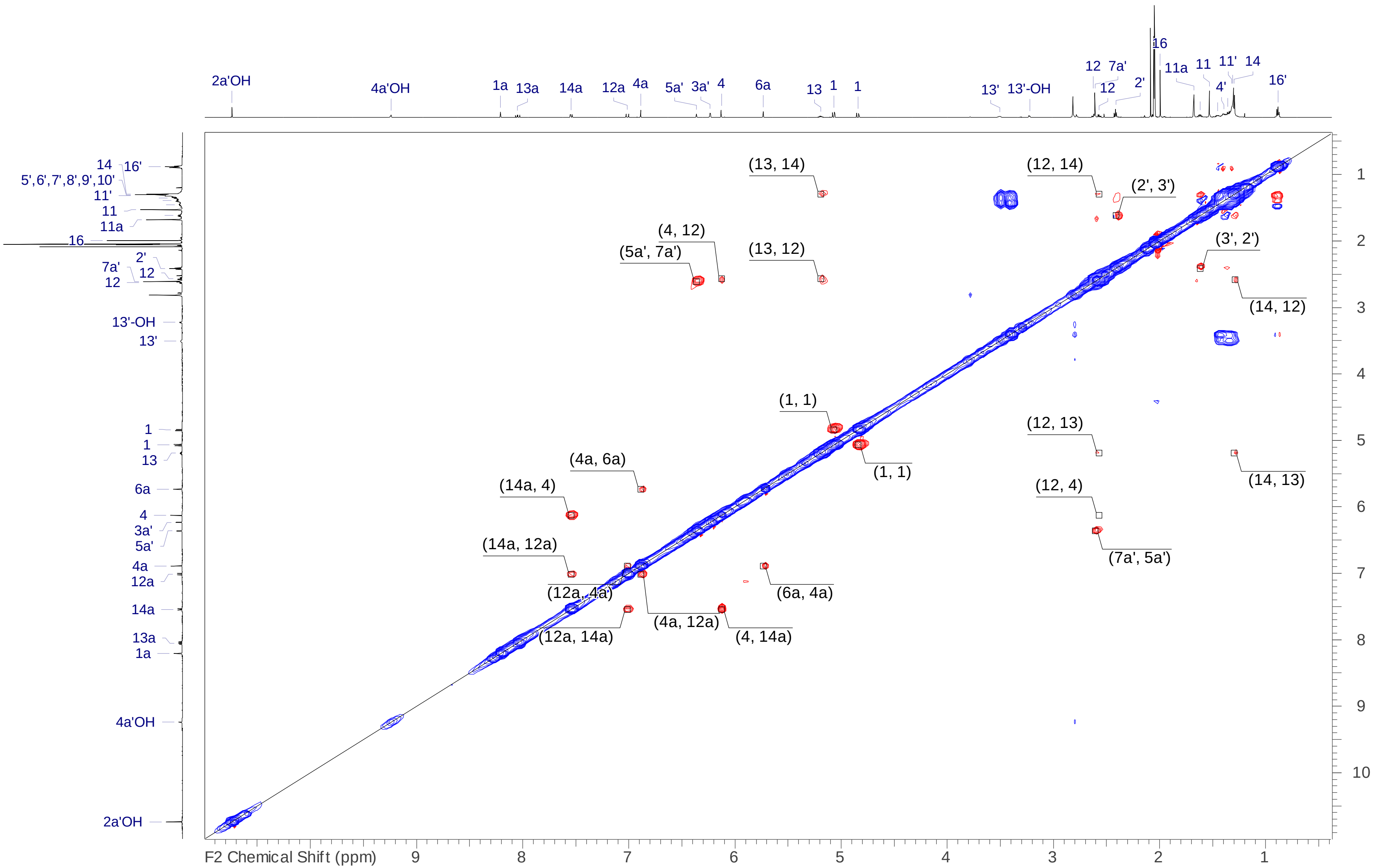

Figure S9: ^{1}H/^{1}H ROESY spectrum (700 MHz, acetone-*d*6) of hybridorubrin A (**1**).

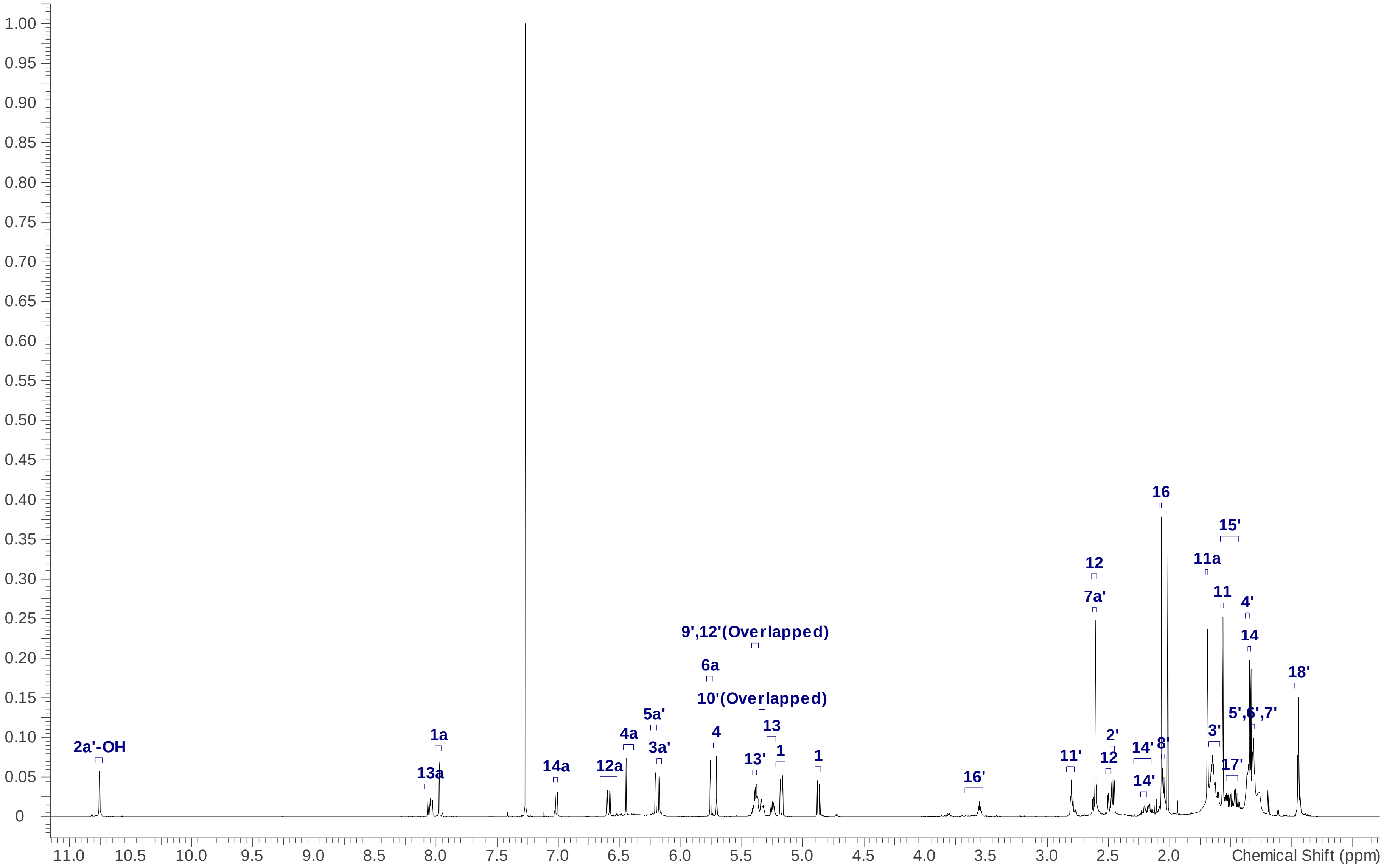

Figure S10: ^{1}H NMR spectrum (500 MHz, $CDCl_3$) of hybridorubrin B (**2**).

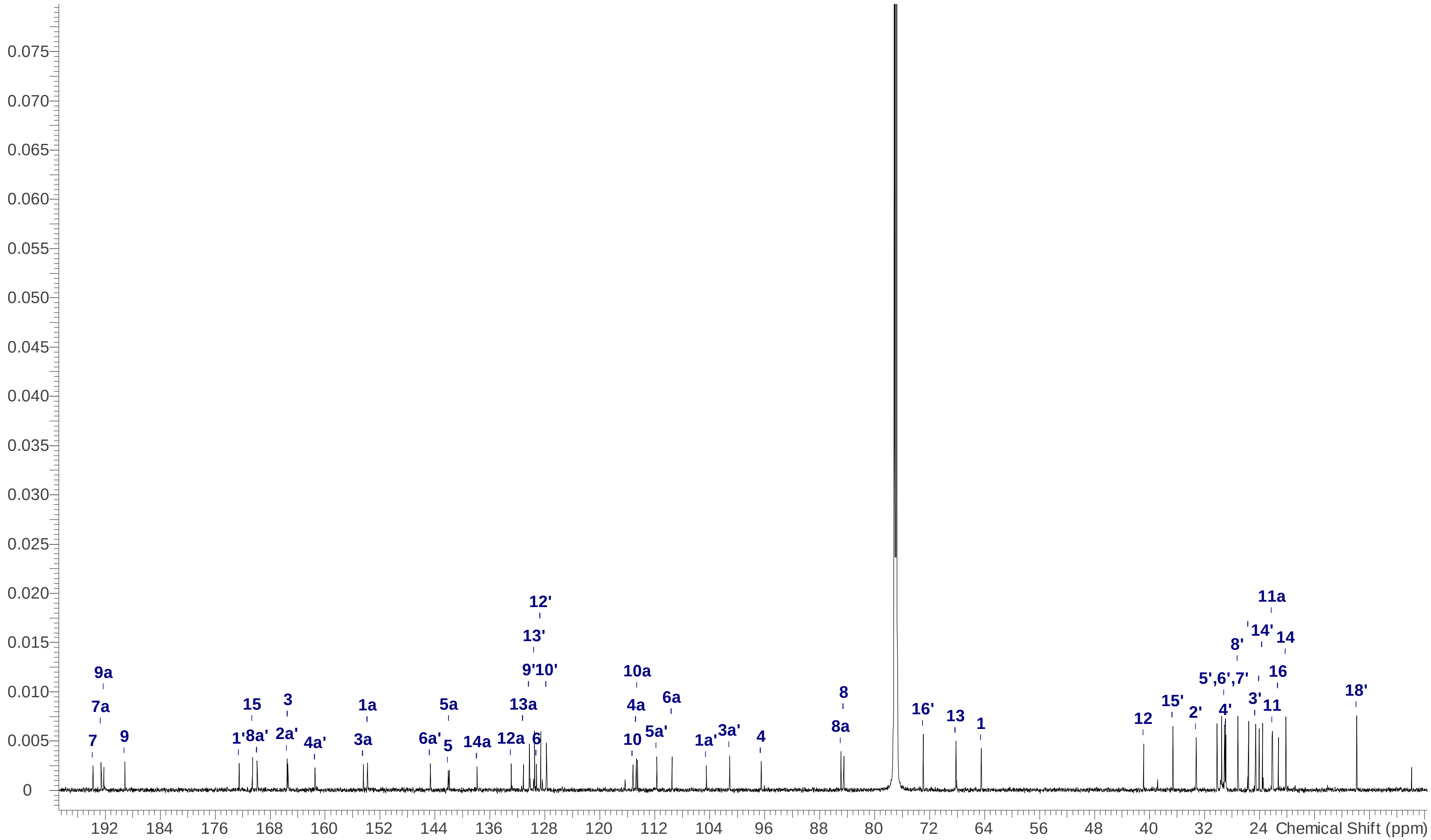

Figure S11: ^{13}C NMR spectrum (125 MHz, $CDCl_3$) of hybridorubrin B (**2**).

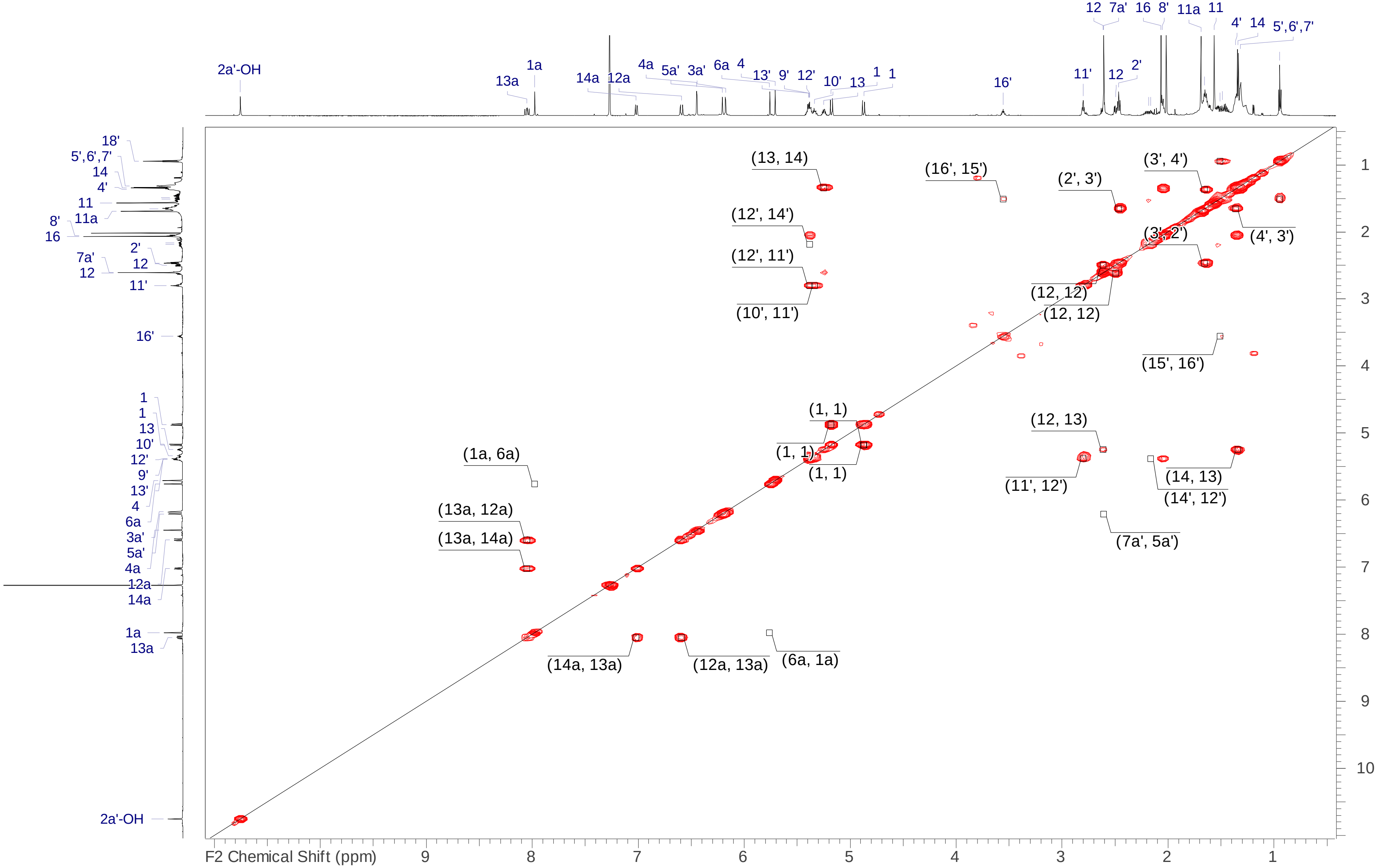

Figure S12: $^{1}H/^{1}H$ COSY spectrum (500 MHz, $CDCl_3$) of hybridorubrin B (**2**).

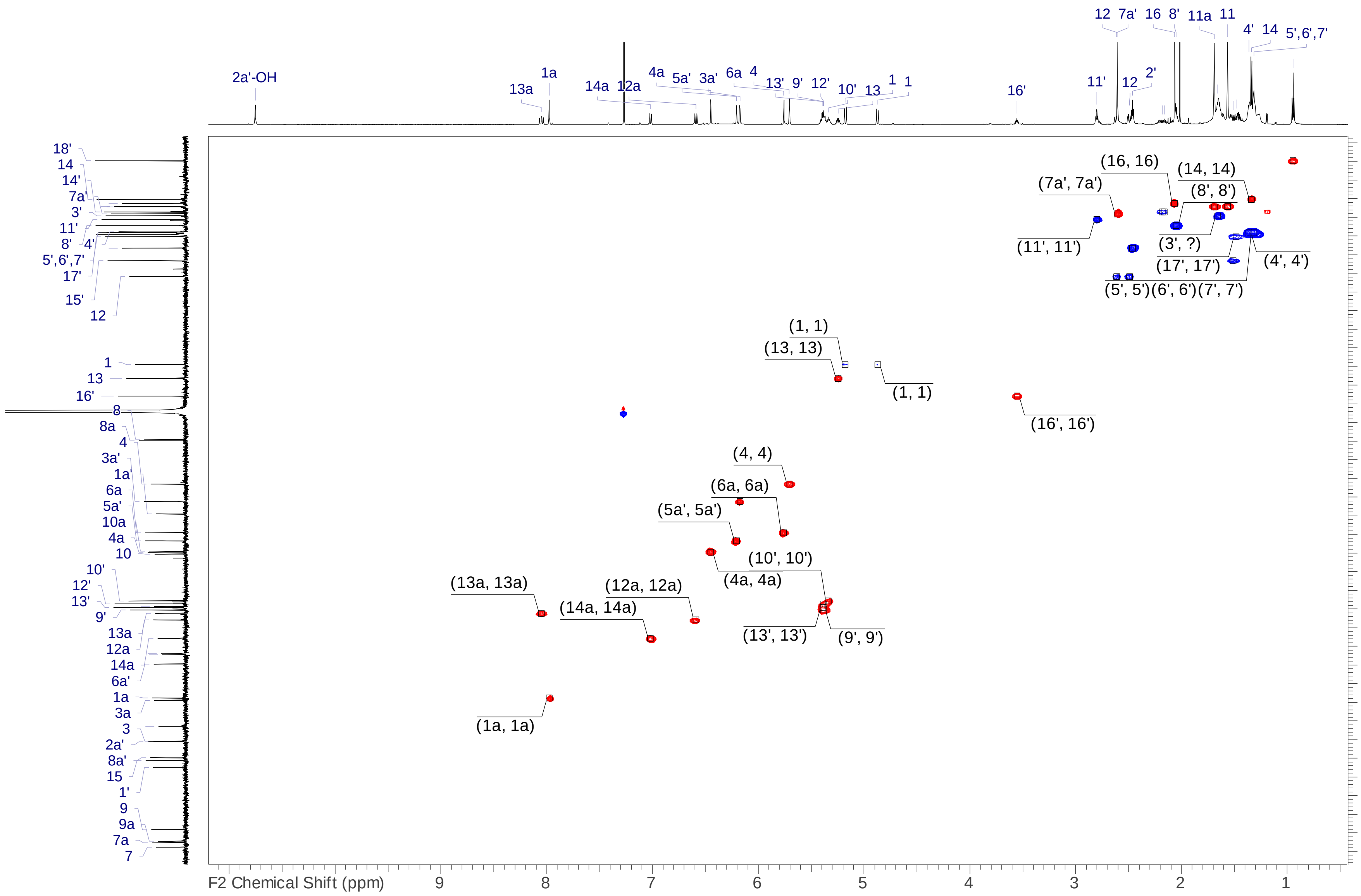

Figure S13: $^{1}H/^{13}C$ HSQC spectrum (500 MHz, $CDCl_3$) of hybridorubrin B (**2**).

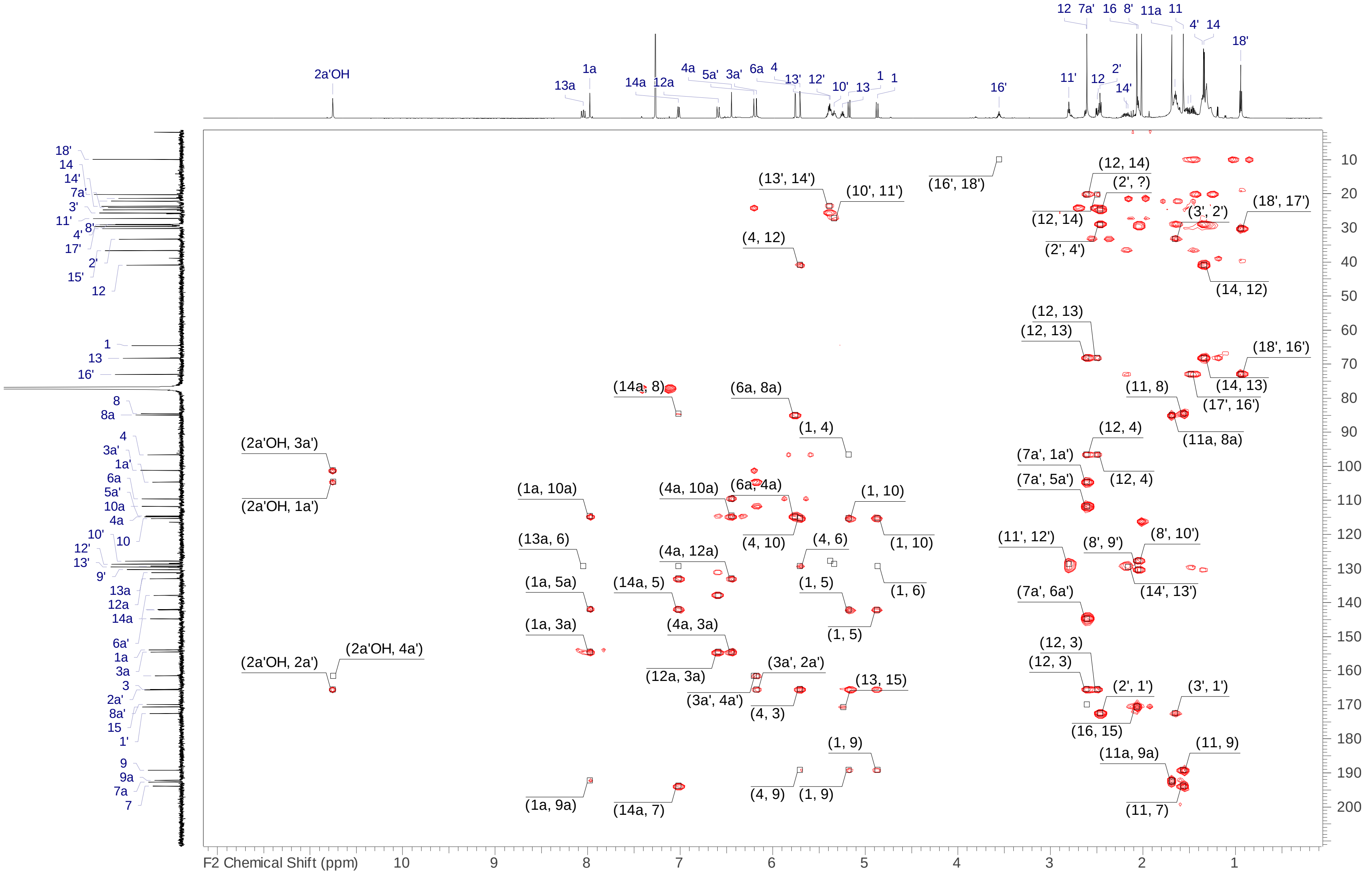

Figure S14: $^{1}H/^{13}C$ HMBC spectrum (500 MHz, $CDCl_3$) of hybridorubrin B (**2**).

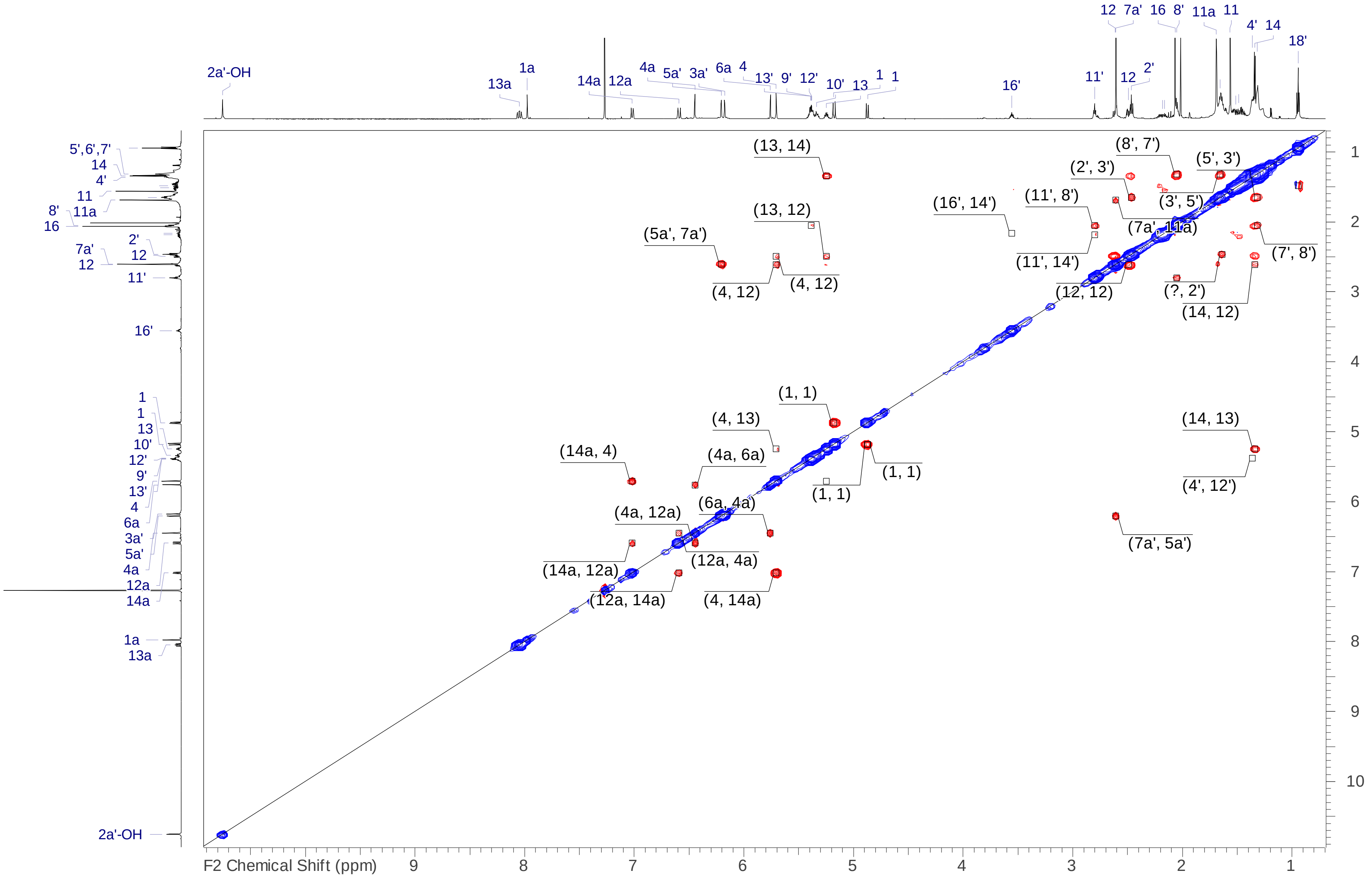

Figure S15: $^{1}H/^{1}H$ ROESY spectrum (500 MHz, $CDCl_3$) of hybridorubrin B (**2**).

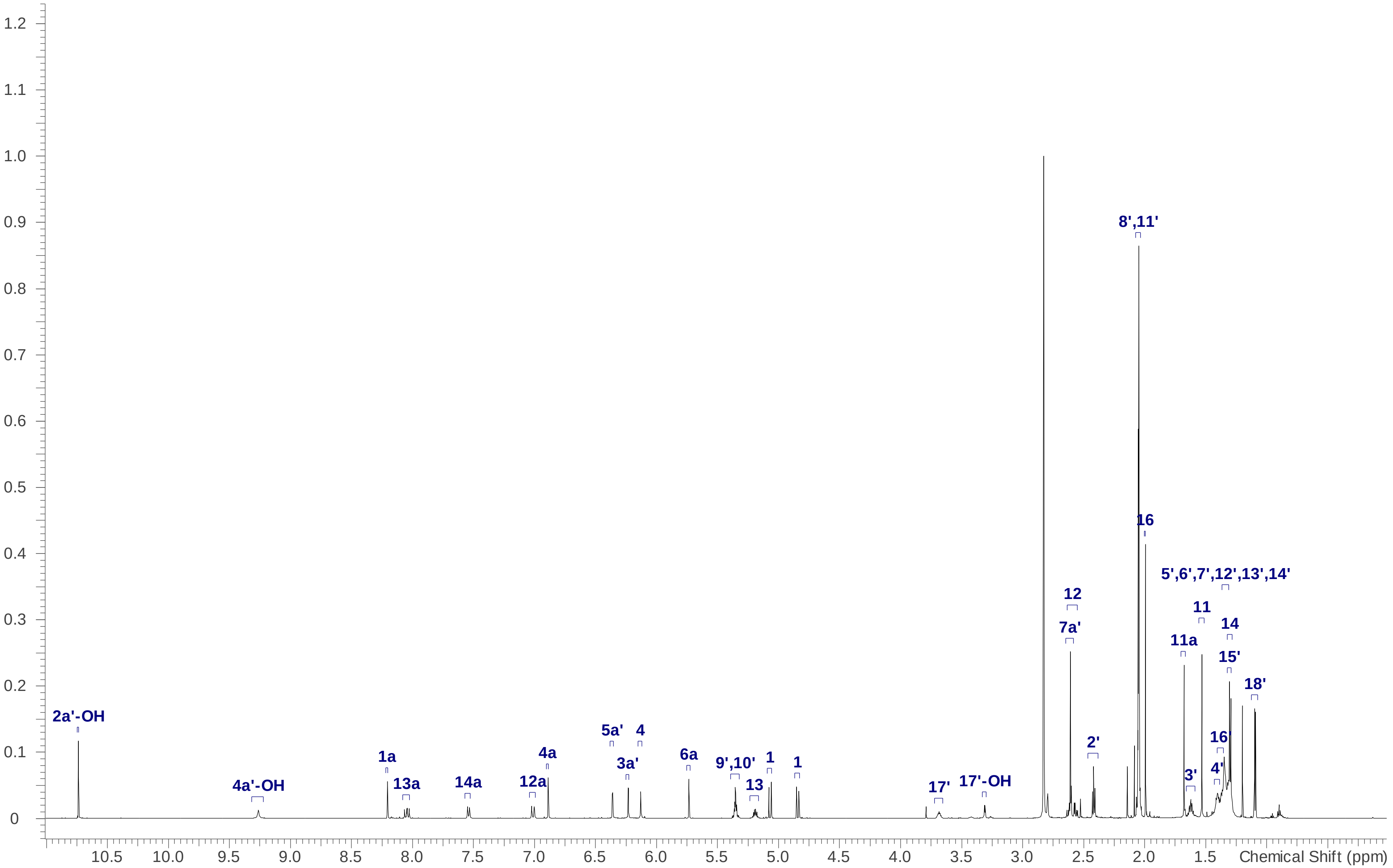

Figure S16: ^{1}H NMR spectrum (700 MHz, acetone-*d6*) of hybridorubrin C (**3**).

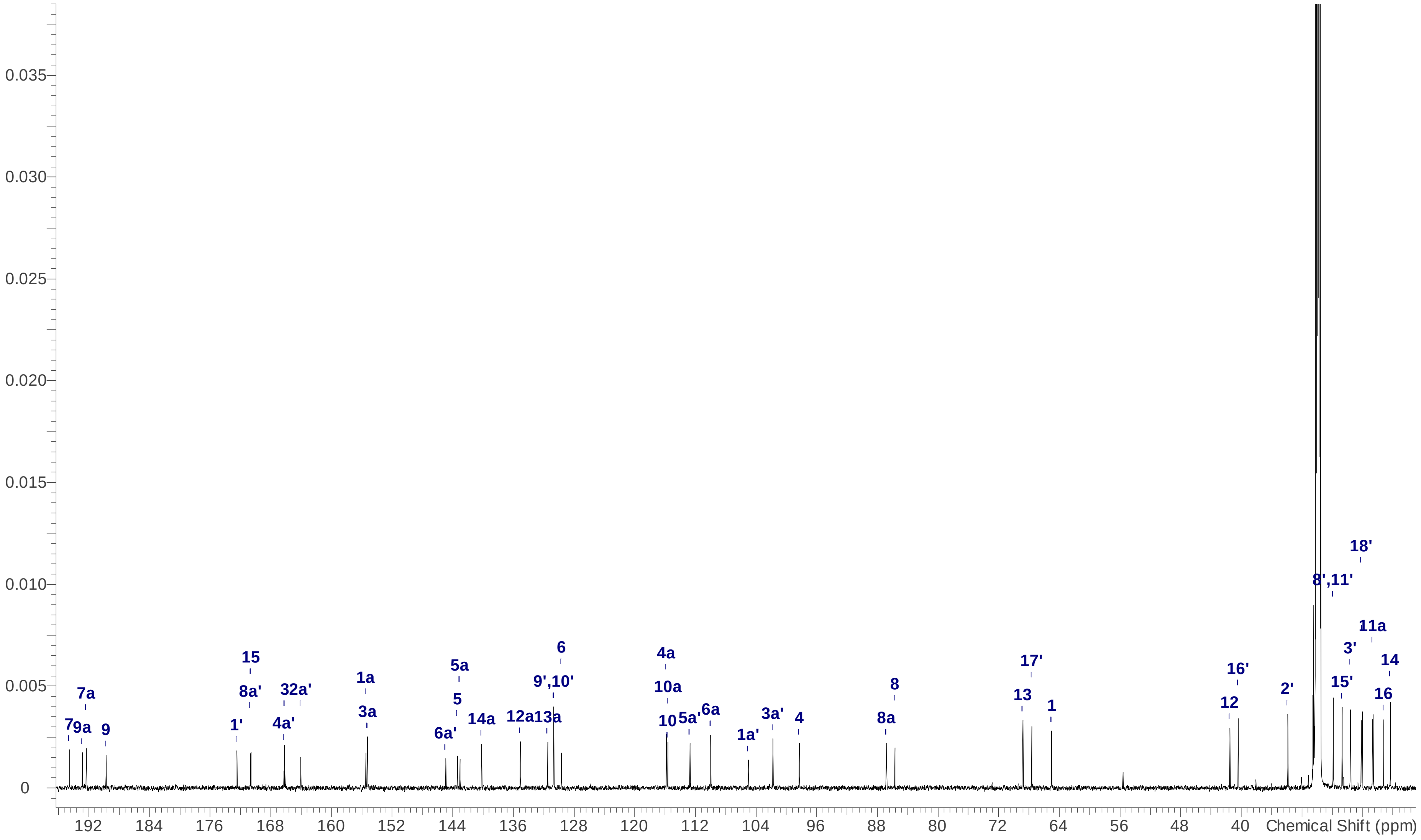

Figure S17: ^{13}C NMR spectrum (175 MHz, acetone-*d6*) of hybridorubrin C (**3**).

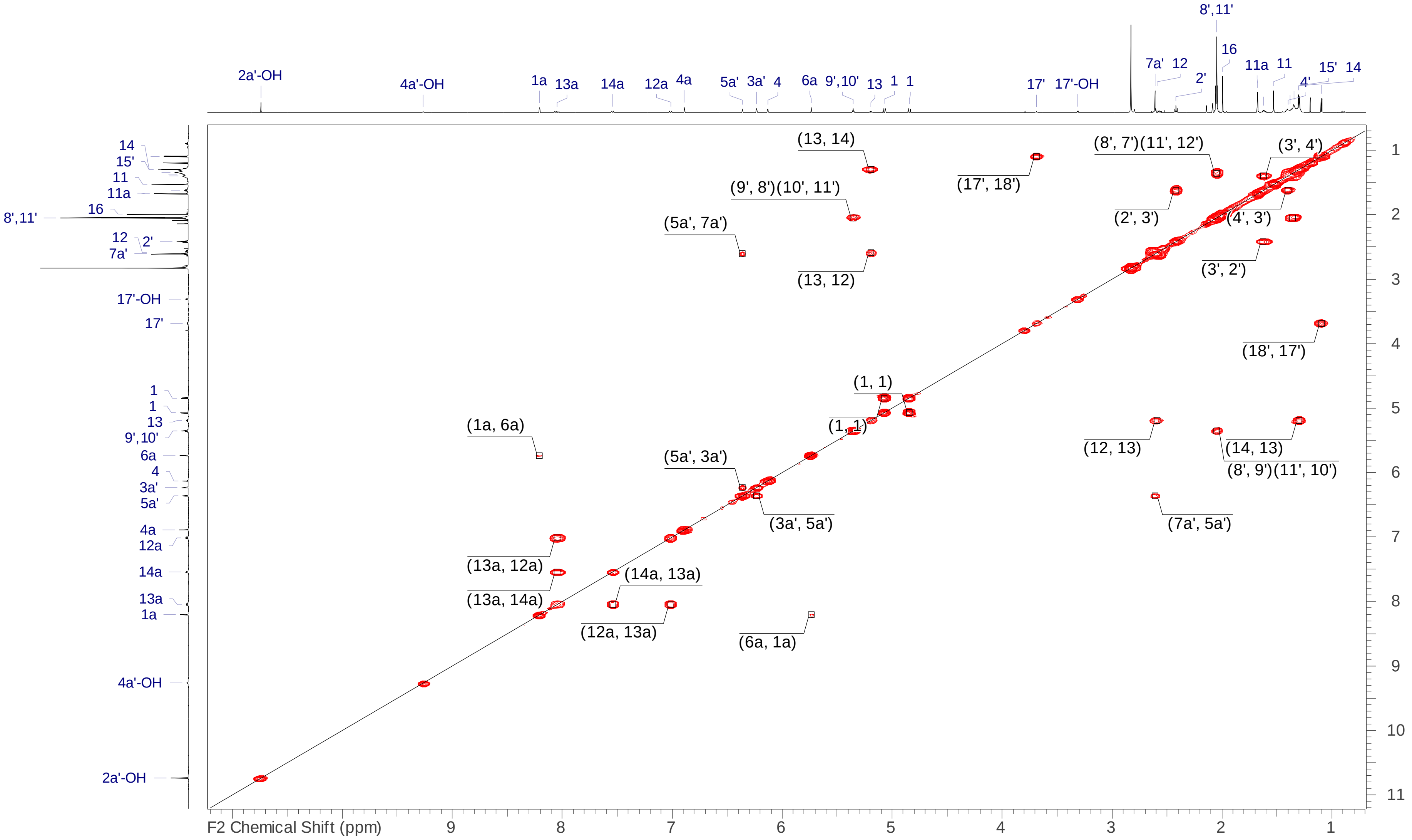

Figure S18: $^{1}H/^{1}H$ COSY spectrum (700 MHz, acetone-*d6*) of hybridorubrin C (**3**).

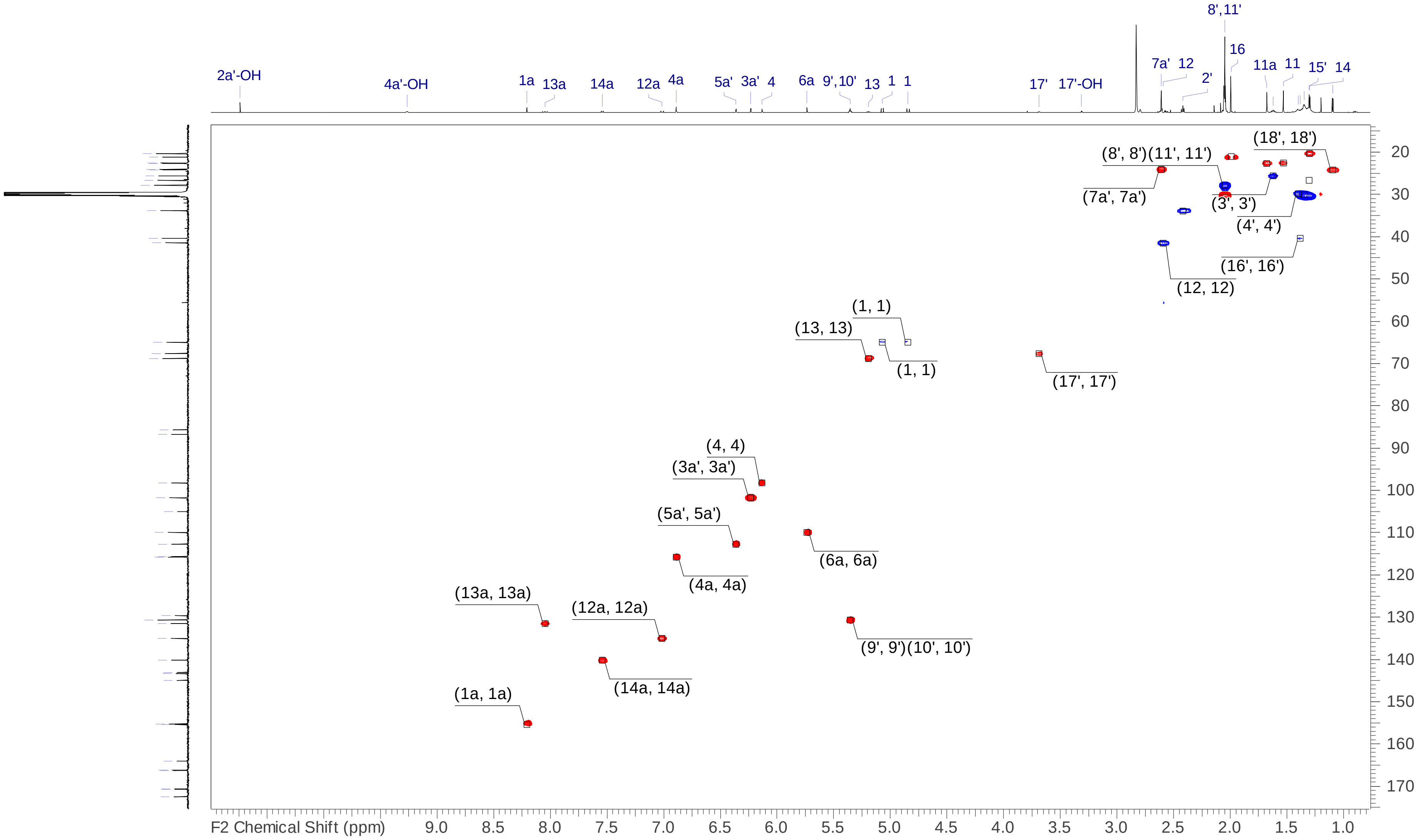

Figure S19: $^{1}H/^{13}C$ HSQC spectrum (700 MHz, acetone-*d6*) of hybridorubrin C (**3**).

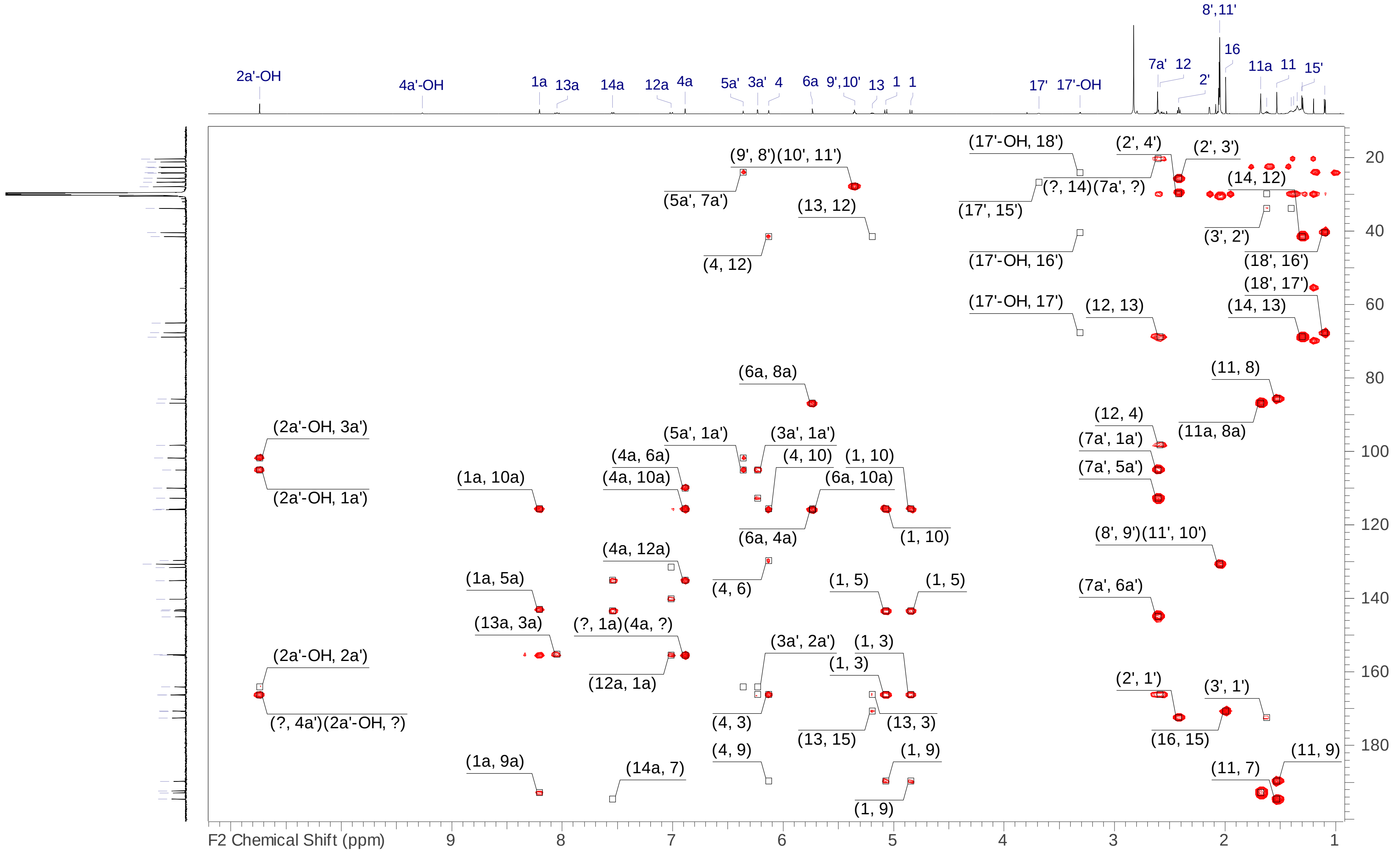

Figure S20: $^{1}H/^{13}C$ HMBC spectrum (700 MHz, acetone-*d6*) of hybridorubrin C (**3**).

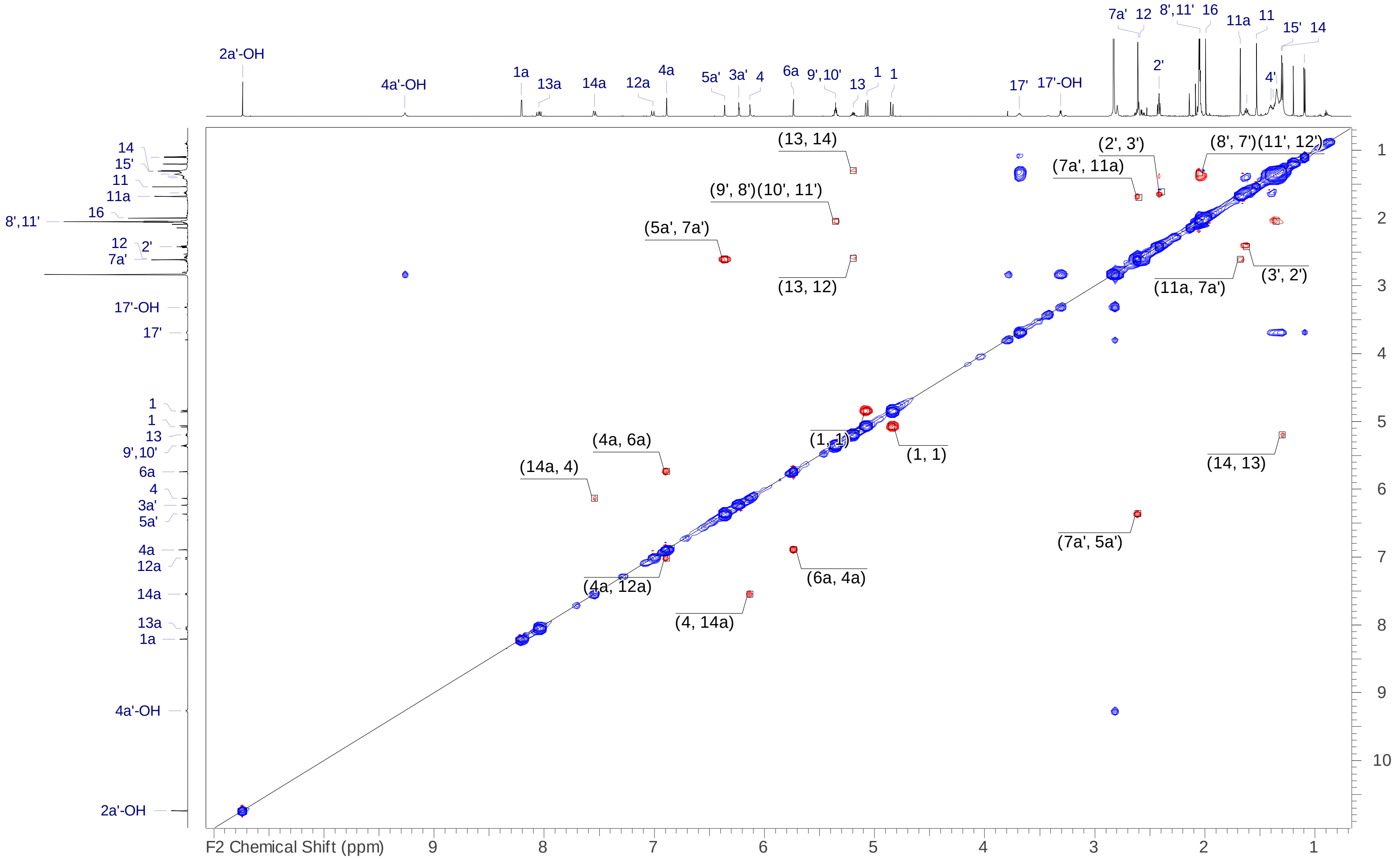

Figure S21: $^{1}H/^{1}H$ ROESY spectrum (700 MHz, acetone-*d6*) of hybridorubrin C (**3**).

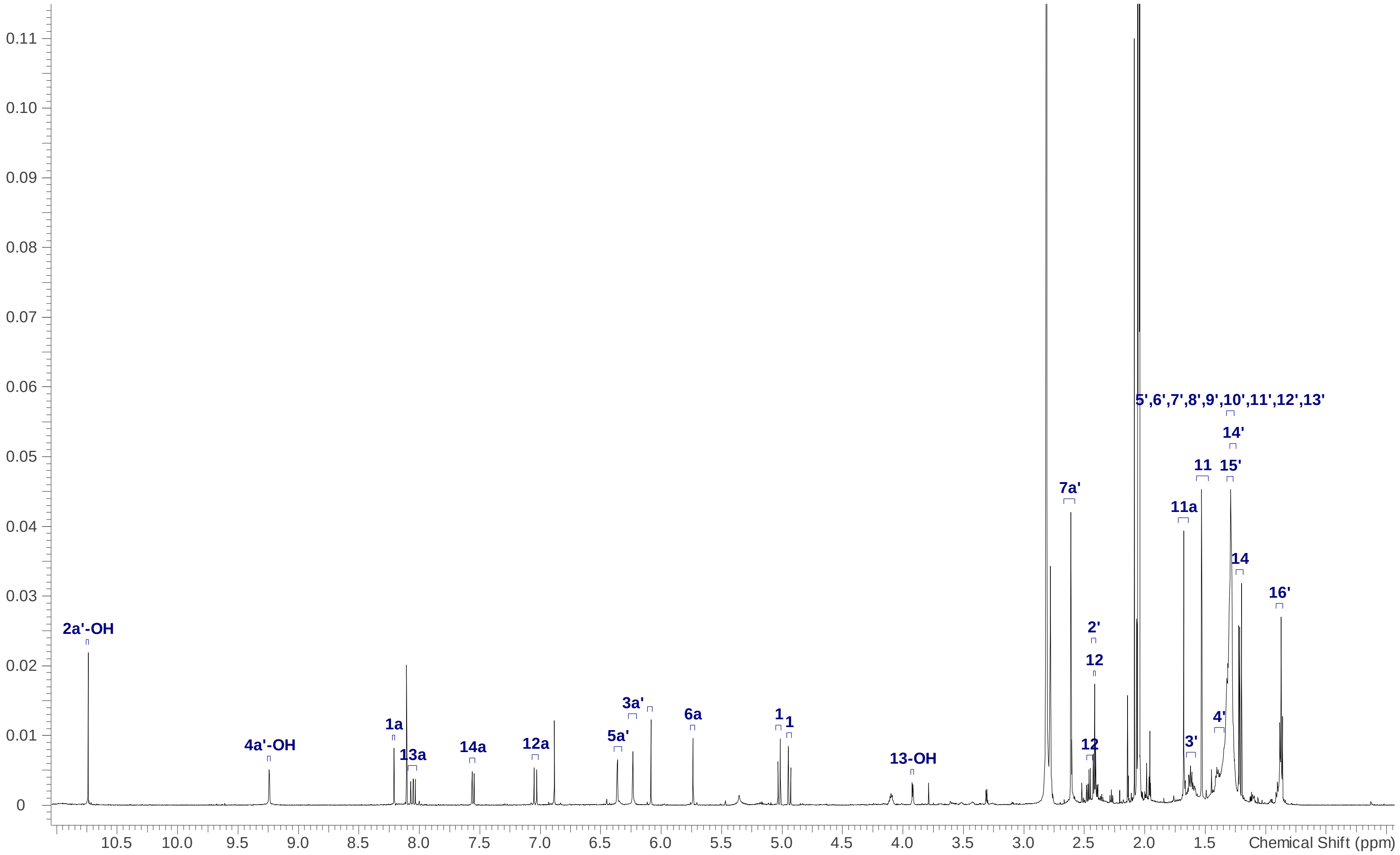

Figure S22: ^{1}H NMR spectrum (700 MHz, acetone-*d*6) of hybridorubrin D (**4**).

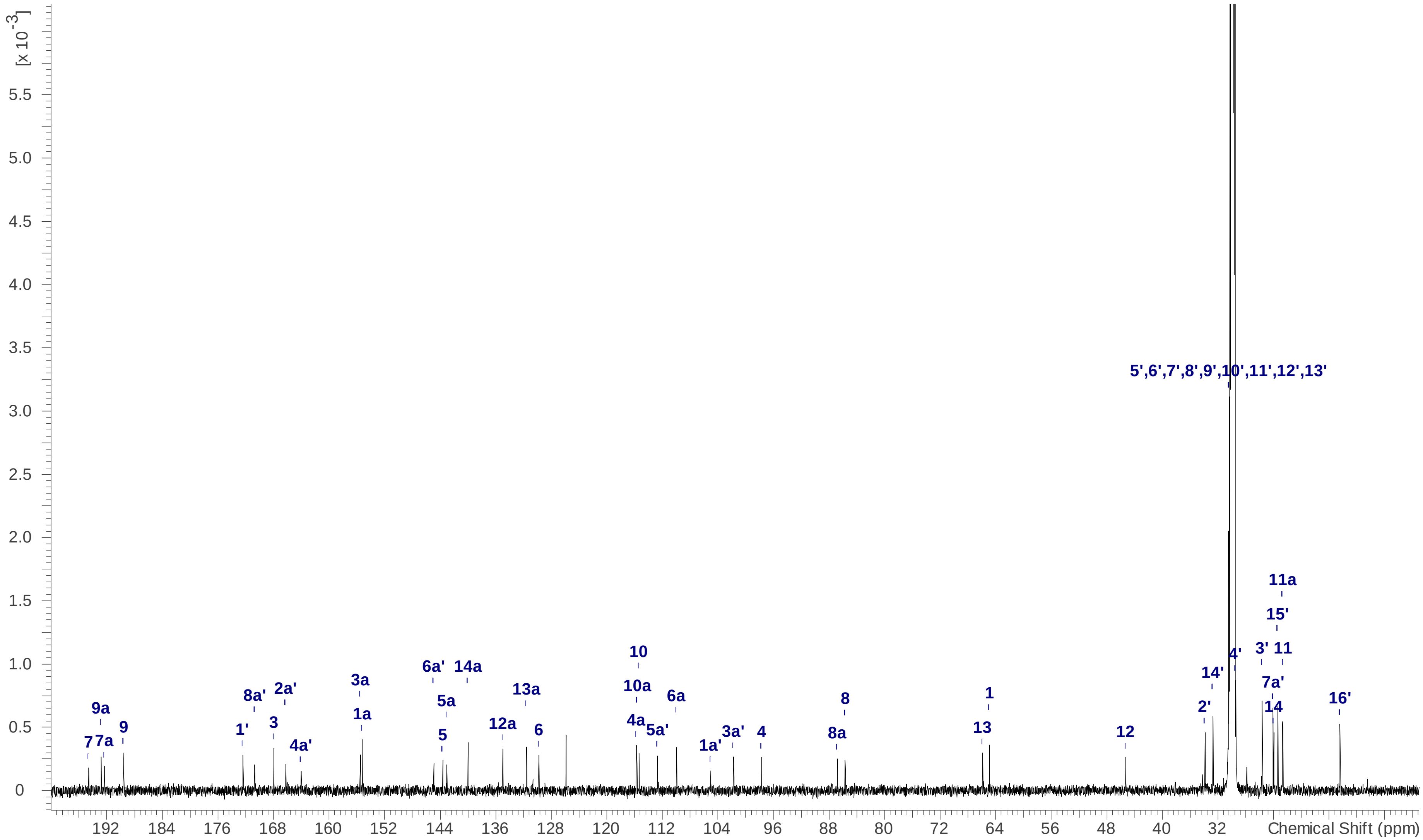

Figure S23: ^{13}C NMR spectrum (175 MHz, acetone-*d*6) hybridorubrin D (**4**).

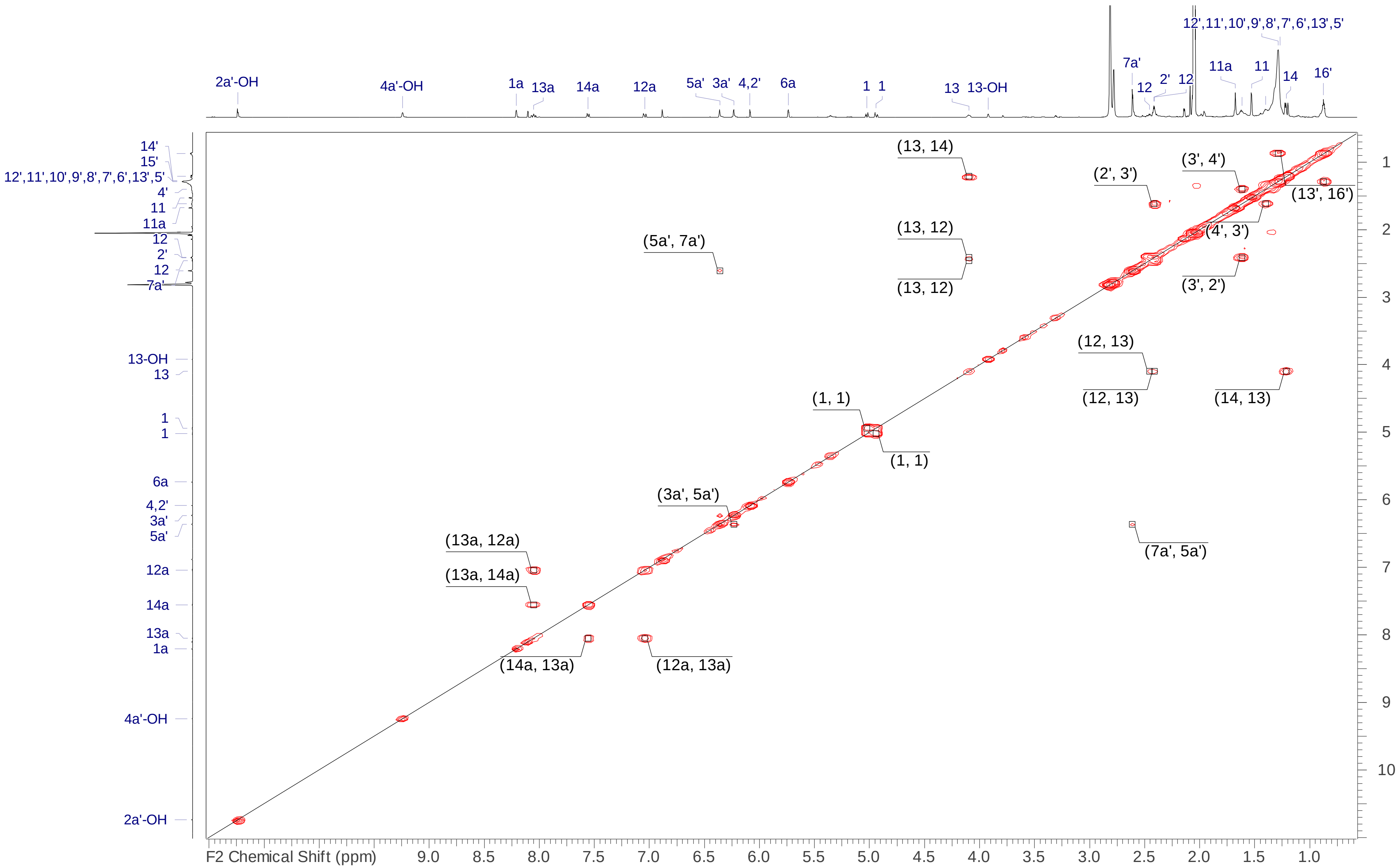

Figure S24: $^1H/^1H$ COSY spectrum (700 MHz, acetone-*d*6) of hybridorubrin D (**4**).

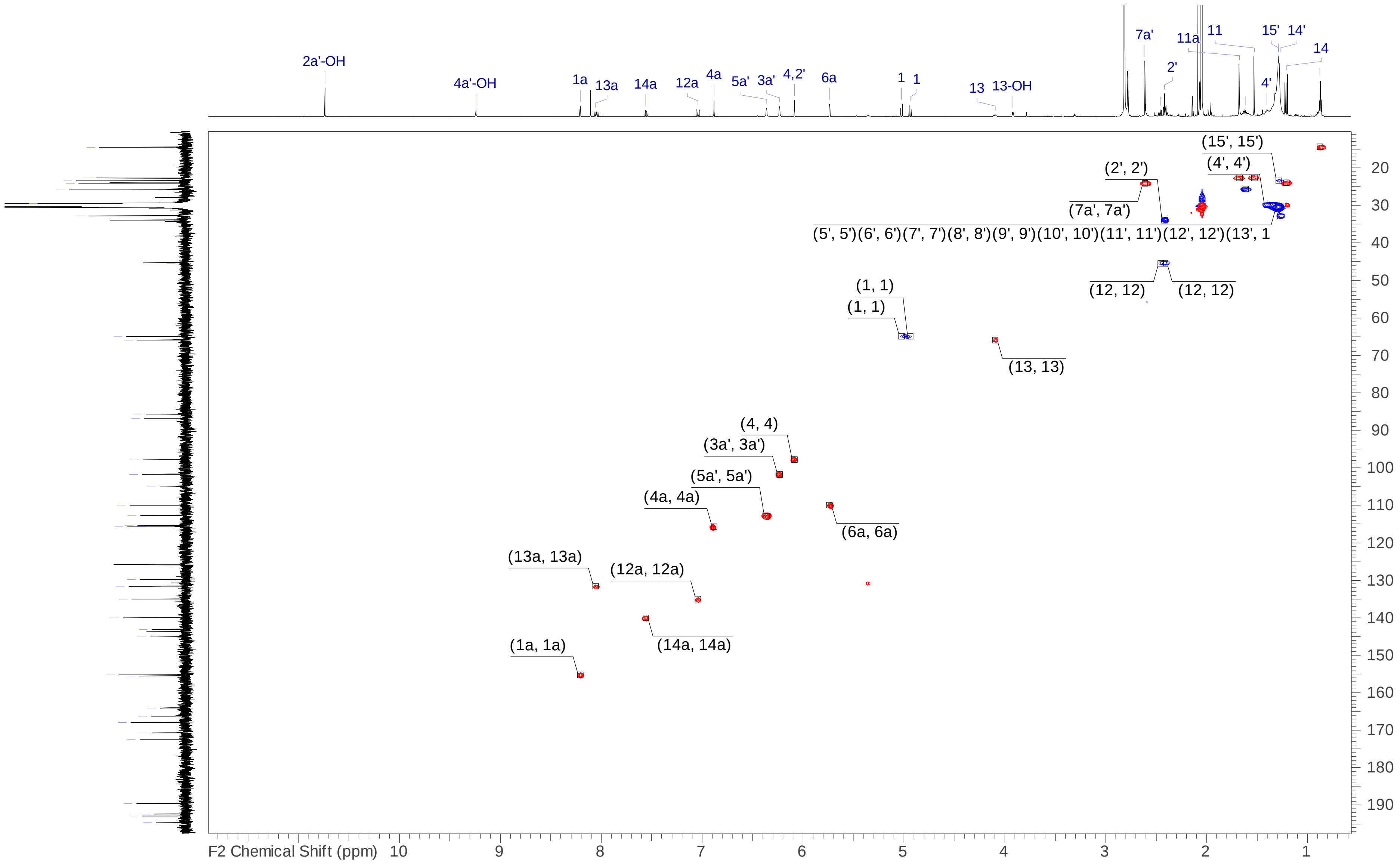

Figure S25: $^{1}H/^{13}C$ HSQC spectrum (700 MHz, acetone-*d*6) of hybridorubrin D (**4**).

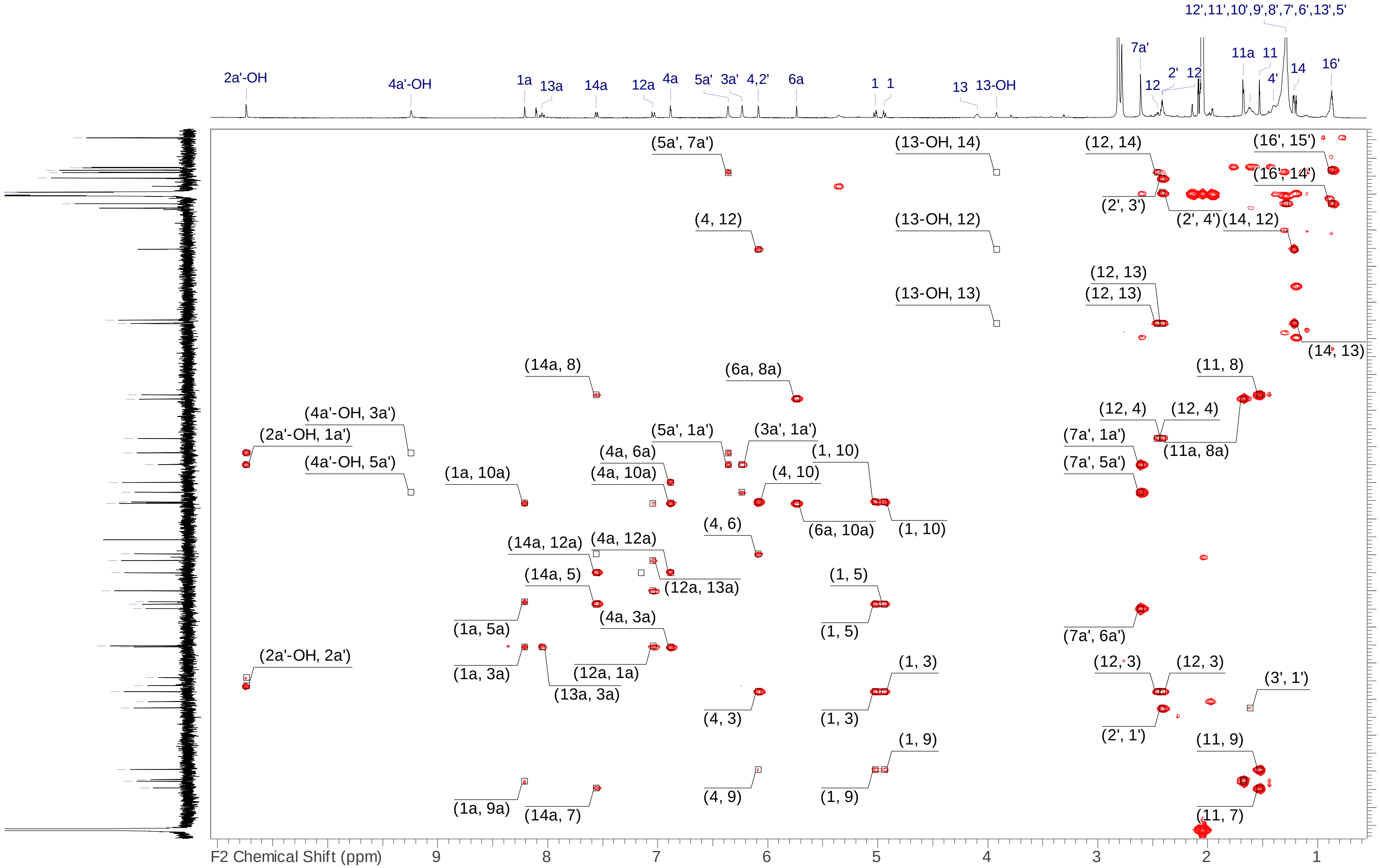

Figure S26: $^{1}H/^{13}C$ HMBC spectrum (700 MHz, acetone-*d*6) of hybridorubrin D (**4**).

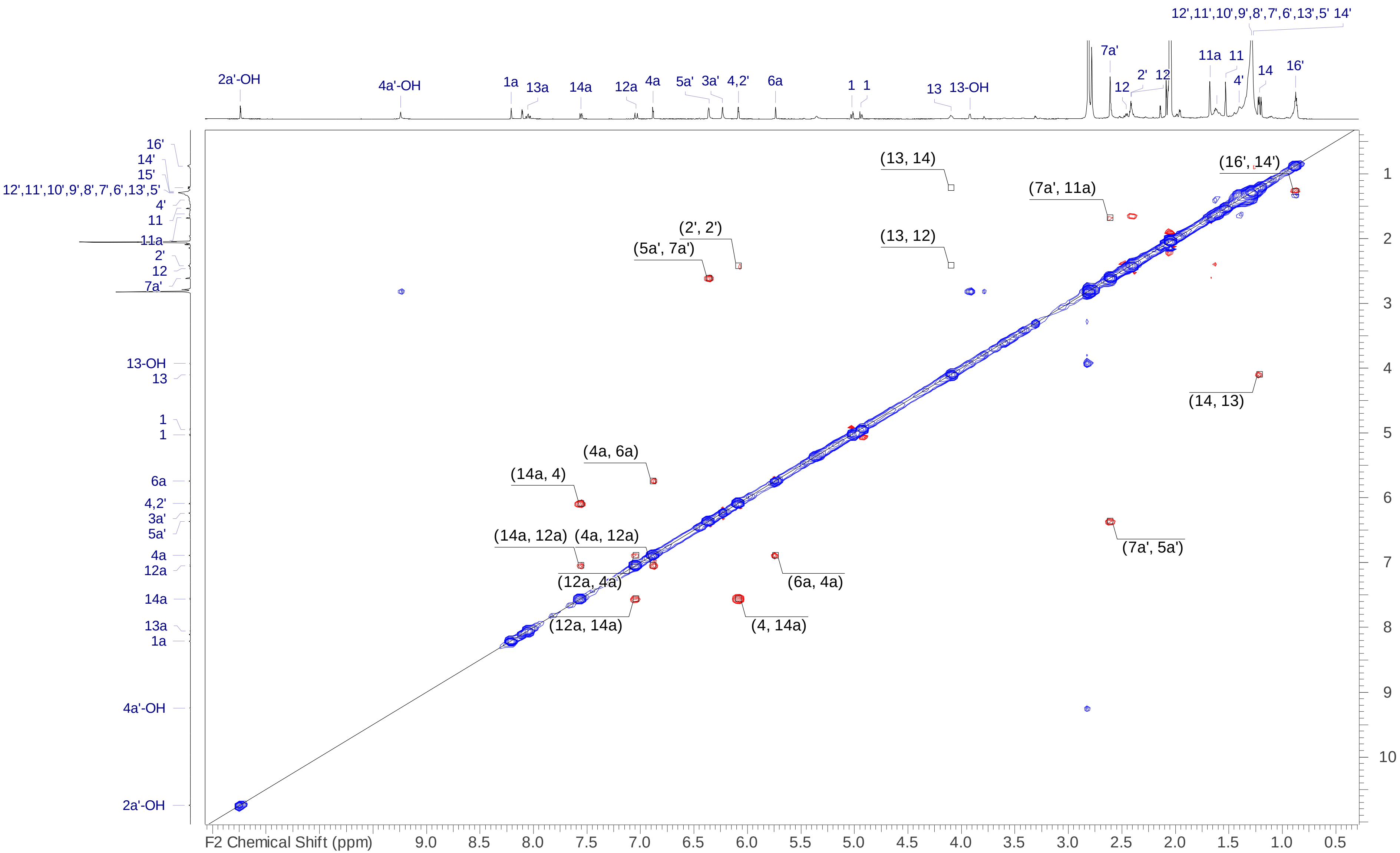

Figure S27: ^{1}H/^{1}H ROESY spectrum (700 MHz, acetone-*d*6) of hybridorubrin D (**4**).

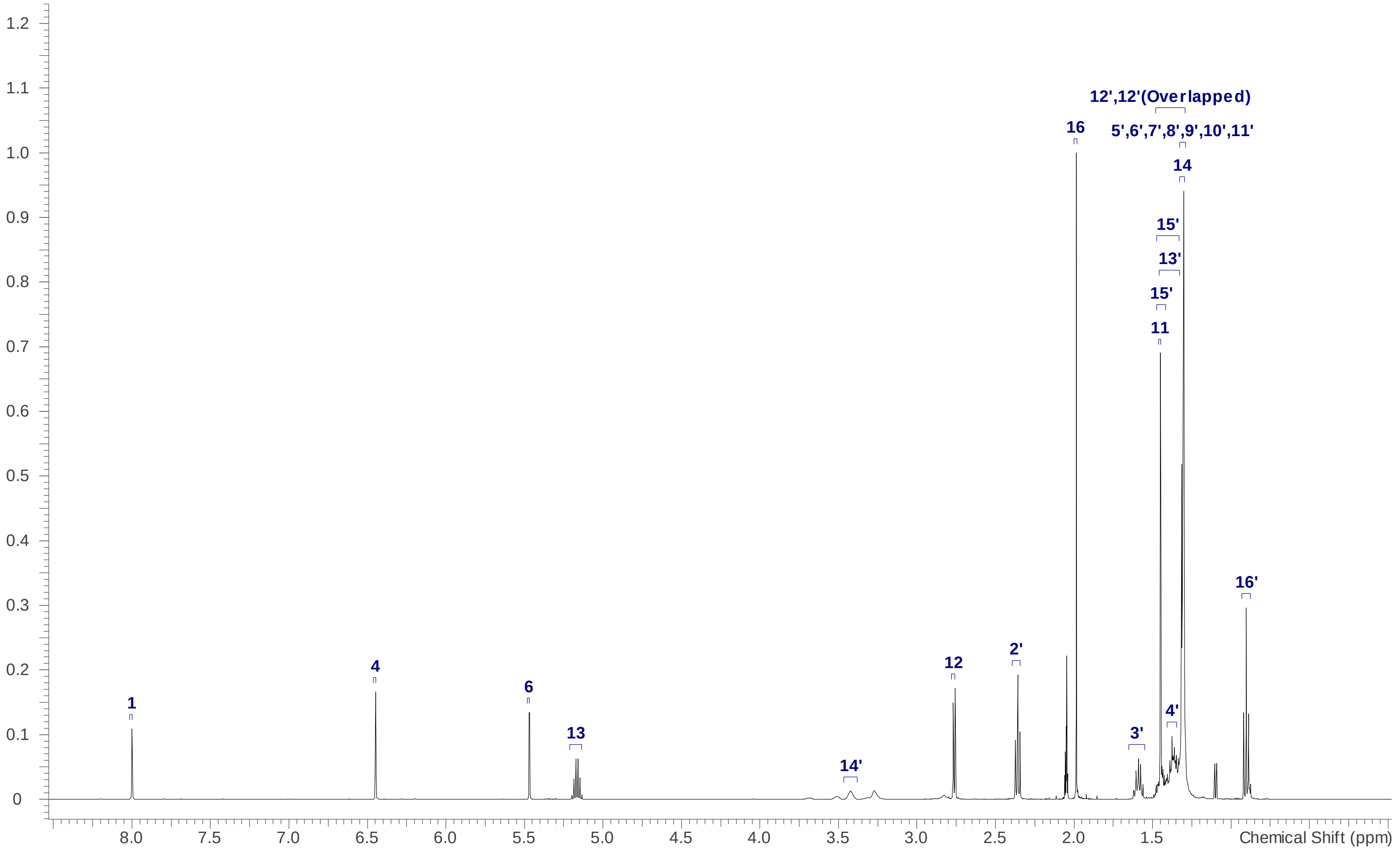

Figure S28: ^{1}H NMR spectrum (500 MHz, acetone-*d*6) of fragirubrin F (**5**).

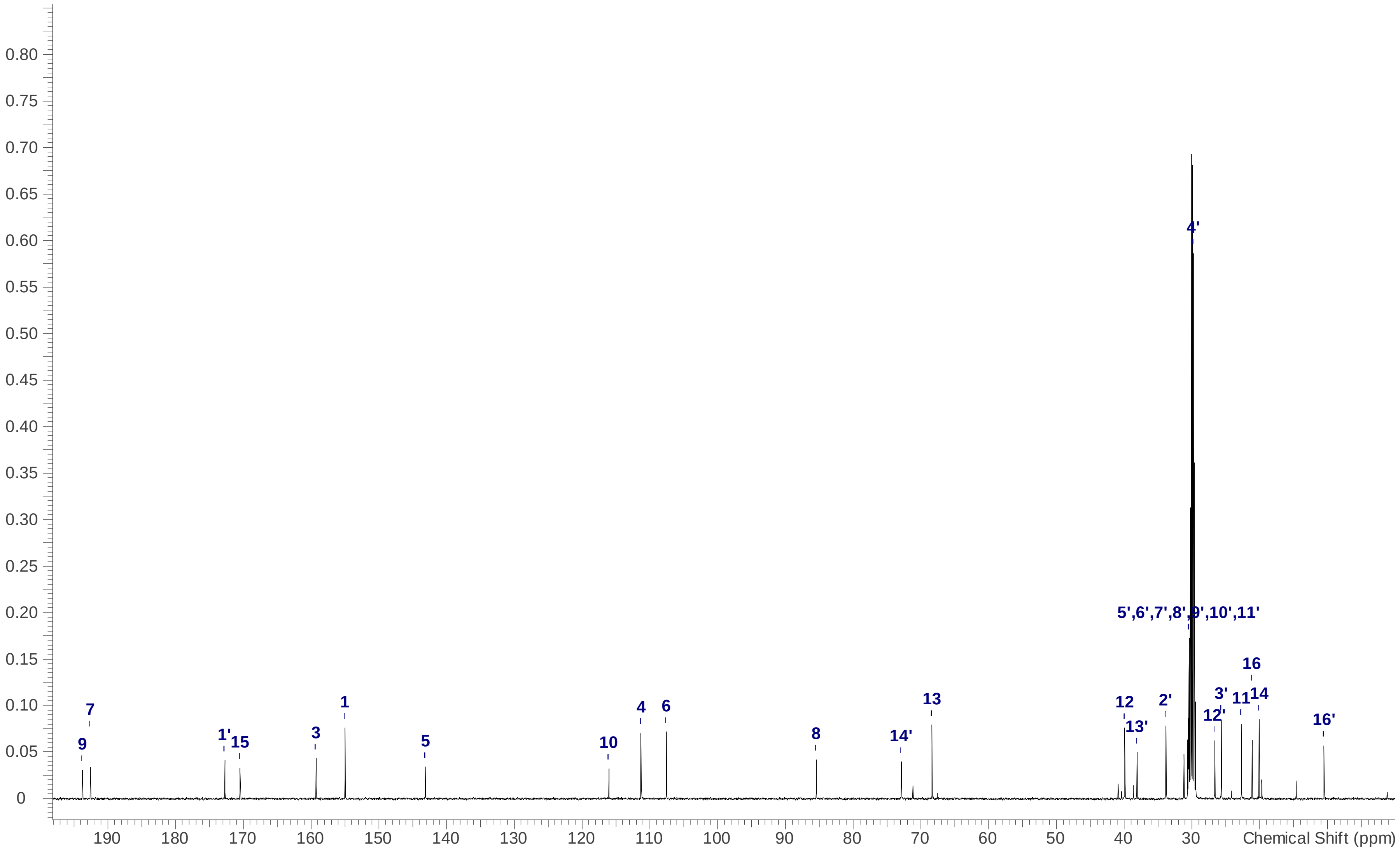

Figure S29: ^{13}C NMR spectrum (125 MHz, acetone-*d*6) of fragirubrin F (**5**).

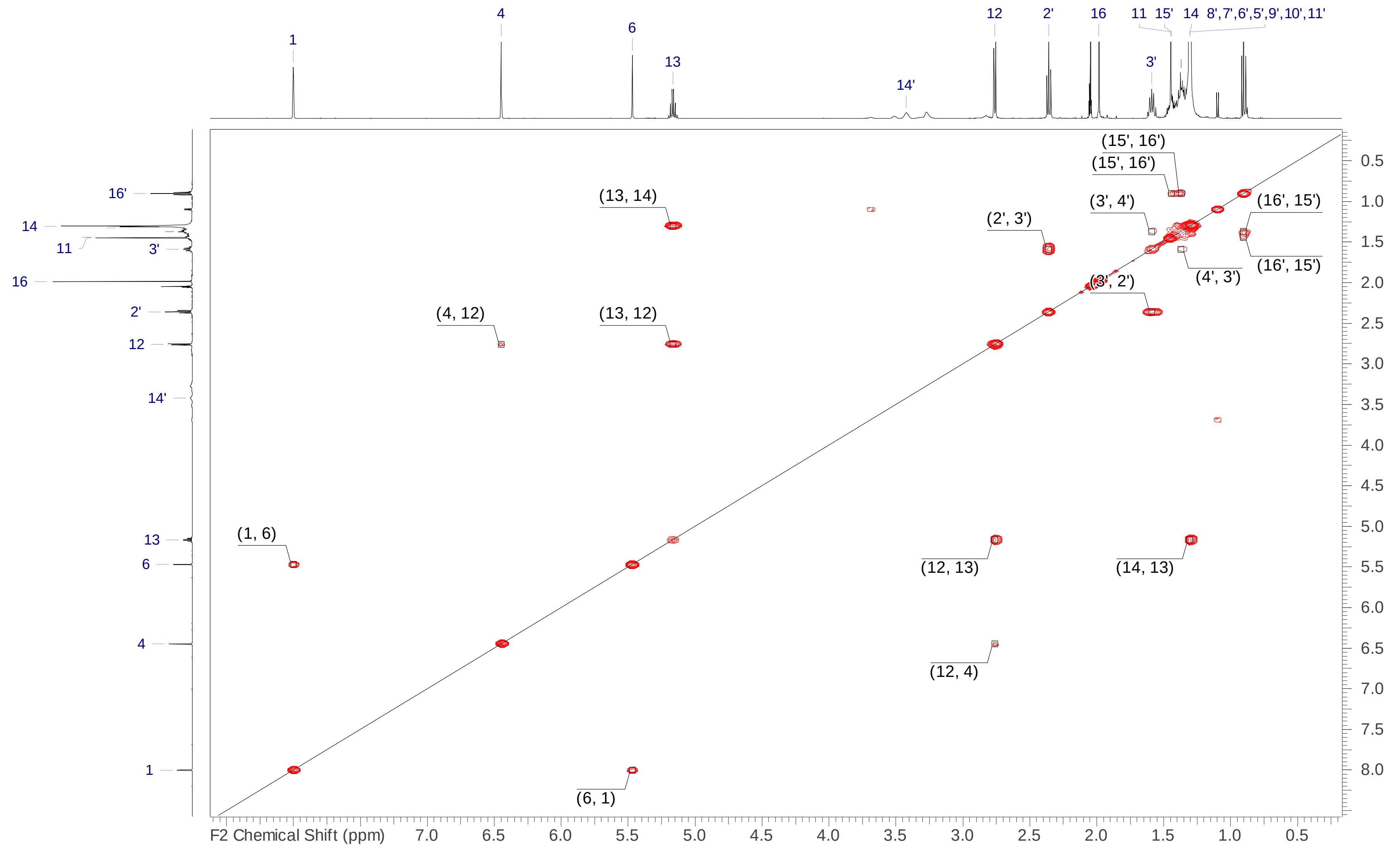

Figure S30: $^1H/^1H$ COSY spectrum (500 MHz, acetone-*d*6) of fragirubrin F (**5**).

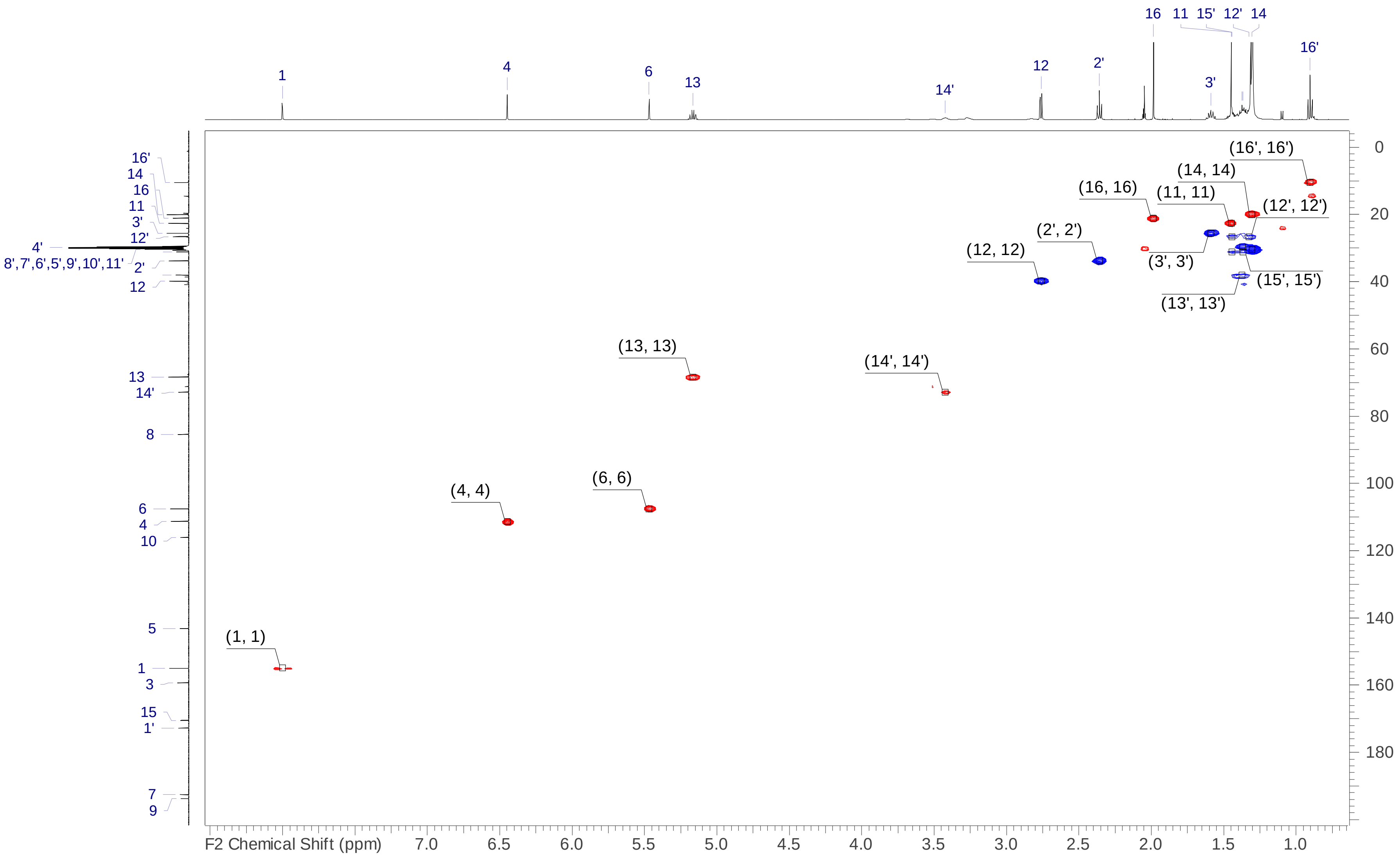

Figure S31: $^{1}H/^{13}C$ HSQC spectrum (500 MHz, acetone-*d*6) of fragirubrin F (**5**).

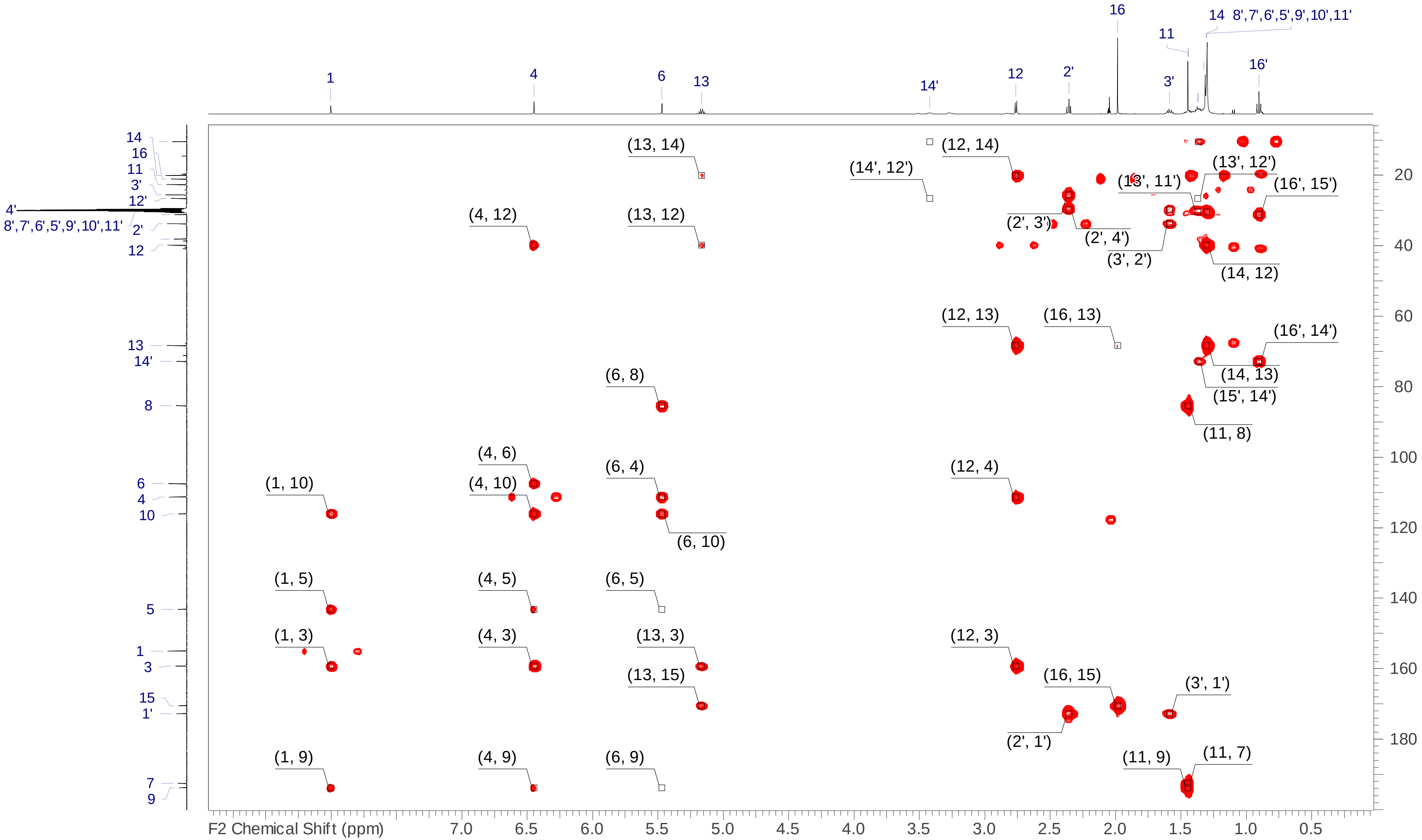

Figure S32: ^{1}H/^{13}C HMBC spectrum (500 MHz, acetone-*d6*) of fragirubrin F (**5**).

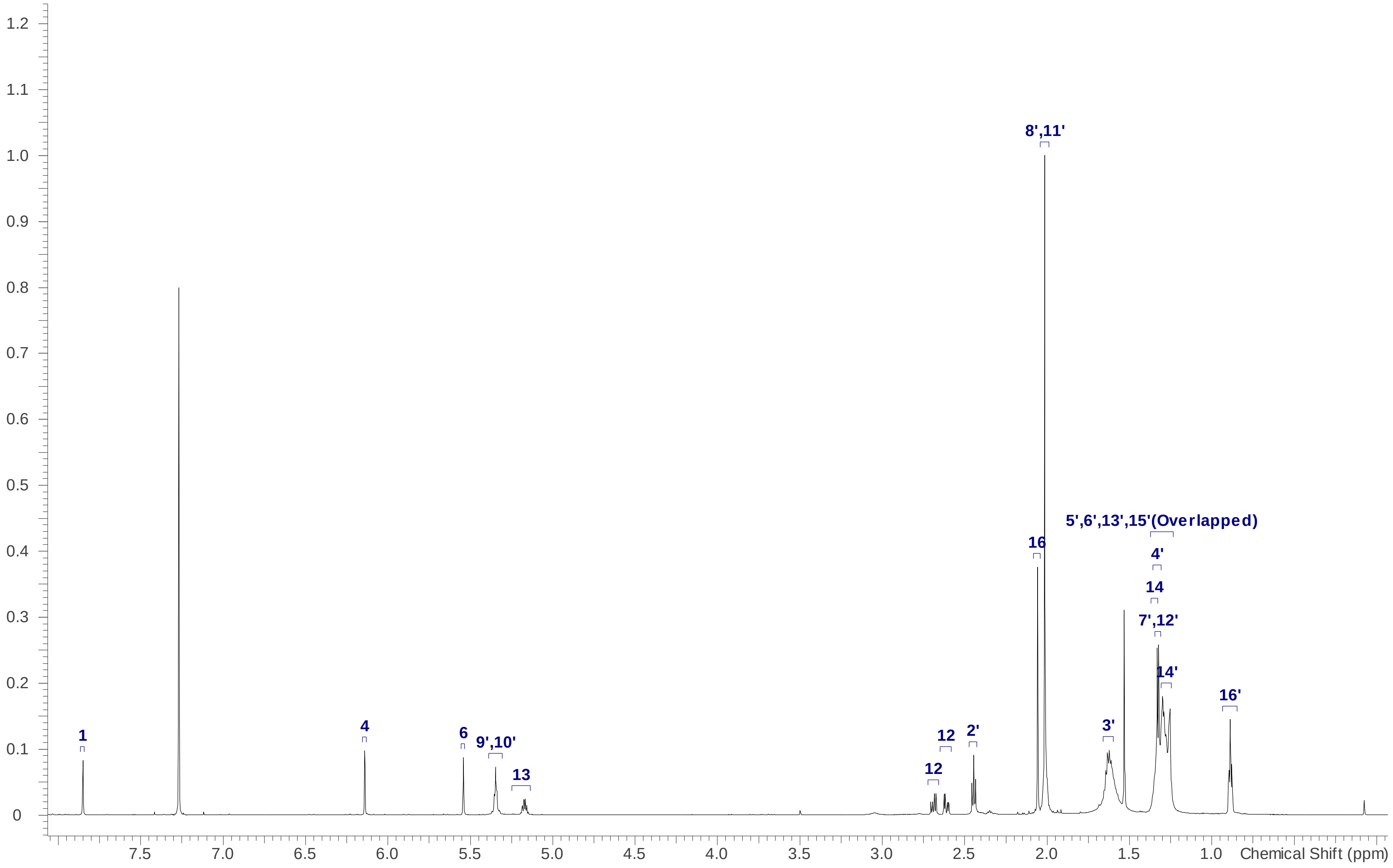

Figure S33: ^{1}H NMR spectrum (700 MHz, $CDCl_3$) of fragirubrin G (**6**).

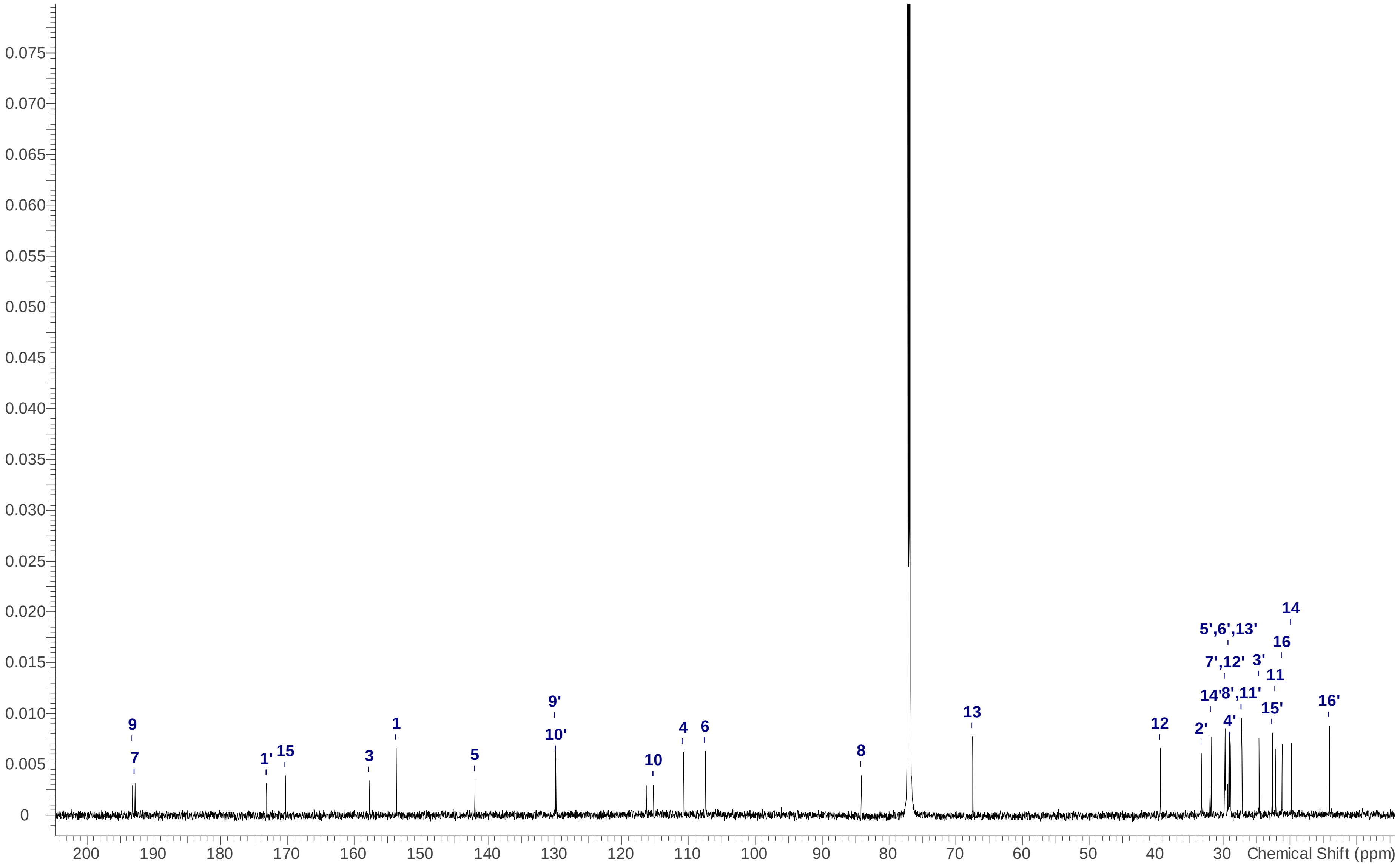

Figure S34: ^{13}C NMR spectrum (175 MHz, $CDCl_3$) of fragirubrin G (**6**).

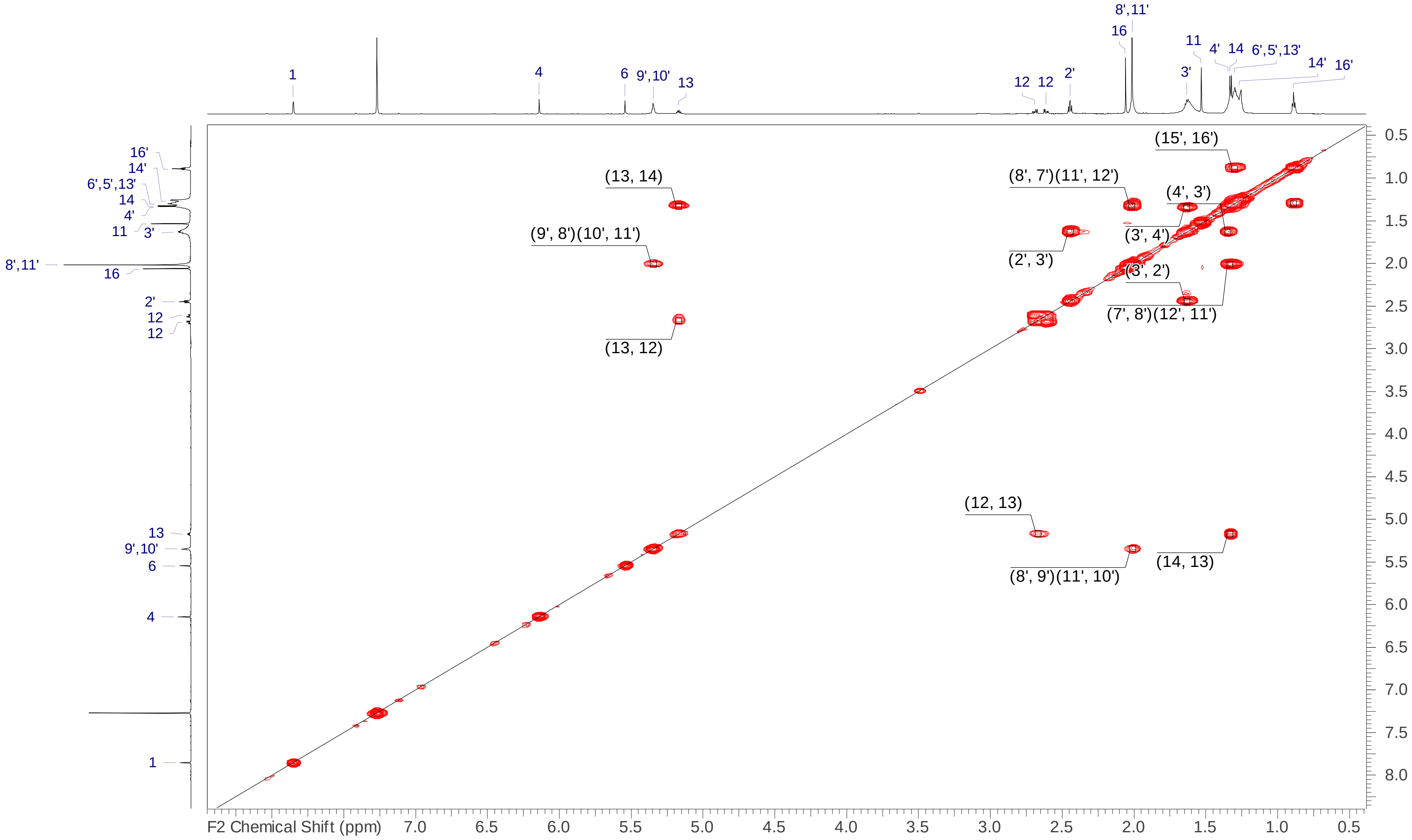

Figure S35: $^1H/^1H$ COSY spectrum (700 MHz, $CDCl_3$) of fragirubrin G (**6**).

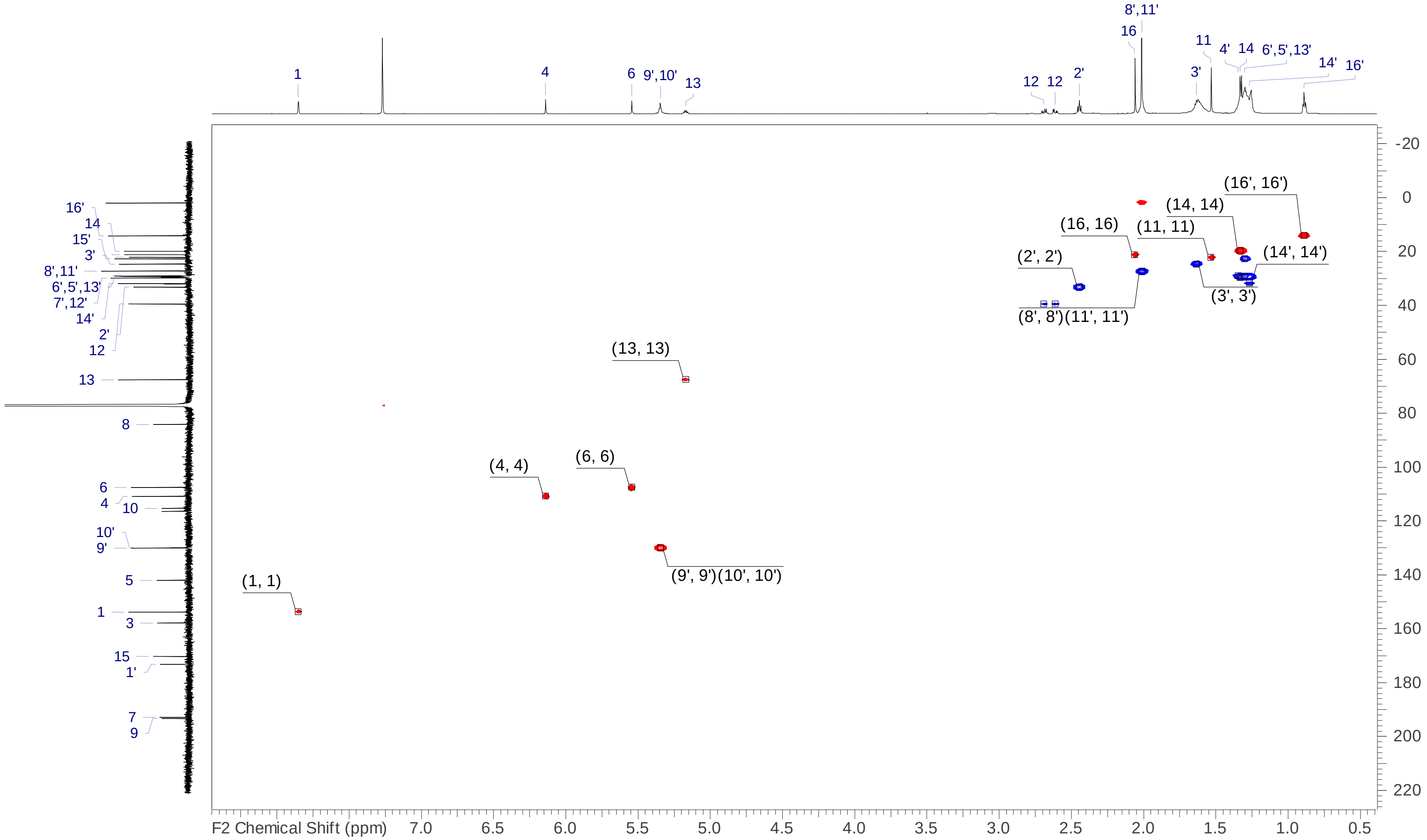

Figure S36: $^{1}H/^{13}C$ HSQC spectrum (700 MHz, $CDCl_3$) of fragirubrin G (**6**).

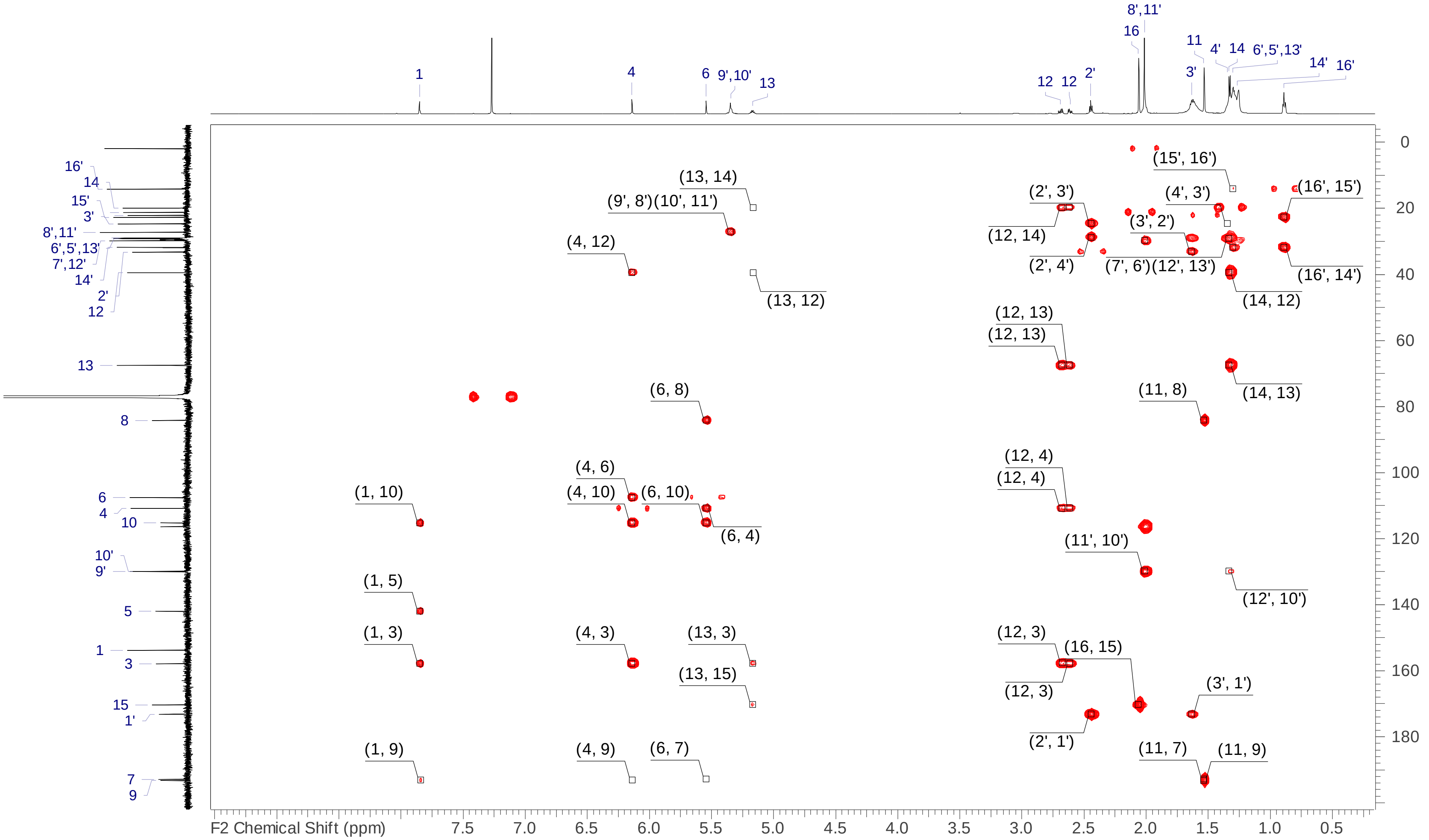

Figure S37: $^{1}H/^{13}C$ HMBC spectrum (700 MHz, $CDCl_3$) of fragirubrin G (**6**).

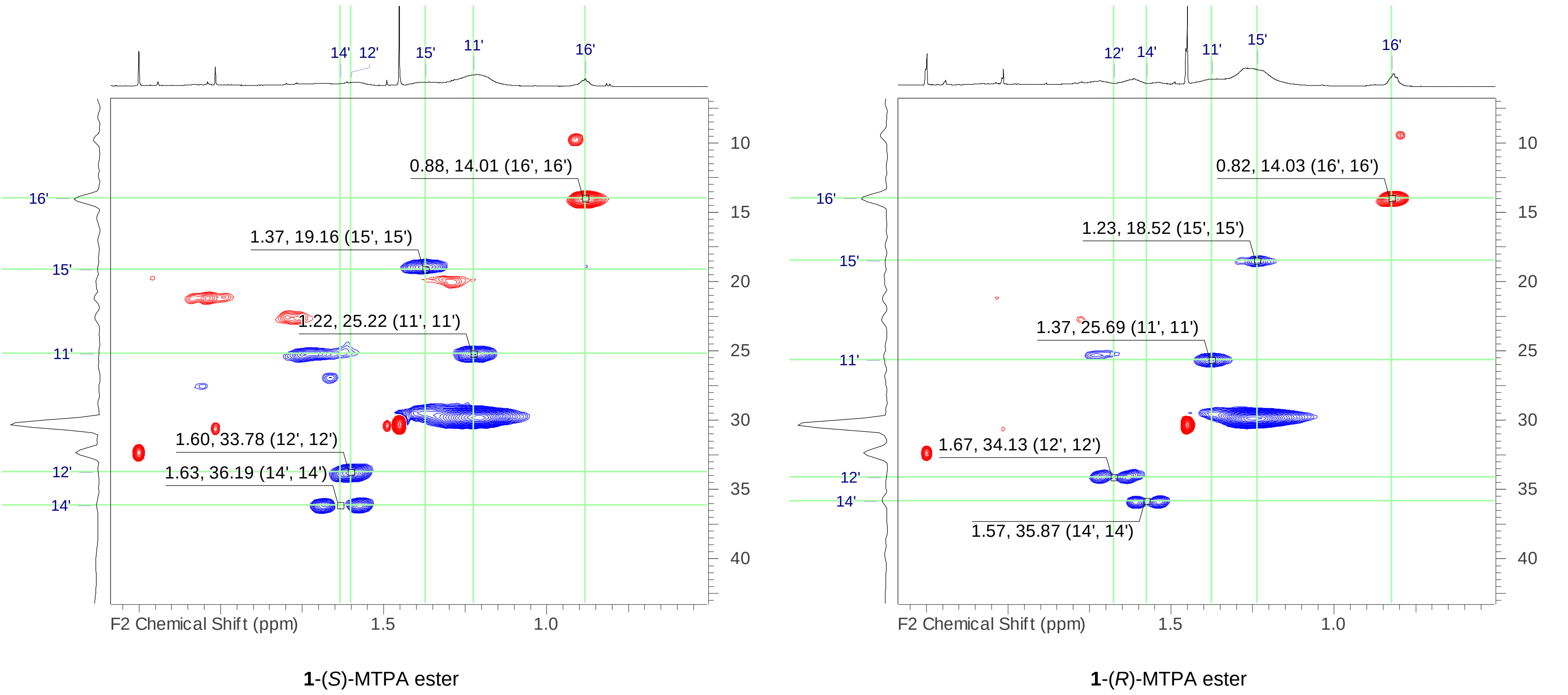

Figure S38: $^{1}H/^{13}C$ HSQC spectra of hybridorubrin A (**1**) after esterification with (*R*)-MTPA chloride (yielding the (*S*)-MTPA ester; left) and (*S*)-MTPA chloride (yielding the (*R*)-MTPA ester; right). (700 MHz, pyridine-*d5*).

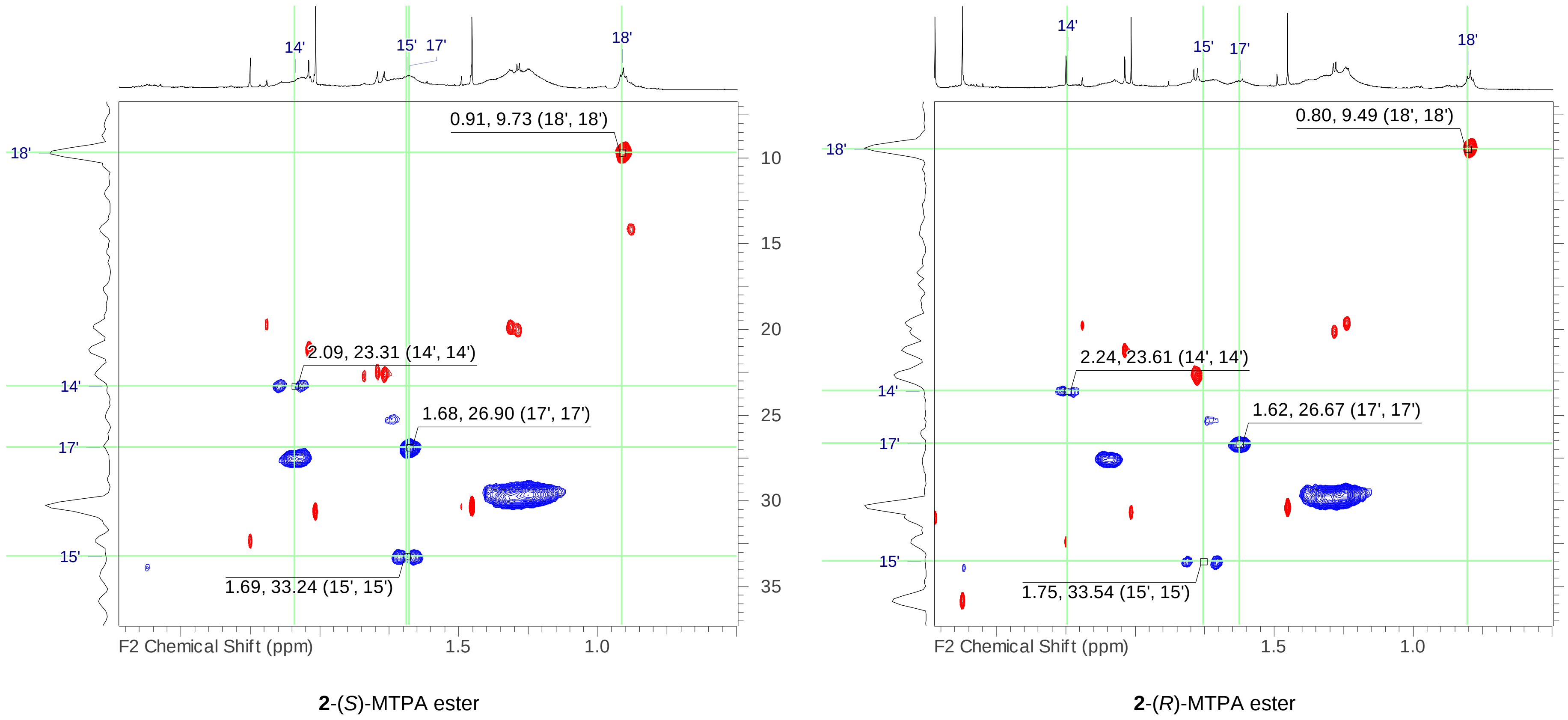

Figure S39: $^{1}H/^{13}C$ HSQC spectra of hybridorubrin B (**2**) after esterification with (*R*)-MTPA chloride (yielding the (*S*)-MTPA ester; left) and (*S*)-MTPA chloride (yielding the (*R*)-MTPA ester; right). (700 MHz, pyridine-*d5*).

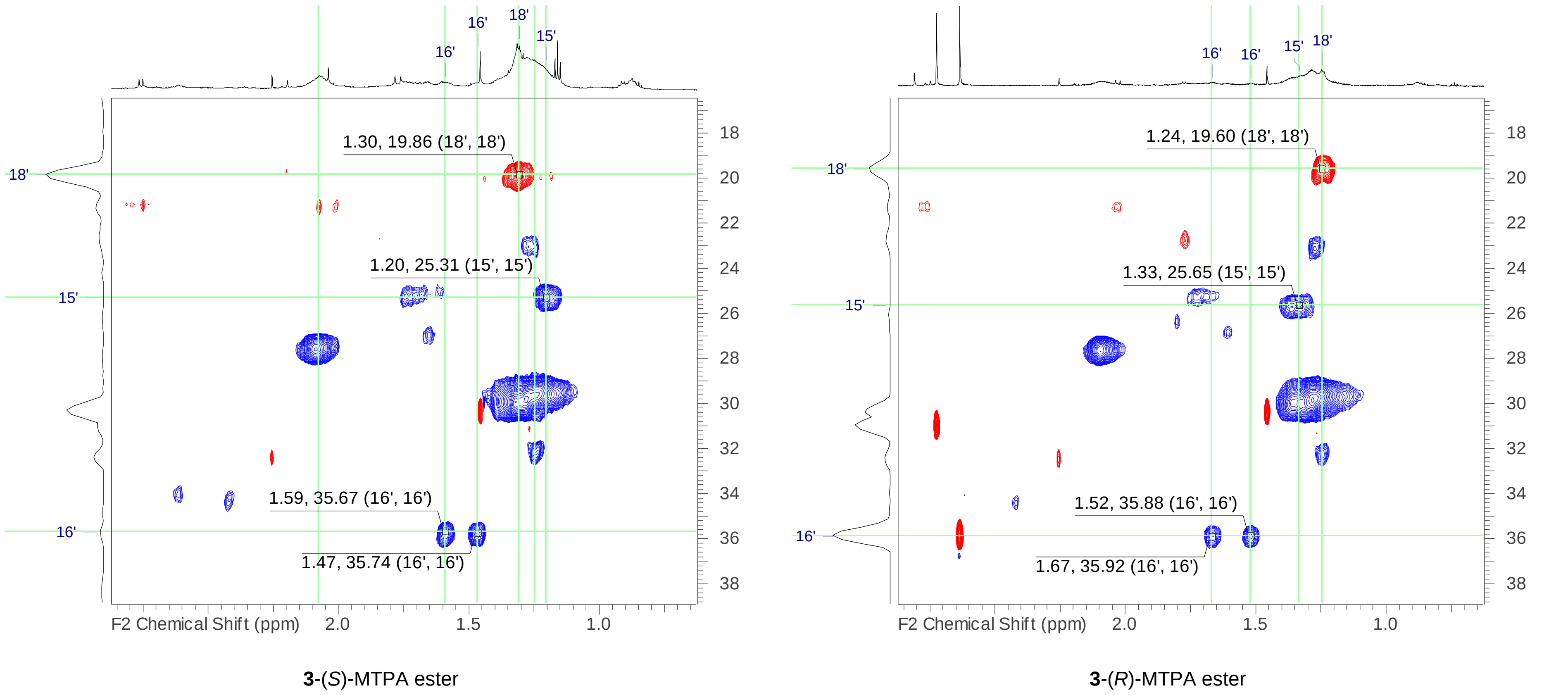

Figure S40: $^{1}H/^{13}C$ HSQC spectra of hybridorubrin C (**3**) after esterification with (*R*)-MTPA chloride (yielding the (*S*)-MTPA ester; left) and (*S*)-MTPA chloride (yielding the (*R*)-MTPA ester; right). (700 MHz, pyridine-*d5*).

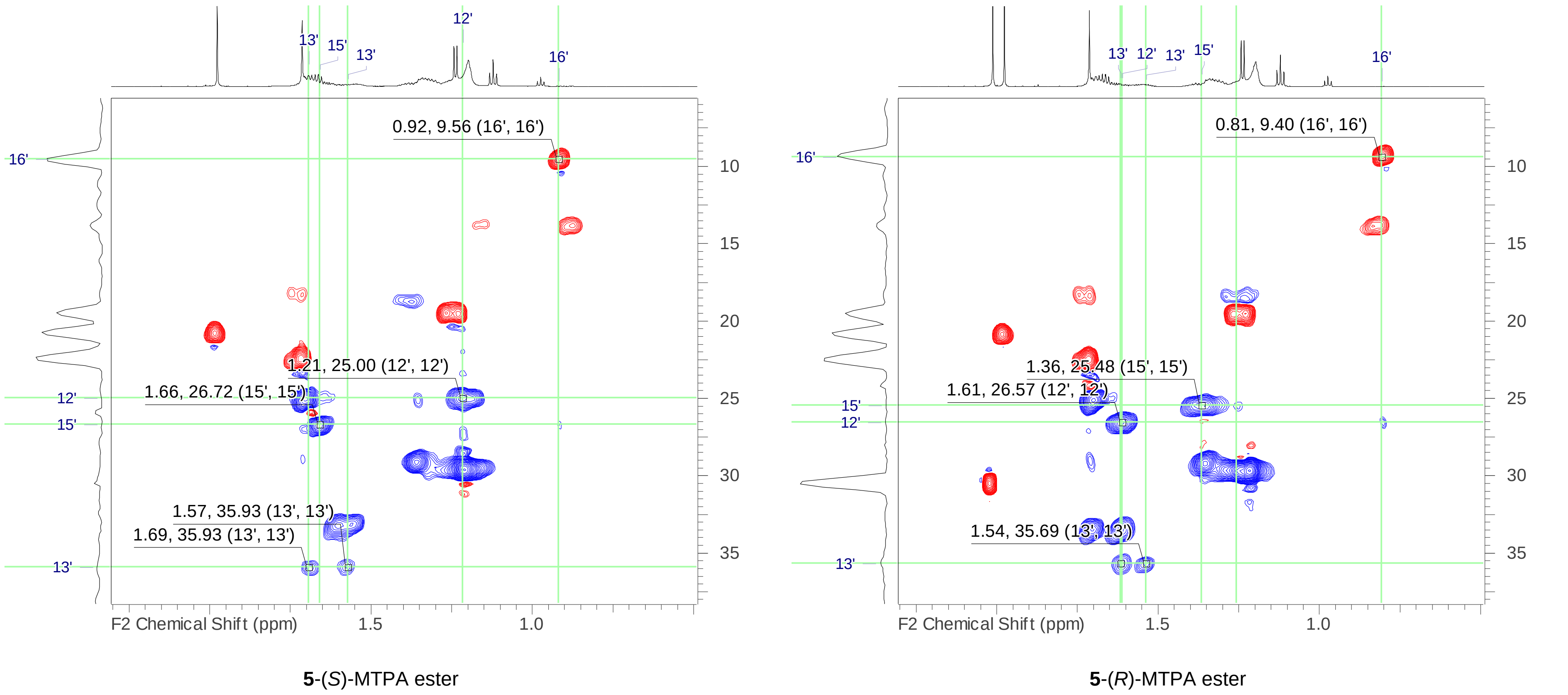

Figure S41: $^{1}H/^{13}C$ HSQC spectra of fragirubrin F (**5**) after esterification with (*R*)-MTPA chloride (yielding the (*S*)-MTPA ester; left) and (*S*)-MTPA chloride (yielding the (*R*)-MTPA ester; right). (700 MHz, pyridine-*d5*).

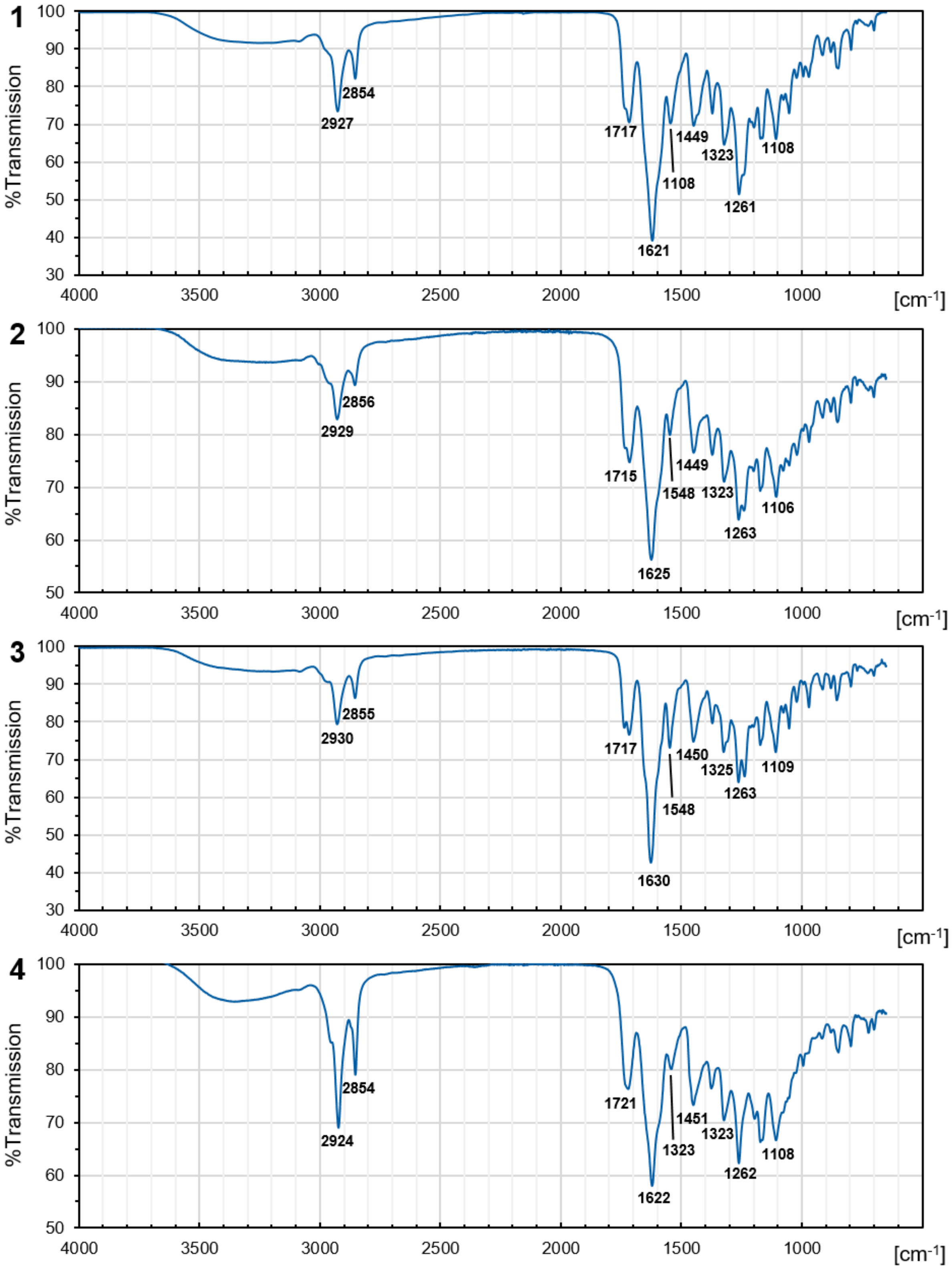

Figure S42: IR spectra (ATR) of hybridorubrins A−D (**1−4**) and fragirubrins F–G (**5**–**6**) from 4,000−650 cm^{-1}.

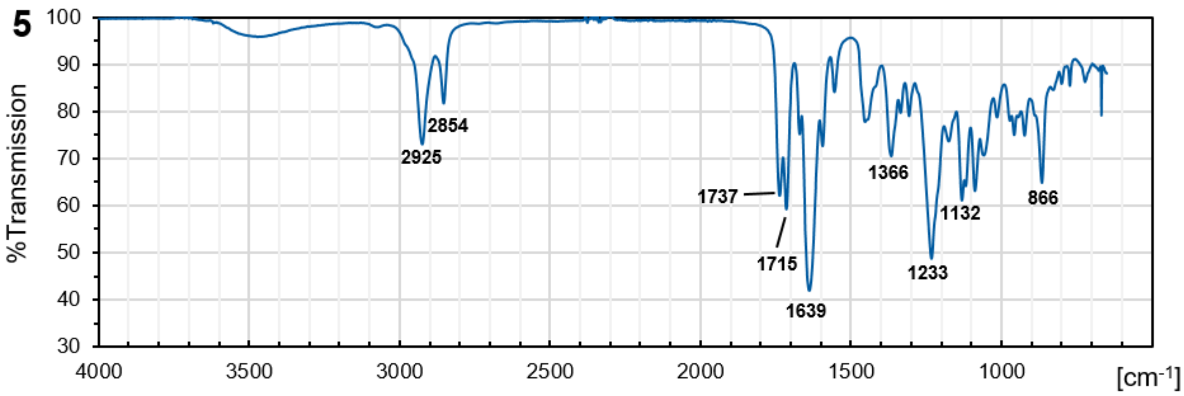

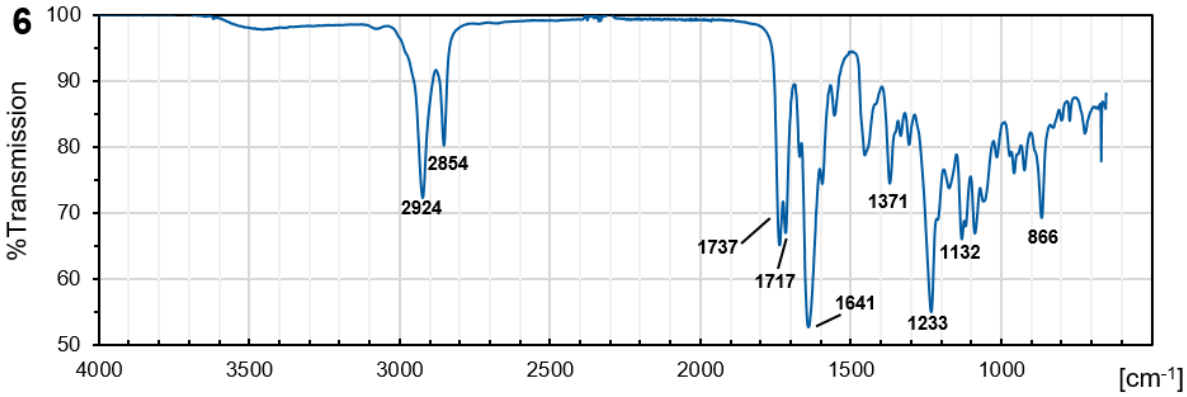

Figure S42 (continued). IR spectra (ATR) of hybridorubrins A−D (**1**−**4**) and fragirubrins F–G (**5**–**6**) from 4,000−650 cm^{-1}.

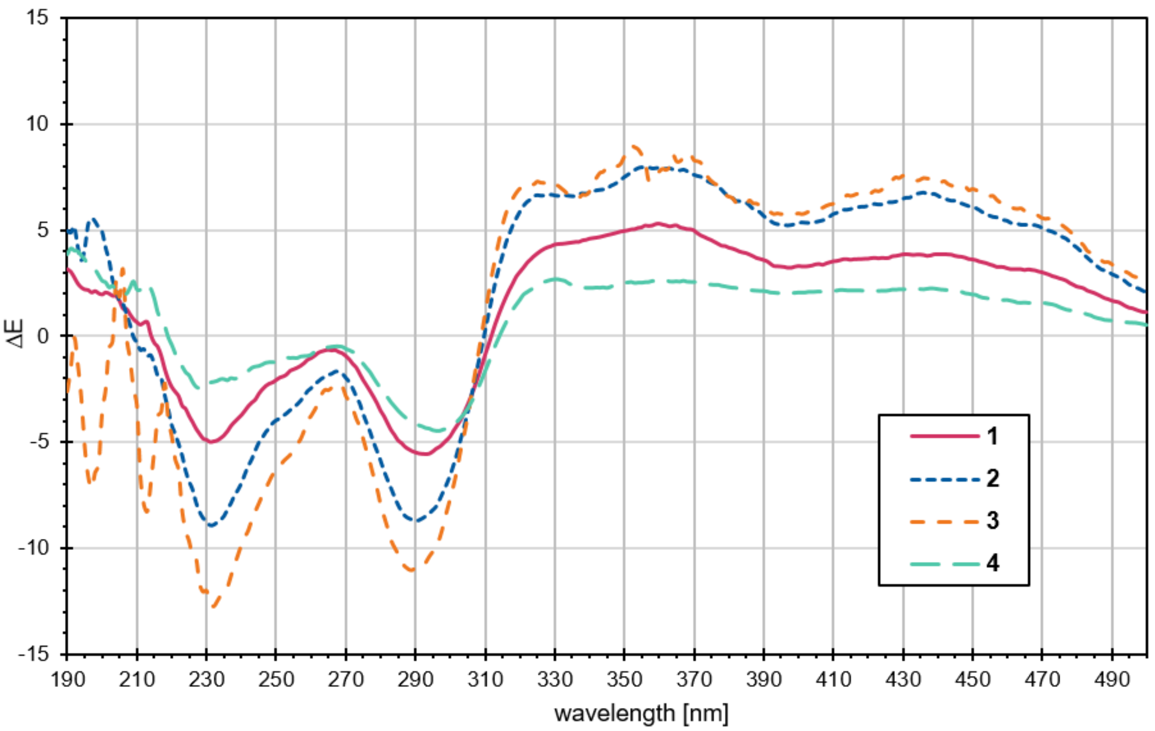

Figure S43: ECD spectra of hybridorubrins A−D (**1**−**4**) from 190−500 nm.

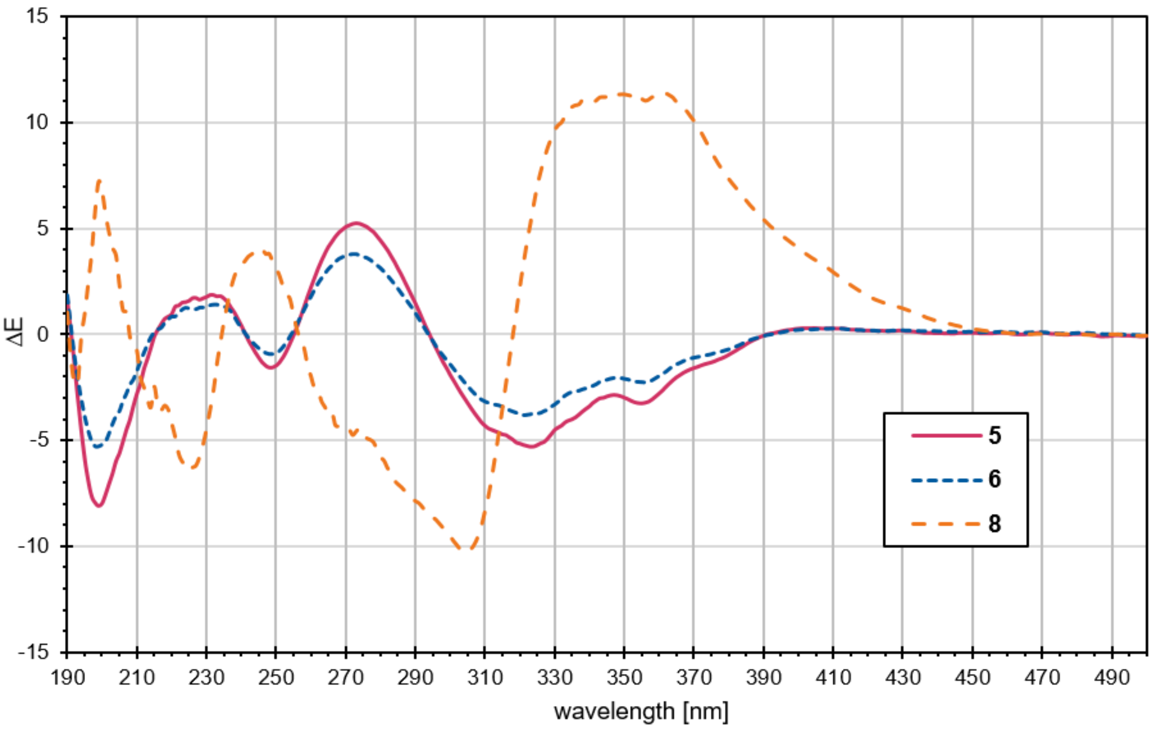

Figure S44: ECD spectra of fragirubrins F−G (**5**−**6**) and mitorubrinol (**8**) from 190−500 nm.

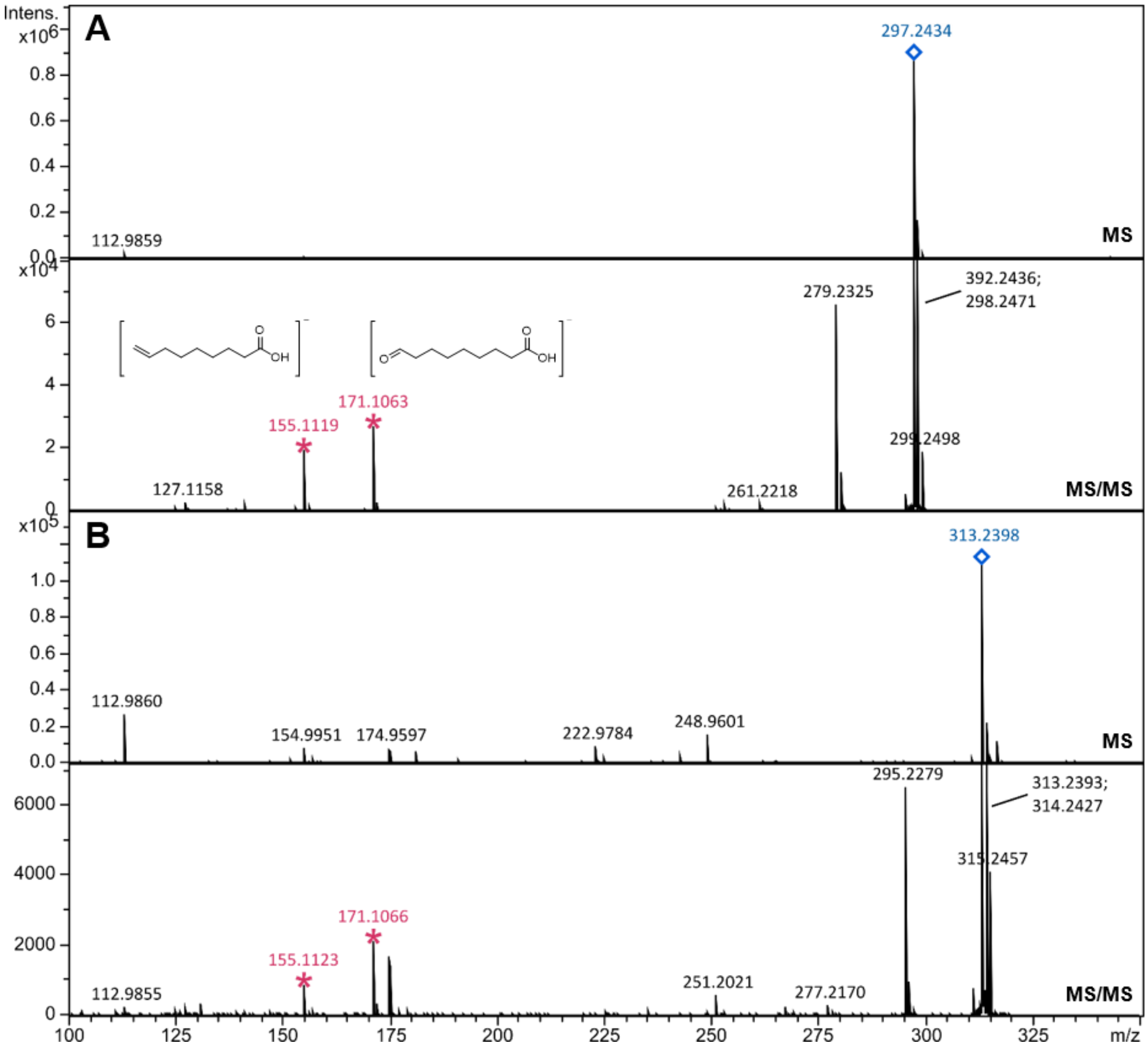

Figure S45: MS and MS/MS spectra between *m/z* 100−350 of compounds epoxidised with mCPBA for double bond position analysis.[S6]

A: epoxidised reference *cis*-octadecenoic acid, t_R = 17.13 min.

B: epoxidised sample of hybridorubrin C (**3**), t_R = 13.35 min. Due to partial hydrolysis of the ester bond linking the azaphilone core and the fatty acid in the sample, the *m/z* 313.2398 (indicating 17-hydroxy-octadeceneoic acid from **3**) was utilised as a parental ion.

Parental ions are highlighted by a blue diamond, while diagnostic ions used for determination of the double bond position are highlighted by pink asterisks.

[S6] Y. Feng, B. Chen, Q. Yu, L. Li, *Anal. Chem.* **2019**, *91*, 1791−1795

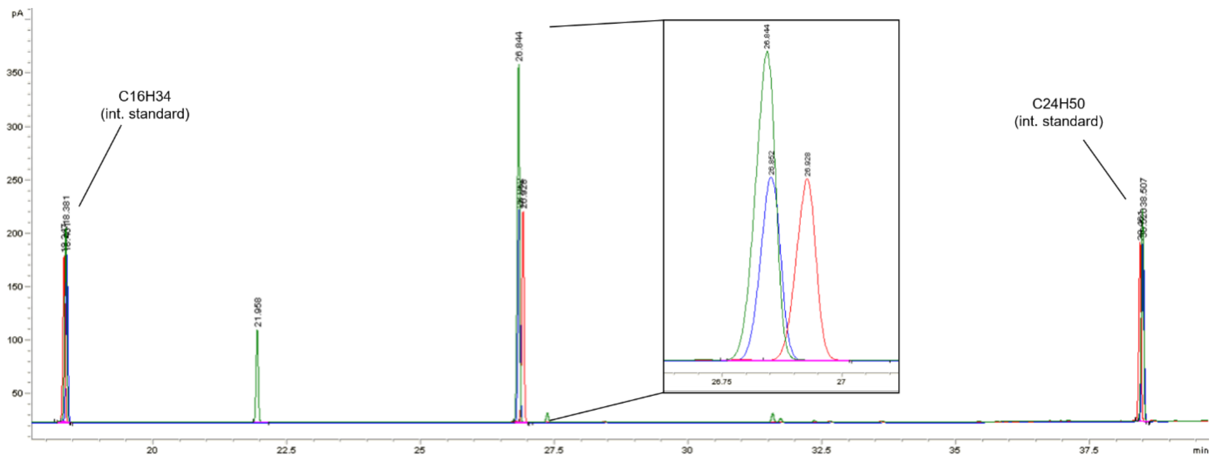

Figure S46: GC chromatogram of the fatty acid-methyl esters of fragirubrin G (**6**), 9-*cis*-, and 9-*trans*-hexadecenoic acid. Green trace: fragirubrin G (**6**)-FAME; blue: 9-*cis*-hexadecanoic acid-FAME; red: 9-*trans*-hexadecanoic acid-FAME.
